# “The architecture of the state was transformed in favour of the interests of companies”: corporate political activity of the food industry in Colombia

**DOI:** 10.1186/s12992-020-00631-x

**Published:** 2020-10-12

**Authors:** Melissa Mialon, Diego Alejandro Gaitan Charry, Gustavo Cediel, Eric Crosbie, Fernanda Baeza Scagliusi, Eliana María Pérez Tamayo

**Affiliations:** 1 Department of Nutrition, School of Public Health, University of São Paulo, São Paulo, Brazil; 2 School of Nutrition and Dietetics, University of Antioquia, Medellin, Colombia; 3 School of Community Health Sciences, University of Nevada, Reno, USA

**Keywords:** Commercial determinants of health, Corporate political activity, Food industry, Non-communicable diseases

## Abstract

**Background:**

In Colombia, public health policies to improve food environments, including front-of-pack nutrition labelling and marketing restrictions for unhealthy products, are currently under development. Opposition to these policies by the food industry is currently delaying and weakening these efforts. This opposition is commonly known as ‘corporate political activity’ (CPA) and includes instrumental (action-based) strategies and discursive (argument-based) strategies. Our aim was to identify the CPA of the food industry in Colombia.

**Methods:**

We conducted a document analysis of information available in the public domain published between January–July 2019. We triangulated this data with interviews with 17 key informants. We used a deductive approach to data analysis, based on an existing framework for the CPA of the food industry.

**Results:**

We identified 275 occurrences of CPA through our analysis of publicly available information. There were 197 examples of instrumental strategies and 138 examples of discursive strategies (these categories are not mutually exclusive, 60 examples belong to both categories). Interview participants also shared information about the CPA in the country. The industry used its discursive strategies to portray the industry in a ‘better light’, demonstrating its efforts in improving food environments and its role in the economic development of the country. The food industry was involved in several community programmes, including through public private initiatives. The industry also captured the media and tried to influence the science on nutrition and non-communicable diseases. Food industry actors were highly prominent in the policy sphere, through their lobbying, close relationships with high ranking officials and their support for self-regulation in the country.

**Conclusions:**

The proximity between the industry, government and the media is particularly evident and remains largely unquestioned in Colombia. The influence of vulnerable populations in communities and feeling of insecurity by public health advocates is also worrisome. In Colombia, the CPA of the food industry has the potential to weaken and delay efforts to develop and implement public health policies that could improve the healthiness of food environments. It is urgent that mechanisms to prevent and manage the influence of the food industry are developed in the country.

## Background

In 2019 in Colombia, public health policies to improve food environments, including the introduction of a new front-of-pack nutrition labelling (FOPNL) system and marketing restrictions for unhealthy products, were under discussion in the Congress [1–3]. This was in response to the increased burden of non-communicable diseases (NCD), such as diabetes, cardiovascular diseases and cancers, which now are responsible for 75% of all deaths in the country [4]. Unhealthy diets, in particular, are amongst the main risks factors for NCD [5]. According to the last Colombian National Nutrition Survey conducted in 2015, among children under 5 years old, 10.8% were stunted, while 6.4% were overweight [6].

Civil society organisations and the media reported that actors representing the food industry strongly opposed these policies [7–13]. The influence of the food industry over the media and in the Congress, was evident in 2016 during the proposal for an increase in the taxation of sugar-sweetened beverages, which has yet to be implemented in Colombia [11]. In this instance, the industry commissioned its own economic studies to counter evidence that an increase in the tax was needed to improve population health [11]. Food industry actors also developed several ‘corporate social responsibility’ initiatives in the country, through the support of communities, which could have helped improve its image in the public opinion [11]. The industry has continued to exert its influence, using similar practices, during the development of the Obesity Prevention Law n°019 of 2017 (Proyecto de Ley or PL019 de 2017) that included the development of a new FOPNL system and restrictions of the marketing on unhealthy foods to children [10, 13, 14]. An investigative report described the use of the ‘revolving door’ with employees from the food industry going to work in government [15].

These food industry actions represent ‘corporate political activity’ (CPA), which includes action-based, instrumental strategies (coalition management; information management; direct involvement and influence in policy; legal strategies) and arguments-based, discursive strategies, stressing the food industry’s importance in the economy, the potential costs associated with the implementation of public health policies, and framing the debate on diet-related health issues in ways favourable to its products and practices, with an emphasis on individuals responsibility and freedom of choice [16, 17]. These practices are described in Additional file 1. Scholars explain that CPA is not necessarily punctual and bound to specific periods of time, such as during the development of specific policies that might threaten the activities of an industry, but rather used to influence public health both in the short and long term [18]. CPA is part of a broader literature on the commercial determinants of health, which corresponds to the negative influence that corporations have on health [19–21].

There is limited research and a lack of monitoring of the food industry CPA in Latin America [22], including Colombia. A pilot study in the region showed that in the country, food industry actors emphasised their prominent role in the economy in order to counter criticism; they tried to demonstrate that they were part of the solution in the prevention and control of NCD; and they built alliances with health organisations and communities [23].

In the present study, our aim was to identify the CPA of the food industry in Colombia.

## Methods

We conducted a document analysis of publicly available information triangulated with interviews, conducted between May and August 2019. The study was led by an international researcher with expertise on the CPA of the food industry, based in Colombia during data collection and analysis, with working proficiency in English and Spanish. Some of the interviewees knew the researcher for her work in that space, but not personally. The research team also comprised three local researchers with expertise in public policy and food environments in Colombia and globally, and two international researchers, with expertise on food environments and industry political practices. All of the researchers for this project took a critical stance to the influence of corporations on public health policy.

In our study, ‘food industry’ included the manufacturers of food and beverage products, wholesalers, retailers, distributors, food service providers and producers of raw material, as well as organisations acting on their behalf, overtly or covertly, including trade associations, public relations firms, ‘philanthropic’ organisations, research institutions, and other individuals and groups.

This manuscript meets the COnsolidated criteria for REporting Qualitative research (COREQ) [24] (Additional file 2).

### Document analysis

For our document analysis, we used a protocol developed by INFORMAS (International Network for Food and Obesity/non-communicable diseases Research, Monitoring and Action Support) for identifying the CPA of the food industry [16]. These methods and the framework used for our data analysis have been applied in different countries in the Pacific, Europe and Latin America [23, 25–30].

Data collection and analysis was led by the first author, using Excel to manage the data.

INFORMAS suggests identifying the most prominent actors in a given country, in terms of market shares [16]. We did not have access to this information for the food industry and instead consulted with local experts and undertook a pilot study, as recommended by INFORMAS in such circumstances [16]: we visited the webpage of two global manufacturers that had national websites, Nestlé and Coca-Cola. This helped us estimate the level of information available on these webpages. Based on that analysis, we decided to include 20 food industry actors and data published between January–July 2019 in our study (purposive sample), except for annual report or other annual event, where the most recent data was included. The industry actors included in our analysis are presented in Table 1. We included the members of the International Food and Beverage Alliance (IFBA), as these are amongst the largest food and beverage manufacturers globally [31]. Other actors in our sample were local food and beverage producers, a retailer and three groups funded by the food industry.

**Table 1:** List of industry actors included in our analysis

Food industry actor	Headquarter	Main activity in the food and beverage sector
Coca Cola	Atlanta, USA	Beverage manufacturer
Danone/Alqueria	Cajicá, Colombia	Food and beverage manufacturer
Ferrero	Alba, Italy	Food manufacturer
General Mills	Minneapolis, USA	Food manufacturer
Grupo Bimbo	Mexico City, Mexico	Food manufacturer
Kellogg	Battle Creek, USA	Food manufacturer
Mars	McLean, USA	Food manufacturer
McDonald’s	Chicago, USA	Fast-food restaurant
Mondelez	Deerfield, USA	Food manufacturer
Nestlé	Vevey, Switzerland	Food and beverage manufacturer
PepsiCo	Purchase (New York), USA	Beverage manufacturer
Unilever	London, UK	Food manufacturer
Alpina	Sopó, Colombia	Beverage manufacturer
Colanta	Medellín, Colombia	Beverage manufacturer
Grupo Nutresa	Medellín, Colombia	Food and beverage manufacturer
Postobón	Medellín, Colombia	Beverage manufacturer
Grupo Éxito	Envigado, Colombia	Retailer
Asociación Colombiana de Ciencia y Tecnología de Alimentos (ACTA)	Bogotá, Colombia	Professional association in food and technology
Asociación Nacional de Empresarios de Colombia (ANDI)	Bogotá, Colombia	Trade association
ILSI Nor-Andino	Bogotá, Colombia	Research institution funded by the food and beverage industry

All data is available as Additional file 3.

As recommended by INFORMAS [16], the sources of information for our study included the industry’s own material, government material and data from other sources, including professional associations and universities. The sources consulted for our study are presented as Additional file 4. Mars, General Mills, Grupo Bimbo and Unilever had no national website or Twitter account.

Data analysis is described in the INFORMAS protocol and consisted of simultaneous identification and coding of data relevant to CPA, using an existing framework (Additional file 1) [16]. The third and fourth authors reviewed 10 and 100% of the data, respectively. Disagreement was resolved through discussion (not quantified).

Our manuscript reports on the different food industry CPA strategies, as indicated in our document analysis and interviews. We allocated a code starting with the letter A followed by a number to each example of CPA identified in our document analysis.

### Interviews

The aim of our interviews was to get access to key informants who have a first-hand experience of food industry CPA, with no specific time limits or restrictions on the type of industry actors. The examples of CPA shared by participants helped triangulate data that we found in the public domain. They also identified additional examples, as detailed in the results section. Moreover, during the interviews, participants shared their perspectives and opinions about food industry CPA in Colombia and globally.

The first author conducted 13 semi-structured interviews, including two group interviews. In total, 17 key informants participated in our study, from the legislative branch of the government (*n* = 1), the executive branch of the government (n = 1), academia (n = 1), civil society (*n* = 12), and the media (*n* = 2). One person from academia accepted our invitation but was traveling and therefore could not be interviewed. We conducted our interviews until data saturation (i.e., when no new theme/CPA practices were identified by the first author). Sampling was purposive and participants identified through their discussion of food industry CPA in Colombia in the media. We also used a snowball sampling technique (participants invited potential interviewees from their networks). The interview guide is available as Additional file 5.

Participants were contacted by email or phone calls and offered to participate, voluntarily and under strict conditions of anonymity and confidentiality, in the study. An ethics agreement was signed between the interviewer and the participants. Participants consented with field notes being taken and the interview being digitally recorded. They had the opportunity to revise their transcript before the submission of this manuscript. At this stage, one participant asked for most of the information shared during her interview to be deleted, for fears of reprisals. One participant withdrew from the study at the peer-review stage during the publication of the present article, after other articles on the CPA of the food industry in Colombia were published. We have not counted her in our list of participants.

Interviews lasted 1 h on average; were conducted face-to-face (*n* = 12) or through Skype (n = 1); in Spanish (*n* = 10), Spanish/English (*n* = 3) and French (n = 1). Interviews were transcribed verbatim by a contracted translator under the condition of confidentiality.

Data analysis was led by the first author and used the existing framework presented in Additional file 1. The second and last authors reviewed 10 and 100% of the data for the interviews, respectively. We used Word and Excel to manage the data.

All information that could identify our participants has been removed from this manuscript and generic terms are used to describe their professions, with no number allocated to each participant, to preserve their anonymity and confidentiality. We use ‘she/her’ when referring to both male and female participants. A translation of the present article is available as Additional file 6.

## Results

We identified 275 occurrences of CPA between January–July 2019 through our analysis of publicly available information. Table 2 is a summary of the examples we found in the public domain, classified by industry actor and by CPA strategy.

**Table 2:** CPA strategies used by the food industry in Colombia in 2019 (categories are not mutually exclusive)

	Instrumental strategies				Discursive strategies
	Coalition management	Information management	Direct involvement and influence in policy	Legal strategies	
**Food industry actor**					
Coca Cola	4	4	2	0	12
Danone/Alqueria	12	8	1	0	6
Ferrero	0	0	0	0	0
General Mills	0	0	0	0	0
Grupo Bimbo	0	0	0	0	0
Kellogg	1	1	0	0	0
Mars	1	0	0	0	0
McDonald’s	5	3	0	0	1
Mondelez	0	0	0	0	0
Nestlé	7	22	0	0	14
PepsiCo	1	2	0	0	2
Unilever	1	2	0	0	0
Grupo Nutresa	13	5	1	0	13
Postobón	11	2	1	0	29
Colanta	8	3	0	0	0
Alpina	0	3	0	0	0
Grupo Éxito	24	9	0	0	18
Asociación Colombiana de Ciencia y Tecnología de Alimentos (ACTA)	1	3	1	0	2
Asociación Nacional de Empresarios de Colombia (ANDI)	9	11	7	0	21
ILSI Nor-Andino	2	15	0	0	0
Multiple actors for the campaigns “Decido lo que como” and “Bebidas de tu lado”	1	13	2	0	21

We identified 197 examples of instrumental strategies and 138 examples of discursive strategies. These categories are not mutually exclusives and 60 occurrences belong to both CPA strategies. Participants in our interviews also identified example of CPA, describing actions or arguments that have been used in the past few years by the food industry in Colombia. The CPA strategies used by the food industry with regards to the discussion on the introduction of a new FOPNL system in Colombia is the subject of a separate publication [32].

### Coalition management: building alliances and weakening the opposition

In Colombia, we identified 101 examples from our document analysis of the coalition management strategy. Additional examples were shared during the interviews. As part of this strategy, the food industry established relationships with health organisations, communities, and the media and with other industry actors. In parallel, it used different mechanisms to weaken its opponents, as described below.

#### Capture of the media

Interview participants noted the capture of the media in Colombia, where the Ardila Lulle Group owns a leading TV channel, RCN, and the beverage company Postobón. This ownership led to two cases of censorship of public health campaigns in Colombia, as described in the section ‘legal strategies’, below.

*“RCN [a TV channel] belongs to an economic group called Ardila Lulle. And the group Ardila Lulle has the largest soda company in Colombia, which is called Postobón. (...) the dominant media in Colombia all have a relationship with the food industry … a direct ownership relationship; the food industry owns the media.” [public health advocate]*

*“The newspapers, the radios, the most important TV [channels], are bought by groups of industries who have a huge amount of companies in the food industry. This is one of the reasons why the [public health] ads on TV and radio were immediately censored (...). It perfectly reflects the fact that the media belong to the economic groups to which the food industry is also part of.” [public health advocate]*

#### Interactions with civil society and health organisations and involvement in the community

Several companies have their own charities in the country. The Fundacion Éxito collaborated with several actors in the food industry, including Coca-Cola, and with city councils, the Ministry of Health, the Instituto Colombiano de Bienestar Familiar (Colombian Family Welfare Institute), the Department of National Planning and the Office of the Inspector General of Colombia [A84]. The Fundacion Nutresa counted on the support of the Ministry of Education of Colombia, UNICEF and the World Food Program [A217]. One participant in our interviews also described a government programme which involved food industry employees:

“*There is a program [organised by the Instituto Colombiano de Bienestar Familiar or Colombian Family Welfare Institute, ICFBF] for food and educational support for families with children under two years of age, called “La modalidad familiar” (“The family modality”). (...) These programs must hire professionals to take care of the children. In some occasions the training for the professionals is done by Nestlé or Alpina. The official training of the ICBF.*” [public health advocate]

Table 3 presents other initiatives that were funded or supported by the food industry in Colombia during the period of analysis.

**Table 3:** Initiatives funded or supported by the food industry in Colombia in 2019

Company	Details about initiatives supported by the industry	Main area of the initiatives					Reference from sup material 5
		Physical activity	Nutrition	Education/schools	Children	Other	
ANDI	Refurbished a school with the support of the Colombian army			X	X		A9
Alpina, Coca-Cola	Developed a programme of food education		X				A54
Coca-Cola	Developed “Vive bailando” (“Live dancing”), a physical activity programmes	X					A43
	Developed “Ludonutricion”, a programme based on games to inform about nutrition, including through the support for “Juego y Niñez”, a charity that promote playing among children		X	X	X		A45–6, A48–9, A136
	Developed “Apúntate A Moverte” (“Sign up to move”), a physical activity programme	X					A50
	Supported the Fundacion Éxito (from the supermarket chain Éxito)					X	A83
Colanta	Provided furnitures to an educative institute calles “IE Tricentenario” in Medellin			X	X		A56
	Participated in the event organised by the city of Sonson for the month of the children				X	X	A57
	Developed “Colanta Sabe Más, Sabe a Campo” (Colanta knows more, knows the field), where they distributed notebooks to kids			X	X		A58–9
	Supported “Vaso de leche” (“glass of milk”) set up by the city of Medellin to distribute milk to children in schools		X		X		A60
	Supported “Programa Maná” from the government of the State of Antioquia, where 140,000 children are “given a daily serving of flavoured milk powder, as a nutritional supplement candy, which can be consumed directly or diluted in water”		X		X		A60
	Supported the “Desayunos Infantiles” (“children breakfasts”) from the ex-President Álvaro Uribe, where children are served with a “portions of liquid flavored milk and a fortified cookie”		X		X		A60
	Distributed “55 million litres of milk in Colombian capitals such as Bogotá, Medellín, Cali, Barranquilla, Armenia. Pereira and Manizales.”		X				A60
	Organised the “Festival Colanta” with the participation of the head of the Department of Education and Cooperative Promotion				X	X	A62
Danone/Alqueria	Distributed products to “Granitos de Paz”, a charity that support the development of children (health, education, etc.)		X		X	X	A67
	Distributed products to “Alimenta Compartiendo” (“Feed sharing”), a programme that distributed “1.5 million glasses of milk to 5200 children distributed through 14 food banks”		X		X		A68
	Participated in the activities of the “Fundación La Esperanza en La Calera” on environmental protection, together with the food bank of Bogotá					X	A72
	Distributed products to “Programa Desayuno Saludables” (“Healthy breakfast programme”), where they distributed 11 t of milk to “3400 children in vulnerable situations”.		X		X		A73
	Partnered with food banks in Colombia and the Fundacion Saciar during the World Milk Day on 1st of June and distributed 1,500,000 glasses of milk in the country		X				A75–7
ANDI, Danone/Alqueria, Éxito	Actors from the food industry launched in 2019 the “Alianza por la Nutrición Infantil” (“Alliance for Child Nutrition”) in partnership with multiple actors from the public sector, to “favour the rights of children, calling attention to the fundamentals that start from nutrition, as the axis of physical, emotional and cognitive development of human beings”		X	X	X		A78, A82, A89–97, A101–2
ANDI, Coca-Cola, McDonald’s, Mars	Supported Concordia, an “organisation dedicated to actively fostering, elevating, and sustaining cross-sector partnerships for social impact”					X	A137
Éxito	Supported the musical initiation programme from the symphonic orchestra of Antioquia					X	A85
	Supported the San Vicente Fundación for its programme of nutrition for children in hospitals		X		X	X	A86
	Supported pregnant mothers and children that were part of an official food assistance programme, through the support for human milk banks and food banks		X		X	X	A87
	Participated in the development of the “Comprehensive Care Guide for chronic malnutrition” with the city of Bogotá, the Fundacion Santa Fé de Bogota and the Instituto Colombiano de Bienestar Familiar (Colombian Family Welfare Institute)		X				A98
	Participated in a public-private partnership between the Ministry of Health, the Fundacion Santa Fé de Bogotá and Éxito for the Day of Human Milk Donation		X		X	X	A99
	Developed its “Cen Zero” (“generation Zero”) strategy “to ensure that no child under 5 suffers from chronic malnutrition in Colombia by 2030”		X		X	X	A104, A107
Kellogg	Supported “Juego y Niñez”, a charity that promote playing among children				X	X	A136
McDonald’s	Ran its Mc Lectura feliz, a programme to promote reading among children				X	X	A131–3
Multiple actors	In 2009, Alquería, Éxito, Nutresa, Unilever and ANDI founded the Association of Food Banks of Colombia (ABACO)		X			X	A139
Nestle	Ran its “Unidos por Niños Saludables” (“United for Healthy Children”), with the support of different actors, including the Colombian Heart Foundation, the Colombian Society of Pediatricians, the charity Juego y Niñez		X	X	X	X	A177–81, A185, A204–5
	Supported “Juego y Niñez”, a charity that promote playing among children				X	X	A136
	Celebrated the Breakfast Day in different cities and the Children’s Day, including through the organisation of a workshop on cooking and nutrition with children and their parents in a school		X		X		A183, A193, A195, A201
Nutresa	Participated in clean-up events					X	A206–7
	Supported food banks		X			X	A208, A211–2
	Provided a school dining room to the Social Pastoral Secretariat			X	X		A212
	Supported several charities: • the Fundación Bambi • The Institución Educativa San Vicente: supported the First National Education Congress • Fundación Victor Salvi: support the Cartagena Festival Internacional de Música • The charity Secretos para Contar • Fundación Empresarios por la Educación: covered the Annual Fee for School Management Improvement • Corporación Pueblo de los niños • Fundación La Cueva: supported the International Arts Carnaval • Fundación Notas de Paz: supported the Infants and Children Philharmonic Orchestra • Fundación Sura Becas • Teatro Metropolitano: supported the International Classical Music Season • Fundación Soleira: supported the Observatory on human rights of childhood and adolescence • Alianza Colombo Francesa: supported the Music Festival • Teatro Metropolitano Sillas: sponsored 8 seats from the theatre • Donación Proantioquia: supported a Prize for Education			X	X	X	A212–3
	Ran its “Disfrutar la vida te alimenta” strategy (“Enjoying life feeds you”), based on “four fundamental pillars: physical activity, balanced eating, sharing with the family and enjoying the outdoors”.	X	X	X		X	A222
	Ran its “Nutresa Quiere a los Niños” programme (“Nutresa Loves Children”), to teach children about healthy diets and lifestyles	X	X	X	X		A229
Pepsico, Postobón	Supported “Fuente de Vida Malambo”, a charity that brings potable water to the population in Malambo					X	A230
PepsiCo	Ran its “Aliméntate y Actívate” programme (“Feed and Activate”), to teach children and their parents about healthy lifestyles	X	X	X	X		A234
	Ran its ““Nutrición para el future” programme (“Nutrition for the future”), that aims to “provide access to at least 3 billion servings of food and nutritional drinks for marginalised communities and consumers around the world”		X				A234
Postobón	Ran its “‘Mi pupitre Postobón” programme (“My Postobón desk”), where the company distributed desks made from recycled containers of Postobón beverages in schools across Colombia			X	X		A235, A238, A244, A246–7
	Ran its “Mi Bici Postobón” programme (“My Postobón bike”), where the company offered Postobón bikes to children in schools across Colombia	X		X	X		A237, A239–41, A245, A251–2
	Supported “Agenda del Mar”, a charity that works on environmental protection					X	A248
	Launched its “¡Boom! Activa tu vida” programme (“Boom! Activate your life”) with the support of the city council of Medellin to promote healthy lifestyles	X					A249–50, A2755

Information contained in Table 3 demonstrates the extent to which the food industry is present in communities in Colombia. It also presents the many interactions between food industry actors and the public sector. In addition, most of the community programmes sponsored or supported by the industry targeted children and focused on education, nutrition and/or physical activity.

Several participants in our interviews described a case, from 2017, where Postobón launched a programme in a desert region in the far north of Colombia, la Guajira, where the company distributed, on a daily basis, two beverages fortified with micronutrients to children, for free [33]. Our interviewees explained that the industry planned to commercialise these beverages in the rest of the country and said that Postobón started a study with these children, with no approval from an independent ethics committee, taking blood samples and anthropometric measures [33]. Our participants also discussed about the industry efforts to get the support of the Ministry of Health for this programme, which never happened. This story became a scandal in the media in early 2018, but a recent investigation found that, almost 2 years later, Postobón is still running its programme in different communities in Colombia [33, 34].

*“When the scandal arose, they had already started changing their discourse, as I told you, and they were saying they never had a commercial interest in [developing these products]. And in the end they never went on the market and it was removed from the school programs.” [member of the government]*

These interactions could be detrimental to public health, particularly when the programmes are heavily branded or when the industry is distributing products that might not be healthy. For example, Colanta, Nestlé, Nutresa and Postobón used marketing material with their brands on it when organising events in schools and/or the community [A58–9, A185, A193, A216, A241]. In addition, Colanta through the “Programa Maná”, from the government of the State of Antioquia, distributed “a daily serving of flavoured milk powder, as a nutritional supplement candy, which can be consumed directly or diluted in water” to 140,000 children [A60]. Our participants were critical of these community programmes:

*“[Schools] are receiving funds and are validating the presence of the industry in school environments, which should be protected from precisely [these] brands, and should be protected from the availability of those products ( …*). *In fact it is brand placement what they are doing.” [public health advocate]*

*“That is super bad, because then [food industry actors] are reaching the most vulnerable populations, trying to be in a certain way the saviour. And that in fact gives them a power to have vulnerable population in their favour that can directly defend the interests of the industry. And [they are] even coming with the State, who validates them with greater force.” [public health advocate]*

Finally, these programmes could help the industry get privileged access to policy makers:

*“What they do with all these programs in several territories is basically to create strategic alliances with local civil society actors and decision makers that ultimately creates a support base for them.” [public health advocate]*

It is crucial to note that Colombia is a unique case, in the sense that it has a history that has been marked by an armed conflict [35]. Moreover, many segments of its population, including indigenous and afro-descendants, are still marginalised and lacking access to basic infrastructure, food and education [36]. As such, information published in the public domain (Table 3) and shared by participants suggest that the involvement of the food industry in the community is often seen as a contribution to peace, joy, social development and prosperity for the country (this is also described in the ‘discursive strategies’ section below). The industry sometimes fills a gap where the government has been absent.

*“Some children didn’t go to school before and now go to school. What are you going to say? Well, is this wrong? Is it better not to go to school? But then it is a vacuum of the State. The State is out-sourcing a series of services that are under its responsibility to the private sector. That is where the problem lies. ( …) Then you use the industry to act as charities, no?” [journalist]*

This position was however critised by some of our participants:

*“The government has no money, simply because the government, which was captured by companies, does not collect taxes from companies, does not collect money from its returns on investments ( …). The government instituted large free trade zones in the country, where they can import their raw material without any cost. Of course, the state has no money ( …). Because all the architecture of the State was transformed in favour of the interests of companies.” [public health advocate]*

#### Constituency fragmentation and destabilisation

Paradoxically, defending the human rights to adequate standards of living, including the right to food, and promoting the prevention and control of NCD, often exposed individuals, particularly those from civil society, to threats and dangers in Colombia. This was described in a New York Times article in 2017 [37], when the director of the consumer organisation Educar Consumidores received direct threats, although no direct links with the food industry were made at that time. Public health actors in Colombia felt unsafe on a daily basis. Some had their equipment stolen, including material with sensitive information.

*“We feel unsafe yes ... we must, because we have realised that there are many people ( …) who are behind each person to see what they write on the cell phone ( …) when the plenary sessions are large, we have noticed that they take pictures with cameras with such a [small lens], without lying to you, without exaggeration.” [public health advocate]*

### Information management: influencing science

The food industry used different practices to try to influence the production and dissemination of information regarding public health nutrition in Colombia. We found 99 examples in this category in our document analysis. This strategy was also discussed during the interviews.

#### In-house production and amplification of research

Actors in the food industry directly undertook research and disseminated information about nutrition in Colombia. Nutresa had its own research centre on non-communicable diseases and their links with diets, called Vidarium (where “Vida” means life) [A226, A228]. Nestlé ran its nutrition programme around Colombia, “Unidos por Niños Saludables” (see Table 3), where it disseminated information to children, parents and teachers [A201, A204–5]. The company collaborated with the Faculty of Nursing and Rehabilitation of the University of La Sabana for a validation of this programme [A188]. The results from the study then served to further promote the programme [A188]. The Foundation Éxito gave a Child Nutrition Prize to “*public and private institutions from different sectors [who] act to improve the nutrition of children in their first 1,000 days of life*” [A112]. In addition, the Foundation Éxito organised an event in May 2019 where it met with “*some of the most important media in the country talking about the importance of nutrition for brain development*” [A100]. Coca-Cola organised a series of talks “*aimed at government entities, in which we provide information on the energy balance and adequate hydration, contributing to the promotion of active and healthy lifestyles. To date, we have benefited more than 900 people*.” [A55].

The ‘Alianza por la Nutrición Infantil’ (‘Alliance for Child Nutrition’), a public private initiative (see Table 3) launched in 2019 by the Foundation Éxito, organised, in partnership with the Ministry of Health, offered different courses to health professionals on infants and young children feeding and epidemiology, where those professionals received an official certification from the government [A96–7].

One participant in our interviews explained that the industry also often pays for the travel and fees for students and academics to attend these conferences [academic]. Another participant explained that professional associations, like the Asociación Colombiana de Dietistas y Nutricionistas (Colombian Association of Dieticians and Nutritionists, ACODIN), invite food industry representatives in their congresses:

*“The inaugural keynote address of the [ACODIN congress a few years ago] was [delivered] by Jairo Romero [from the “Latin American Association of Food Science and Technology”, ALACCTA]. (…). Obviously the event was full of industry exhibition booths, wasn’t it? (…). ACODIN, the association of nutritionists is completely co-opted by the industry.”* [public health advocate]

#### ILSI nor-Andino

ILSI Nor-Andino is the local branch of the International Life Science Institute, an industry front group that has been criticised for its influence on science and policy in numerous countries [38–40]. Alpina, Coca-Cola, Kellogg, Mondelez, Nestlé, Pepsico, Postobón and Unilever were all members of ILSI Nor-Andino as of August 2019 [A115]. In Colombia, a newspaper article described the many ways in which ILSI influences policy and research in the country [41]. ILSI collaborated with the Ministry of Health and academics from different universities, without necessarily disclosing its links with the food industry [41]. These individuals in turn participated in policy making without disclosing these links with ILSI and the industry [41].

ILSI’s board members include a retired professor from the Universidad Nacional de Colombia, a professor from the Pontificia Universidad Javeriana and employees from Nestlé and Alpina, among others [A114].

In our interviews, participants discussed a research project on diet and physical activity in Latin America, called the ‘Estudio Latinoamericano de Nutrición y Salud’ (Latin American Study of Nutrition and Health, ELANS). ELANS is funded by Coca-Cola and ILSI, among others, and is led in Colombia by researchers from the Pontificia Universidad Javeriana [42].

ILSI and some of its industry members supported different scientific events to disseminate information about nutrition in Colombia in 2019 [A120, A125]. ILSI and Unilever sponsored the annual congress of ACODIN [A160]. Unilever and Kellogg’s sponsored some of the ACODIN sessions [A129, A161]. ILSI, Danone and McDonald’s sponsored the annual congress of the Asociación Colombiana de Nutrición Clínica (Colombian Association of Clinica Nutrition, ACNC) [A162]. During the congress, ILSI organised a session on infant nutrition [A162].

#### Influence on science translates into political influence

In our interviews, it was suggested that the influence of the food industry on science in Colombia could also directly translate into political influence.

*“A professor at [the University of] Los Andes ( …) has worked a lot on sports but with [funding from] Coca-Cola. (...) He has sabotaged several [public events about nutrition and health], he is one of the strongest academic detractors in Colombia. (...) For example, he never appears in public hearings, ( …) but he is very close to the current Minister, he appears in academic debates ( …). And for him his conflict of interest is with Coca-Cola. That never gets mentioned.” [public health advocate]*

Participants in our interviews explained how the food industry tried to shape the evidence in Colombia during the discussion for an increase in the sugar-sweetened beverages taxation in 2016/2017:

*“They hired two people to do two studies, two very well-appointed people in the country. ( …). And each one made a separate study ( …) and they came to say in a public event that if the tax on sugary drinks was implemented, the mothers and parents, as a substitute [to sugar-sweetened beverages] were going to put beer in the lunchbox of their kids. [The studies] were never peer reviewed, we never saw them published in an indexed scientific journal, they never even published [them].”[public health advocate]*

### Direct involvement and influence in policy

The food industry is a prominent and influential actor in public health policy in Colombia. We identified 16 examples of this practice during data collection of publicly available documents. Our participants in the interviews also described examples in this category.

#### Lobby

Several participants in our interviews described the lobbying exerted by the food industry in the Congress:

*“They manage to co-opt the new members of parliament who arrive ( …) and what you see is that they start visiting them, they start asking for appointments ( …). Then there, the industry frequently asked for appointments to then speak, express their interests.” [politician]*

*“They enter the congress and go everywhere without any legal authorisation; then they get into the organisation of the plenary’s agenda, help break the quorum of the plenaries, pass proposals to be signed by someone to block, to file bills, to change the articles of the bills. They get into the whole parliamentary process irregularly.” [politician]*

One interviewee described how different food industry actors join forces and build alliances within the industry to then influence policy in Colombia.

“*One strategy they use is to leverage in the associations ( …) So they are not lonely voices of an industry saying something but they are unionised voices.” [public health actor]*

The President of Colombia, then a Senator, lobbied against a proposal to increase taxes on sugar-sweetened beverages a few years ago when he was a Senator [A141]. In 2019, he participated in and gave the final speech at the ANDI Bogotá section assembly, an event publicised on his official website [A17]. Our interview participants suggested that these interactions between the President and the industry have direct influence in policy in the country:

*“You end up with the positions - at least of this government - the positions that several of the officials who are in the government have today, starting with President Duque, who was a defender, so that the tax on sugary drinks was not developed. Well, no doubt that the government lobbyists will be in alliance with industry lobbyists.” [politician]*

#### Donations and other incentives

Information about political donations is difficult to retrieve from publicly available information in Colombia, as one would need to search information for every single individual in the government to learn about these donations. This therefore would form a separate study. One participant summarised the situation in Colombia:

*“Many years ago ( …) I talked to a politician and he said: “I don’t want to be a politician anymore because to be a congressman you have to sell yourself to an armed group that finances you or to a group of businessmen. Then every time you go to make a decision they always send someone who touches your shoulder and says: “remember that X sent you greetings, then you and X wants this not to be voted.” [public health advocate]*

It was reported in an investigative article that the food industry made numerous donations during the last presidential elections of 2018 [7]. The President of Colombia for example declared receiving the equivalent of US$148,000 from the sugar-sweetened beverages industry during his election campaign in 2018 [7].

Some interviewees explained that the food industry also offers gifts to politicians:

*“And the other way is that the industry always visits to bring presents (...) they give away objects. For example, they give away pens, ( …) bring wines, fine liquors, well-presented sweets, well-presented chocolates.” [politician]*

*“Individuals who came from the industry to the Congress talked with the congressmen and offered them. “Does your child want to go to study at such a university? Ok, senator or member of the parliament, we cover the costs of studying on the other side of the world for your son. You need such a thing. Ok, member, senator, we give you this, but you cannot vote for this.”[member of the government]*

#### Actors in decision making and self-regulation

Actors in the food industry often directly participated in policy making and other high level meetings in Colombia and internationally. ACTA for example declared working with the Ministry of Health regarding the reduction of salt intake in the Colombian population [A2]. In April, Colanta participated in the launch of the “Alianzas Competitivas para la Equidad” (“Competitive Alliances for Equity”), which aims to boost the development of the country through investments from corporations, in the presence of the President Duque and the ambassador of the USA in Colombia [A66].

Self-regulation, which indirectly affects the decision making process, by suggesting that other alternatives than mandatory regulation are possible, was also favoured by the industry and supported by the government [A257, A273]. The initiatives promoted by the food industry were: the provision of nutrition information to consumers [A152–3]; what they called “conscious advertising”; responsible marketing [A155]; a reformulation strategy [A12]; the promotion of healthy lifestyles [A12]. In the interviews, several participants were sceptical of this approach:

*“The self-regulation agreement (...) was adopted to avoid a tax on sugary drinks and avoid a series of proposals for state regulation that were about to be made at that time.” [journalist]*

### Legal strategies

We did not find information related to CPA legal strategies in our document analysis. However, participants in our interviews described two cases where public health campaigns ran by charities were challenged in the court.

The first case occurred in 2016, when Educar Consumidores ran a TV campaign about the negative health effects associated with the consumption of sugar sweetened beverages [12]. One participant detailed:

*“Educar Consumidores, when it tried to air a commercial on Colombian television about the health risks associated with the consumption of sugary drinks, immediately Postobón, which belongs to Ardila Lulle, sent a communication to the Superintendence of Industry and Commerce to withdraw the television commercial.”* [public health advocate]

As a consequence, Educar Consumidores had to stop its campaign [12]. Eventually, the Constitutional Court of Colombia recognised that Educar Consumidores had a right to share this information as it had important consequences for population health [12]. A participant in our interviews explained that the wife of a judge from the Constitutional Court which was in charge of the case got hired by Postobón during that period [public health advocate].

The second case happened in 2018, when the charity Red Papaz, which advocates for the protection of rights for children and adolescents, tried to run a campaign called “Don’t eat more lies” (“No comas mas mentiras”) [43]. The objective of the campaign as to disseminate information about the consumption of ultra-processed foods and its risks on health, particularly for children and adolescents [43]. Red Papaz wanted to run the campaign on the main TV channels in the country, but its request was rejected by the National Private Channels Consortium (Consorcio de Canales Nacionales Privados), which includes RCN [43]. The case was brought to the Constitutional Court in 2019 and eventually won by Red Papaz [44].

One participant explained that litigation against these types of campaigns was a well-known practice:

“*The SLAP [Strategic lawsuit against public participation] is a strategic litigation to deter or distort the debate. Industries sometimes initiate litigation strategies, not necessarily to win them, because they know they will not win, but to silence a voice or to frighten civil society*”. *[public health advocate]*

### Discursive strategies

We identified 138 examples, in the public domain, where the food industry used a diverse range of arguments as part of its CPA discursive strategies. Participants in the interviews also described discursive strategies.

#### Role of the industry in the economy

In Colombia, the creation of jobs by the food industry was often framed as a contribution not only to the economy [A232], but more importantly as a central factor for social development. This was sometimes discussed as part of the corporate social responsibility initiatives of food industry actors [A27]. The ANDI for example declared: *“The food industry in Colombia is an engine of economic and social development: Large generator of formal employment (260,000 workers); More than 65,000 companies; Large exporter: More than USD900 million to 129 countries. We create economic and social well-being!”* [A25].

The food industry also used the economic argument to criticise proposed public policies that would impact its products and activities. Following the suppression of a subsidy on sugar-sweetened beverages, Coca-Cola said that it lost revenues and had to cut 177 jobs, and as a consequence of these losses of money, the company decided to stop sponsoring the Colombian soccer team and declared that the decision had “*counterproductive effects for the economy*” [A44].

#### Framing of the debate in public health nutrition

In their efforts to frame the debate in public health nutrition in the country, food industry actors promoted their central role and efforts in the prevention and control of NCD and other diet-related issues. For example, Alqueria explained that its distribution of products to food banks was crucial for the country: “*We are aware of the importance of our role in the food chain and of our commitment to eradicate hunger in Colombia*” [A71]. Other actors presented similar arguments: *“Nestlé has contributed to improving the quality of life and ensuring a healthier future for children”.* [A186].

Food industry actors advocated for self-regulation, including for the use of a FOPNL system, as described earlier, and for other voluntary initiatives, including the promotion of education about nutrition and physical activity, instead of the introduction of new public policies [A29, A33–4, A196, A256, A273].

We identified two initiatives of the food industry: each had a dedicated website and a dedicated Twitter account. The first was “Decido lo que como” (“I decide what I eat”) [A38, A47]. The initiative was developed by the Foundation Éxito, Nestlé and other actors in the food industry [A138] and the sources of information cited were industry actors [A203]. The second initiative was “Bebidas de tu lado” (“Beverages on your side”) where the ANDI promoted the five self-regulatory initiatives adopted by food industry actors in Colombia, as described earlier [A12, A150]. In their messaging, on these platforms and other media, food industry actors particularly promoted personal and parental responsibility, balanced diets and physical activity [A47, A49, A52–3, A149, A156, A200, A254, A259–67, A274 and interviews].

In our interviews, one participant suggested:

*“In a country like Colombia, which is the victim of an internal conflict that has not ended and has already lasted for over a century; this issue of [personal] blaming has a lot of power.” [public health advocate]*

## Discussion

The results of our study reveal that the food industry is a prominent and influential actor in Colombia. In our study, we found 275 examples of CPA practices for the food industry, using publicly available data. Our participants described additional examples, including new data about legal strategies, and provided a critical analysis of these actions and arguments of the food industry.

We found evidence that food industry actors built alliances with communities, the government (national and local) and the media. The interactions between the food industry and actors in government, academia and the media, amongst others, could mean that the industry gets credibility by association [9]. In a country affected by an armed conflict and where some segments of the population still lack access to basic infrastructure, food and education, our results show that the food industry is often described as contributing to the prosperity of the country, at least in the short term. This means that investments and employment, made possible by corporations with the support from the government and perhaps the public, may be prioritised over public health goals. Many actors in public health nutrition advocating for the rights to food often felt unsafe in their positions, when criticising the products or actions of that industry.

The industry tried to influence the science on nutrition and diet-related issues in Colombia, through its in-house production and dissemination of science and its use of third parties such as ILSI, which in turn had direct impact on policy.

In Colombia, industry actors were directly involved in policy making in the country. The industry was also promoting self-regulation, which is a standard industry approach to avoid government regulation that has been shown to be ineffective [45–47]. Civil society organisations have monitored the voluntary commitments made by the food industry in 2016, which aimed at limiting the sales of unhealthy products in schools [A144]. They concluded that food companies did not meet their initial objectives [48]. Industry responded by launching another self-regulatory initiative in September 2019 in the presence of the Minister of Health [49].

We found no evidence of the use of legal strategies in Colombia in our document analysis but some examples of litigation were described in our interviews. This is explained by the fact that we only collected documents published in 2019, while our interviewees discussed cases from 2016 and 2018.

The food industry in Colombia is using discursive strategies, where the industry presented itself as an essential economic actor in the country and framed the debate on NCD and other diet-related issues.

This was the first study of the CPA of the food industry in Latin America. Our results are consistent with findings from other studies on the CPA of the food industry globally, where all CPA strategies are used by large economic actors to influence policy, research and practice [16, 23, 28, 50]. However, in Colombia, the proximity between the industry, the government and the media is particularly evident and remains largely unquestioned. The influence of vulnerable populations in communities, including in areas that lack support from the government, and the threats on civil society organisations are also striking and worrisome. CPA practices of the food industry could facilitate the distribution of ultra-processed products, with for example the assumption that such products can help addressing hunger, as was the case with fortified beverages distributed in the far north of the country to a vulnerable segment of the population. Such hunger alleviation initiatives have also been used in other parts of the globe [51]. Therefore, these CPA practices may pose a risk to population health and that of children in particular, since the consumption of ultra-processed foods has been associated with the development of NCDs [52, 53], but also to the protection of fundamental human rights to health and adequate food, as recognized by the United Nations Special Rapporteur on the right to health [54].

This study also builds upon the growing literature of commercial determinants of health, which focuses on how corporations market and lobby for products that harmful to health [19, 55]. By applying a CPA analysis in Colombia, this study further identifies and exposes the practices of corporations, which could help assist academics, advocates and government officials counter industry interference and help prepare, enact and implement evidence-based public health policies [56]. Given that commercial determinants views the industry as the vector of disease [55], future research should explore cross-industry and cross-policy comparisons to identify evolving patterns and trends in industry activity and policy interference.

This study has some limitations. For our interviews, we experienced better access to civil society actors, compared to actors in the food industry and the government, universities and professional associations. We also found limited information available to the public regarding the interactions of these individuals with the food industry. This might be due to the fact that these interactions are not known to the public, but rather happen in private space, like personal meetings and through emails or phone calls, and that these individuals are not necessarily willing to critically discuss these interactions. In addition, we limited our searches of publicly available information to data published in the past few months and a limited number of food industry actors, due to time constraints. Future studies could cover a longer period of time and include additional actors.

Finally, there are solutions to address and prevent negative influence from the food industry on public health policy, research and practice in Colombia and abroad, as recently detailed in a scoping review [56]. We noted the existence and availability, online, of a register of lobbyists in the country, but it has not been updated since 2014. There is also a law that prohibits members of the governments from working in a sector that they used to regulate, but participants explained that the law is not necessarily implemented (Article 3 from the Law 1474 of 2011). In Colombia, the ‘Colectivo de Abogados José Alvear Restrepo’ (The ‘José Alvear Restrepo’s Lawyers Collective’, CAJAR), launched a ‘Pact for transparency in public health policies and against interference with [human] rights’ [57]. The Pact proposed a number of actions that could help in reducing the interference of the food industry in the country (Additional file 7) [57]. Furthermore, the protection of public health, beyond policies, from undue influence by corporations needs to be addressed in Colombia.

## Conclusions

In conclusion, the food industry has penetrated many institutions and closely interacts with individuals in policy, communities, research and the media in Colombia. It is crucial that these actors understand the risks associated with the CPA and the commercial determinants of heath, and that solutions are developed and implemented to address the influence from the vested, profits driven interests of the food industry.

## Supplementary information

**Additional file 1.** Conceptual framework for categorising the corporate political activity of the food industry.

**Additional file 2.** Consolidated criteria for reporting qualitative research (COREQ) checklist.

**Additional file 3.** Sources.docx: Sources of information to identify the corporate political of the food industry in Colombia.

**Additional file 4.** Data collected from publicly available information.

**Additional file 5.** Interview guide (Spanish).

**Additional file 6.** Spanish version of the article.

**Additional file 7.** Actions proposed in the “Pact for transparency in public health policies and against interference with [human] rights”, adapted from the Colectivo de Abogados José Alvear Restrepo.

## Data Availability

All data from the public domain collected during this study are available with this manuscript as Additional file 4. Data from our interviews are available from the corresponding author on reasonable request.
