# Supplementary material for: “The architecture of the state was transformed in favour of the interests of companies”: corporate political activity of the food industry in Colombia
Source: Global Health. 2020 Oct 12;16:97. doi: 10.1186/s12992-020-00631-x (PMC7552360; doi:10.1186/s12992-020-00631-x)
Supplement: Supplementary file 1 — Additional file 1. Conceptual framework for categorising the corporate political activity of the food industry. [file 12992_2020_631_MOESM1_ESM.docx]

Additional file 1: Conceptual framework for categorising the corporate political activity of the food industry (1)

| Instrumental Strategies | Practices | Mechanisms |
| --- | --- | --- |
| Coalition management | Constituency recruitment – external - Establish relationships with key opinion leaders and health organisations | Promote public-private interactions with health organisations |
|  |  | Support professional organisations, including through funding and / or advertising in their publications |
|  |  | Establish informal relationships with key opinion leaders |
|  |  | Support the placement of industry-friendly personnel within health organisations |
|  | Constituency recruitment – external - Seek involvement in the community | Undertake corporate philanthropy |
|  |  | Support physical activity initiatives |
|  |  | Support events (such as for youth or the arts) and community-level initiatives |
|  | Constituency recruitment – external - Establish relationships with the media | Establish close relationships with media organisations, journalists and bloggers to facilitate media advocacy |
|  | Constituency fabrication | Establish fake grassroots organisations (‘astroturfing’) |
|  |  | Procure the support of community and business groups to oppose public health measures |
|  | Constituency fragmentation and destabilisation | Discrediting public health advocates personally and publicly, e.g. through the media, blogs |
|  |  | Infiltrate, monitor the operation and advocacy strategies of public health advocates, groups and organisations |
|  |  | Creating antagonism between professionals |
| Information management | Production | Fund research, including through academics, ghost writers, own research institutions and front groups |
|  | Amplification | Cherry pick data that favours the industry, including use of non-peer reviewed or unpublished evidence |
|  |  | Participate in and host scientific events |
|  |  | Propose industry-sponsored education |
|  | Suppression | Suppress the dissemination of research that does not fit the industry’s interests |
|  |  | Emphasise disagreement among scientists and focus on doubt in science |
|  |  | Criticise evidence, and emphasise its complexity and uncertainty |
|  | Credibility | Fronting: concealing industry links to information/evidence, including through the use of scientists as advisers, consultants or spokespersons |
| Direct involvement and influence in policy | Indirect access | Lobby directly and indirectly (through third parties) to influence legislation and regulation so that it is favourable to the industry |
|  |  | Use the “revolving door”, i.e. ex-food industry staff work in government organisations and vice versa |
|  | Incentives | Fund and provide financial incentives to political parties and policy makers (donations, gifts, entertainment or other financial inducements) |
|  | Threats | Threaten to withdraw investments if new public health policies are introduced |
|  | Actor in government decision making | Seek involvement in working groups, technical groups and advisory groups |
|  |  | Provide technical support and advice to policy-makers (including consultation) |
| Legal actions | Use legal action (or the threat thereof) against public policies or opponents | Litigate or threaten to litigate against governments, organisations or individuals |
|  | Influence the development of trade and investment agreements | Influence the development of trade and investment agreements such that clauses favourable to the industry are included (e.g., limited trade restrictions, mechanisms for corporations to sue governments) |

| Discursive Strategies | Domain | Argument |
| --- | --- | --- |
|  | The economy | Stress the number of jobs supported and the money generated for the economy |
|  | Governance | Demonise the ‘nanny state’ |
|  | Expected food industry costs | Policy will lead to reduced sales/jobs |
|  |  | Cost of compliance will be high |
|  | Frame the debate on diet- and public health-related issues | Stress the good traits of the food industry |
|  |  | Shift the blame away from the food industry and its products, e.g. focus on individual responsibility, role of parents, physical inactivity |
|  |  | Promote industry´s preferred solutions: education, balanced diets, information, public private initiatives, self-regulation (reformulation) |

1. Mialon M, Julia C, Hercberg S. The policy dystopia model adapted to the food industry: the example of the Nutri-Score saga in France. World Nutrition. 2018;9(2):109–120.
