# Supplementary material for: “The architecture of the state was transformed in favour of the interests of companies”: corporate political activity of the food industry in Colombia
Source: Global Health. 2020 Oct 12;16:97. doi: 10.1186/s12992-020-00631-x (PMC7552360; doi:10.1186/s12992-020-00631-x)
Supplement: Supplementary file 2 — Additional file 2. Consolidated criteria for reporting qualitative research (COREQ) checklist. [file 12992_2020_631_MOESM2_ESM.docx]

Additional file 2: Consolidated criteria for reporting qualitative research (COREQ) checklist (1)

| Item number | Item | Guide questions/description | Answers for the research study |
| --- | --- | --- | --- |
| Domain 1: Research team and reflexivity | | | |
| Personal Characteristics | | | |
| 1 | Interviewer | Which author/s conducted the interview or focus group? | First author |
| 2 | Credentials | What were the researcher’s credentials? E.g. PhD, MD | PhD, MSc, BSc |
| 3 | Occupation | What was their occupation at the time of the study? | Research fellow |
| 4 | Gender | Was the researcher male or female? | Female |
| 5 | Experience and training | What experience or training did the researcher have? | Experience conducting qualitative research |
| Relationship with participants | | | |
| 6 | Relationship established | Was a relationship established prior to study commencement? | I knew, professionally, some participants. |
| 7 | Participant knowledge of the interviewer | What did the participants know about the researcher? e.g. personal goals, reasons for doing the research | Some knew my research |
| 8 | Interviewer characteristics | What characteristics were reported about the interviewer/facilitator? e.g. Bias, assumptions, reasons and interests in the research topic | Interest in public health and the influence of the food industry – see interview guide as well |
| Domain 2: study design | | | |
| Theoretical framework | | | |
| 9 | Methodological orientation and Theory | What methodological orientation was stated to underpin the study? e.g. grounded theory, discourse analysis, ethnography, phenomenology, content analysis | Thematic analysis using a conceptual framework – critical social science theory |
| Participant selection | | | |
| 10 | Sampling | How were participants selected? e.g. purposive, convenience, consecutive, snowball | Purposive, snowball |
| 11 | Method of approach | How were participants approached? e.g. face-to-face, telephone, mail, email | Emails and direct contact of colleagues |
| 12 | Sample size | How many participants were in the study? | 18 |
| 13 | Non-participation | How many people refused to participate or dropped out? Reasons? | None |
| Setting | | | |
| 14 | Setting of data collection | Where was the data collected? e.g. home, clinic, workplace | At a location chosen by the participants |
| 15 | Presence of non-participants | Was anyone else present besides the participants and researchers? | No, except from two interviews where a research assistant was present |
| 16 | Description of sample | What are the important characteristics of the sample? e.g. demographic data, date | All ages, different genders, academics, journalists, policy makers, etc. |
| Data collection | | | |
| 17 | Interview guide | Were questions, prompts, guides provided by the authors? Was it pilot tested? | Questioned not shared with participants. The guide was pilot tested in 2015. |
| 18 | Repeat interviews | Were repeat interviews carried out? If yes, how many? | Yes, for one interviewee as we could not cover everything the first time (participant was late) |
| 19 | Audio/visual recording | Did the research use audio or visual recording to collect the data? | Digital audio recording |
| 20 | Field notes | Were field notes made during and/or after the interview or focus group? | Yes, after each interview |
| 21 | Duration | What was the duration of the interviews or focus group? | 60min on average |
| 22 | Data saturation | Was data saturation discussed? | Yes |
| 23 | Transcripts returned | Were transcripts returned to participants for comment and/or correction? | Yes, if asked |
| Domain 3: analysis and findings | | | |
| Data analysis | | | |
| 24 | Number of data coders | How many data coders coded the data? | One, and reviewed by two researchers (10 and 100% of the data) |
| 25 | Description of the coding tree | Did authors provide a description of the coding tree? | Yes |
| 26 | Derivation of themes | Were themes identified in advance or derived from the data? | In advance and modified accordingly to the findings (iterative) |
| 27 | Software | What software, if applicable, was used to manage the data? | Not applicable |
| 28 | Participant checking | Did participants provide feedback on the findings? | Yes |
| Reporting | | | |
| 29 | Quotations presented | Were participant quotations presented to illustrate the themes / findings? Was each quotation identified? e.g. participant number | Yes, identified with a generic term for the profession |
| 30 | Data and findings consistent | Was there consistency between the data presented and the findings? | Yes |
| 31 | Clarity of major themes | Were major themes clearly presented in the findings? | Yes |
| 32 | Clarity of minor themes | Is there a description of diverse cases or discussion of minor themes? | Yes |

1. Tong A, Sainsbury P, Craig J. Consolidated criteria for reporting qualitative research (COREQ): a 32-item checklist for interviews and focus groups. Int J Qual Health Care. 2007 Dec;19(6):349–357.
