# Supplementary material for: “The architecture of the state was transformed in favour of the interests of companies”: corporate political activity of the food industry in Colombia
Source: Global Health. 2020 Oct 12;16:97. doi: 10.1186/s12992-020-00631-x (PMC7552360; doi:10.1186/s12992-020-00631-x)
Supplement: Supplementary file 3 — Additional file 3. Sources.docx: Sources of information to identify the corporate political of the food industry in Colombia. [file 12992_2020_631_MOESM3_ESM.docx]

Additional file 3: Sources of information to identify the corporate political of the food industry in Colombia

| **Nature of the source of information** | **Source of information** | **Country-specific URL** |
| --- | --- | --- |
|  | Country-specific website(s) of the industry actor, including CSR webpages or report or website, webpages or information in annual reports of a company's philanthropic activities (or the company’s country-specific activities on its international website) | o Coca Cola: https://www.coca-cola.com.co/es/co/home/  https://www.coca-colafemsa.com/presencia/presencia-colombia.html  o Danone/Alqueria:  https://www.mundoalqueria.co/  https://www.alqueria.com.co/  http://www.alqueriaportubienestar.com/  o Ferrero: no national website o General Mills: no national website o Grupo Bimbo: no national website o Kellogg’s: https://www.kelloggs.com.co/es_CO/home.html  o Mars: no national website o McDonald’s: https://www.mcdonalds.com.co/  o Mondelez: https://www.mondelezinternational.com/colombia  o Nestlé:  https://www.corporativa.nestle.com.co/  https://www.nestle-contigo.co/  https://www.unidosporninossaludables.com.co/  o PepsiCo: http://www.pepsico.com.co/  o Unilever: no national website  ° Grupo Nutresa S. A.:  https://www.gruponutresa.com/  http://fundacionnutresa.com/  http://www.vidarium.org/  ° Postobón:  https://www.postobon.com/  https://tomatelavida.com.co/  ° Colanta: https://colanta.com/corporativo.html  ° Alpina:  https://www.alpina.com/  https://www.fundacionalpina.org/es-es/   ° Grupo Exito:  https://www.grupoexito.com.co/es/  https://www.fundacionexito.org/   o Asociación Colombiana de Ciencia y Tecnología de Alimentos (ACTA): https://portal.acta.org.co/  o Asociación Nacional de Empresarios de Colombia (ANDI): http://www.andi.com.co/Home/Camara/16-industria-de-alimentos  http://www.andi.com.co/Home/Camara/19-industria-de-bebidas http://www.vamoscolombia.org/fundacion-andi/   ° ILSI NOR-ANDINO: https://ilsinorandino.org/   ° Decido lo que como: https://decidoloquecomo.com/ ° Bebidas de tu lado: http://bebidasdetulado.com/ |
| Government material: Ministries (and related agencies) responsible for diet- related issues | Websites of Ministry in charge of health and related agencies (National level), Ministry of Education | Ministerio de Salud y Protección Social: - https://www.minsalud.gov.co/Paginas/default.aspx   Transparencia y acceso a la información pública: https://www.minsalud.gov.co/atencion/Paginas/transparencia-acceso-informacion.aspx   INSTITUTO NACIONAL DE SALUD: http://www.ins.gov.co/Paginas/inicio.aspx   Ministerio de Educacion: https://www.mineducacion.gov.co/1759/w3-channel.html?_noredirect=1   Instituto Colombiano de Bienestar Familiar: https://www.icbf.gov.co/ |
|  |  |  |
|  |  |  |
|  | Websites of the Parliament (National level) and Presidency | Congresso de la Republica de Colombia: - Senado de la Republica: http://www.senado.gov.co/  -> transparencia: http://www.senado.gov.co/transparencia/datos-abiertos   - Camara de Represententes: http://www.camara.gov.co/  -> transparencia: http://www.camara.gov.co/transparencia-y-acceso-a-la-informacion-publica   Presidency of the Republic: https://id.presidencia.gov.co/gobierno/ |
|  |  |  |
|  |  |  |
|  | Register of lobbyists (National level) | Register for the period 2014-2018:http://www.camara.gov.co/participacion-ciudadana/registro-publico-de-cabilderos |
|  | Other | Congreso Visible: http://www.congresovisible.org/ |
|  | Websites of major political parties and websites of commissions in charge of elections (National and State Level) | Consejo Nacional Electoral: https://www.cne.gov.co/tramites-y-servicios/cuentas-claras  http://www.cnecuentasclaras.gov.co/ - will be the subject of a specific study  All parties done, no relevant information  Partido Social de Unidad Nacional: www.partidodelau.com   Partido Conservador Colombiano: partidoconservador.com   Partido Liberal Colombiano: www.partidoliberal.org.co   Centro Democrático: www.centrodemocratico.com |
| Other material | Websites of major universities with a School/Department of nutrition/dietetics and/or exercise/physical activity | Universidad de Antioquia - Escuela de Nutrición y Dietética: http://www.udea.edu.co/wps/portal/udea/web/inicio/unidades-academicas/nutricion-dietetica   Pontificia Universidad Javeriana - Faculdad de Ciensa - Departamento de Nutrición y Bioquímica: https://ciencias.javeriana.edu.co/departamentos-instituto/nutricion-bioquimica  Universidad Nacional de Colombia - Facultad de Medicinca-Departamento de Nutrición: http://medicina.bogota.unal.edu.co/dependencias/departamentos/nutricion-humana#ejes_misionales-2   Foundation for Research in Nutrition and Health, FINUSAD: https://www.finusad.com/ |
|  |  |  |
|  | Websites of major conferences in dietetics/nutrition (National level) | XVIII Congreso Colombiano de nutricion y dietética y II Internacional en alimentacion y nutricion: https://acodin.org/congreso-2019/  33° Congreso de Nutrición Clínica y Metabolismo y 4° Congreso FELANPE Regional Andino: https://www.nutriclinicacolombia.org/congreso/   Congreso salud publica Colombia - II Congreso Bienal: https://www.saludpublicacolombia.org/ii-congreso-bienal/ |
|  |  |  |
|  |  |  |
|  |  |  |
|  | Websites of major professional bodies in dietetics/nutrition (National level) | ACODIN - Asociación Colombiana de Dietistas y Nutricionistas: https://acodin.org/   Asociación Colombiana de Nutricion Clinica: http://www.nutriclinicacolombia.org/   COLNUD - Colegio colombiano de Nutricionistas Dietistas: http://www.colnud.co/   Asociaciòn Colombiana de Salud Pública: https://www.saludpublicacolombia.org/   ACOCIB - Asociación Colombiana de Obesidad y Cirugía Bariátrica: https://www.acocib.com/ |
|  |  |  |
|  |  |  |
| Industry own material and government | News and media releases | **Google News** with name of the companies included in our sample  **Newspapers: El Tiempo:** www.eltiempo.com - not freely accessible **El Espectador:** elespectador.com - not freely accessible |
| Industry own material | Twitter account (national only) | Coca Cola: https://twitter.com/cocacolacol?lang=en  Danone Alqueria: https://twitter.com/alqueriaoficial?lang=es  Ferrero: excluded as it was only a commercial account to market products Mc Donald's: https://twitter.com/mcdonaldscol?lang=en  Nestlé: https://twitter.com/nestlecolombia?lang=en  https://twitter.com/UnidosxNinosCol  PepsiCo: https://twitter.com/pepsicolombia?lang=en   Alpina: https://twitter.com/alpina  Colanta: https://twitter.com/ColantaOficial  Postobon: https://twitter.com/postobonoficial  Nutresa:  https://twitter.com/fnutresa?lang=es  https://twitter.com/grupo_nutresa?lang=es   Grupo Exito: https://twitter.com/Fundacion_Exito  https://twitter.com/Grupo_Exito?ref_src=twsrc%5Etfw    ACTA: https://twitter.com/acta_col/  ANDI: https://twitter.com/ANDI_Colombia only data for after 16 May 2019 https://twitter.com/FundacionANDI  ILSI NOR-ANDINO: https://twitter.com/ILSINorAndino   https://twitter.com/decidoloquecomo - this page started on 3 March 2019 https://twitter.com/bebidasdetulado - no activity since Aug 2018, started again on 15 April 2019 |
