# Supplementary material for: “The architecture of the state was transformed in favour of the interests of companies”: corporate political activity of the food industry in Colombia
Source: Global Health. 2020 Oct 12;16:97. doi: 10.1186/s12992-020-00631-x (PMC7552360; doi:10.1186/s12992-020-00631-x)
Supplement: Supplementary file 4 — Additional file 4. Data collected from publicly available information. [file 12992_2020_631_MOESM4_ESM.docx]

| Code | Food industry actor | Source | Strategy | Code | Data coded | Notes | Website URL | Date collected |
| --- | --- | --- | --- | --- | --- | --- | --- | --- |
| A1 | ACTA | Industry website | Coalition management | Health organisations and government bodies Internal | Representación Institucional La trayectoria de ACTA y el potencial que tiene de ofrecer un punto de vista independiente, técnico y con conocimiento del medio, le ha permitido ser considerada como asesora y consejera de diferentes instituciones. Es así como actualmente (...) trabaja en alianza con la Asociación Colombiana de Facultades de Nutrición – ACOFANUD y con la Asociación de Exalumnos de la Universidad de Los Andes – UNIANDINOS, igualmente desarrolla permanentemente actividades con la Cámara de Alimentos de la ANDI y la Cámara Procultivos de la misma entidad. También Apoya a la Cámara de Comercio de Bogotá en el área de Agroindustria. A nivel internacional, ACTA pertenece a la Asociación Latinoamericana y del Caribe de Ciencia y Tecnología de Alimentos –ALACCTA-, al International Union of Food Science and Technology –IUFoST- , al Institute of Food Technologists –IFT- a la International Association for Food Protection- IAFP y tiene un convenio de cooperación con la Universidad Politécnica de Valencia– UPV. |  | <https://portal.acta.org.co/representacion-institucional/> | 13-Aug-19 |
| A2 | ACTA | Industry website | Direct involvement and influence in policy Discursive strategy | Direct actors/Indirect access Frame the debate | Ministerio de salud y protección social. Tradicionalmente ACTA ha mantenido una relación continua con el ministerio de salud y protección social de Colombia y apoya difundiendo las diversas normas que se encuentran en consulta pública para su fortalecimiento, actualización y mejoramiento continuo, en la actualidad ACTA por invitación del Ministerio convoca a la industria y expertos del país dando continuidad al trabajo de “Prevención de las enfermedades cardiovasculares en las Américas, mediante la reducción de la ingesta de sal alimentaria en toda la población; el grupo de trabajo de la Estrategia Nacional de Reducción del Consumo de Sal, en el frente de la Industria ha dado inicio a la segunda etapa de trabajo; para ello ha priorizado otro grupo de alimentos de alto contenido de sodio.  Los nuevos grupos de alimentos priorizados son: salsas comerciales, sopas, bases, caldos granulados y en cubo, embutidos de pollo, cereales extruidos de desayuno, atún, sardinas, grasas, aderezos y salsas comerciales. |  | <https://portal.acta.org.co/representacion-institucional/> | 13-Aug-19 |
| A3 | ACTA | Industry website | Information management | Suppression | Es nuestro deber llamar la atención al uso indebido del termino “ultraprocesado”, el cual empezó a imponerse desde la clasificación NOVA. Desde la ciencia de los alimentos, la clasificación NOVA NO TIENE una base cientifica coherente. Para el IFT[1] y ALACCTA esta clasificación genera confusión y principalmente desinformación. (...) Ante estas evidencias, es importante hacer claridad que la sociedades cientificas en Ciencia y Tecnología de Alimentos, como el IFT, IUFoSt, el Codex Alimentario; buscan generar y transferir nuevo conocimiento; que algunas generalizaciones como que los cientificos estan siendo patrocinados por la industria, sin la evidencia pertinente, puede llegar a convertirse en injuria. (....) La innovación en nuestra propuesta es incluir en el GDA, EL NÚMERO DE PORCIONES POR ENVASE y regular este número de acuerdo con las GABAS – Guías Alimentarias Basadas en Alimentos para la población colombiana; y no dejarlo al criterio del industrial; y que tanto el etiquetado GDA y el número de porciones sea un parámetro principal para la obtención y renovación de los registros, permisos y notificaciones sanitarias otorgadas por el INVIMA. | See the webpage for more details | <https://portal.acta.org.co/ponencia-acta-etiquetado-nutricional/> | 13-Aug-19 |
| A4 | Alpina | Industry website | Information management | Amplification | Componentes de la leche esenciales en el funcionamiento del organismo  22 Ene, 2019 La leche uno de los alimentos más consumidos en el mundo debido a su alto aporte nutricional, posee componentes que son esenciales en todas las etapas de la vida del ser humano, así como en periodos de embarazo y lactancia. Según la Organización de las Naciones Unidas para la Alimentación y la Agricultura FAO, en el mundo, seis mil millones de personas consumen leche. En Colombia, la cifra es de 140 litros al año por persona, de acuerdo con la Asociación Colombiana de Procesadores de Leche, Asoleche. La meta es que en cinco años aumente la preferencia por este alimento y sus derivados alcanzando 170 litros por persona. [Then see details on website] | See here for more examples: https://www.alpina.com/mundo-alpina/nutricion | <https://www.alpina.com/mundo-alpina/nutricion/componentes-de-la-leche> | 19-Aug-19 |
| A5 | Alpina | Industry website | Information management | Amplification | Las grasas de la leche y sus beneficios Debido a su importancia, las investigaciones sobre la leche y sus derivados son constantes. La ciencia dice lo siguiente con respecto a los efectos de las grasas sobre el organismo: El consumo de leche entera no presenta riesgos de padecimiento de enfermedades cardiovasculares en personas sanas. Por el contrario, se ha demostrado que consumir leche mejora la salud cardiovascular. La ingesta de productos lácteos no produce una elevación de los niveles de colesterol en sangre. Sin lugar a dudas, es sorprendente el valor de este alimento. Pero hay más para decir, y es lo que vamos a hacer a continuación. | See more details on the webpage | <https://www.alpina.com/mundo-alpina/nutricion/grasas-de-leche-importante-para-el-organismo> | 19-Aug-19 |
| A6 | Alpina | Industry website | Information management | Amplification | En este momento nos encontramos en la consolidación de la estrategia de “Con-ciencia Láctea”, que busca promover el consumo de lácteos en grupos importantes para el país como son el gremio médico, la academia, entidades gubernamentales y no gubernamentales, entre otros. Está claro y de lácteos tiene una relación positiva con el crecimiento y desarrollo de las personas en diferentes momentos de la vida, además de proveer casi de manera exclusiva proteínas y nutrientes como el calcio, vitaminas A y D, minerales, fósforo y complejo B, que son esenciales para lograr una alimentación completa y balanceada. Adicionalmente, importantes componentes lácteos pueden cionales sino funcionales, relacionados con salud y bienestar Continuamos consolidando nuestra estrategia de relacionamiento con el gremio médico para fortalecer la categoría Alpina Baby, entiendo los.....consumidores | Info also on the Foundation - see details collected from the Foundation webpage for up-to-date info | <https://www.alpina.com/Portals/_default/Sostenibilidad/Informes-sostenibilidad/Informe-de-Sostenibilidad-2018.pdf> | 19-Aug-19 |
| A7 | ANDI | Twitter | Coalition management | Community | ANDI Retweeted Jorge Jesús Montaño Acosta ‏@jormon26  Jul 5 Este sábado 8 am con liderazgo @ANDI_Colombia con @AVives y empresas afiliadas como @puertobq @zfbaq @ElectricaribeSA apuestan a festival fútbol con 120 niños en el Moderno de los barrios Rebolo, La Luz, Ferry y Villanueva. @CardenalNoticia @CUARTOPODERHF |  | <https://twitter.com/jormon26/status/1147105023282819074> | 29-Jul-19 |
| A8 | ANDI | Twitter | Coalition management | Internal | ANDI a retweeté  Camilo Montes Pineda @CamiloMontes  30 mai Comienza en Santiago de Chile el Encuento #ALAIAB donde debatiremos asuntos como la lucha contra la obesidad, directrices del @FAOWHOCodex, desarrollo sostenible, entre otros Tengo el honor de ejercer la secretaría de la junta directiva  Camilo Montes Pineda @CamiloMontes  31 mai Buena jornada de trabajo en encuentro #ALAIAB en Santiago de Chile Fuerte compromiso con la promoción de hábitos de vida saludable en y con avanzar en economía circular! |  | <https://twitter.com/CamiloMontes/status/1134088499156660225> | 30-Jul-19 |
| A9 | ANDI | Twitter | Coalition management | Community | Fundación ANDI a retweeté  Ejército Nacional de Colombia Compte certifié @COL_EJERCITO  2 févr. Desde #VillaDelRosario voluntarios de @FundacionANDI, soldados de la #BrigadaDeAcciónIntegral1 , @Ejercito_CAAID, #FeEnColombia, #Brigada30 y @Ejercito_Div2 trabajan en la Escuela María Inmaculada, para crear ambientes escolares agradables. | Many more Tweet about this and another event in the North later during the year | <https://twitter.com/COL_EJERCITO/status/1091762418391400449> | 30-Jul-19 |
| A10 | ANDI | Twitter | Coalition management | Community | Fundación ANDI @FundacionANDI  28 janv. En dos años, 2.700 voluntarios de 149 empresas han participado de los 13 encuentros de #VamosColombia que han beneficiado a más de 7.000 personas. Este año comenzamos en Villa del Rosario, Norte de Santander. #VamosFronteras https://bit.ly/2FMdtsK |  | <https://twitter.com/FundacionANDI/status/1089907799654584321> | 30-Jul-19 |
| A11 | ANDI | Twitter | Coalition management | Community | Fundación ANDI @FundacionANDI  17 janv. El voluntariado corporativo es una herramienta poderosa para construir confianza y reconciliación como país, inspirando al sector privado a trabajar hombro a hombro con comunidades vulnerables. Nuestra propuesta es #VamosColombia. Conócela y súmate. https://bit.ly/2i7jpl6 | see also: https://twitter.com/FundacionANDI/status/1084805810700648449 | <https://twitter.com/FundacionANDI/status/1085912914131705856> | 30-Jul-19 |
| A12 | ANDI | Industry website | Coalition management | Internal | Bebidas de tu lado ¿QUIÉNES SOMOS? “Bebidas de tu lado” es una iniciativa de la Cámara de la Industria de Bebidas de la ANDI que tras un año de la suscripción de 5 compromisos de autorregulación quiere contarle a los colombianos sus avances y logros. |  | <http://bebidasdetulado.com/quienes-somos/> | 14-Aug-19 |
| A13 | ANDI | Twitter | Coalition management Discursive strategy | Community Frame the debate | ANDI a retweeté Felipe Torres @l_felipetorres  4 juil. Empresas de #ANDIBebidas pioneras en programa de reciclaje en Santa Marta ♻️. Claro compromiso del sector con el desarrollo sostenible y el caribe. #MovimientoRE #economiacircular @ANDI_Colombia |  | https://twitter.com/l_felipetorres/status/1146792672658677760 | 30-Jul-19 |
| A14 | ANDI | Government website | Direct involvement and influence in policy | Actor in government decision making | REPORT OF THE FORTY-FIFTH SESSION OF THE CODEX COMMITTEE ON FOOD LABELLING Ottawa, Ontario, Canada -13 - 17 May 2019 - List of delegates Codex for Colombia: Dr Juan Camilo Montes Director de la Cámara de la Industria de Alimentos - ANDI Calle 73 No. 8 – 13 piso 6 Bogotá D.C Colombia (...)  Ms Laura Otalora Pharmacist ANDI Carrera 83 N°71-81 piso 3 Bogotá Colombia (....)  Mrs Diana Carolina Rojas Gonzalez Representante ANDI ANDI Dirección: Carrera 55ª NO. 134ª – 85. Colombia (...) |  | <http://www.fao.org/fao-who-codexalimentarius/sh-proxy/en/?lnk=1&url=https%253A%252F%252Fworkspace.fao.org%252Fsites%252Fcodex%252FMeetings%252FCX-714-45%252FFinal%252520Report%252FREP19_FLe.pdf> | 20-Aug-19 |
| A15 | ANDI | Twitter | Direct involvement and influence in policy | Actor in government decision making | ANDI a retweeté Felipe Torres @l_felipetorres  25 juin La Cámara de la Industria de Bebidas de la ANDI apoya el debate técnico sobre el etiquetado que se debe adoptar en Colombia, este debate debe incluir al Gobierno, la academia, la sociedad civil, las agremiaciones y el sector privado. #EmpresariosEtiqueten @ANDI_Colombia |  | <https://twitter.com/l_felipetorres/status/1143501479757721600> | 30-Jul-19 |
| A16 | ANDI | Twitter | Direct involvement and influence in policy | Indirect access | Camilo Montes Pineda @CamiloMontes  12 juin En junta #ANDIAlimentos tuvimos conversatorio con Rep @oscardarioperez hablamos: + Metas y oportunidades del Plan Nacional de Dllo + Avances en Empleo, PIB, Exportaciones del sector + Herramientas de la ley de financiación  + Desarrollo Agroindustrial Gracias por su presencia! |  | <https://twitter.com/CamiloMontes/status/1138875209514196992> | 30-Jul-19 |
| A17 | ANDI | Twitter | Direct involvement and influence in policy | Indirect access | ANDI a retweeté Presidencia Colombia Compte certifié @infopresidencia  21 mai El Presidente @IvanDuque clausurará en la tarde de este martes la 75° Asamblea Seccional de la @ANDI_Colombia Bogotá-Cundinamarca-Boyacá, con un diálogo que sostendrá con los empresarios de esta región del país.   >>> https://bit.ly/2Qg45A4 | On Twitter and webpage of the President: https://id.presidencia.gov.co/Paginas/prensa/2019/190520-Este-martes-el-Presidente-clausura-la-75-Asamblea-Seccional-de-la-ANDI.aspx | <https://twitter.com/infopresidencia/status/1130662697450835968> | 30-Jul-19 |
| A18 | ANDI | Media | Direct involvement and influence in policy Discursive strategy | Actor in government decision making | Es necesario avanzar en un etiquetado frontal informativo para los alimentos Durante las últimas semanas hemos participado en dos audiencias públicas sobre dos proyectos de ley que buscan regular el etiquetado frontal de los productos alimenticios, lo cual refleja la alta importancia que tiene este tema para los colombianos. Un análisis por Camilo Montes, director de la Cámara de la Industria de Alimentos de la Andi. Si bien desde la industria aplaudimos los esfuerzos que ha hecho el Congreso de la República para poner este tema en la agenda pública, es claro que el país no puede avanzar solo en el frente regulatorio, sino que esta debe ser una tarea multisectorial para afrontar desafíos de salud pública como lo son la obesidad, la diabetes y otras enfermedades crónicas no transmisibles. Es por esto que desde la industria hemos implementado de forma voluntaria, a partir de 2016, un etiquetado frontal en más de 4.771 referencias alimenticias, para que los colombianos puedan comenzar a tomar decisiones sobre su alimentación sin necesidad de alarmismo. Además, esto es solo el primer paso, porque no podemos olvidar que la obesidad es multifactorial y se deben afrontar otros problemas como la inactividad física. |  | <https://www.dinero.com/pais/articulo/es-necesario-avanzar-en-un-etiquetado-frontal-informativo-para-los-alimentos-por-camilo-montes/272464> | 22-Aug-19 |
| A19 | ANDI | Industry website | Discursive strategy | Frame the debate | Promovemos entornos saludables: como complemento a las actividades individuales de las empresas afiliadas, desarrollamos acciones colectivas de posicionamiento de la industria, liderando campañas de responsabilidad social para la promoción de hábitos de vida saludables y educación de los consumidores en los asuntos relacionados con la alimentación, especialmente entre la población infantil. |  | <http://www.andi.com.co/Home/Camara/16-industria-de-alimentos> | 29-Jul-19 |
| A20 | ANDI | Industry website | Discursive strategy | Economy | Posicionar al sector de alimentos como motor de desarrollo económico y social del país a través de su liderazgo, competitividad y sostenibilidad y bajo el enfoque de cadena de valor. Para lograrlo, nuestras actividades se orientan a impulsar el desarrollo y el crecimiento de la industria de los alimentos, generando espacios de participación y promoviendo la adopción de políticas públicas que contribuyan con los objetivos legítimos del Estado de forma competitiva, proporcional y razonable. |  | <http://www.andi.com.co/Home/Camara/16-industria-de-alimentos> | 29-Jul-19 |
| A21 | ANDI | Industry website | Discursive strategy | Frame the debate | Promovemos la autorregulación: dentro del complejo y multifactorial universo determinante del desarrollo de las enfermedades crónicas no transmisibles, trabajamos en los frentes de mayor impacto dentro de nuestras industrias. Ese trabajo se refleja no en compromisos sino en acciones concretas adelantadas, conjunta e individualmente, por las empresas del sector. |  | <http://www.andi.com.co/Home/Camara/16-industria-de-alimentos> | 29-Jul-19 |
| A22 | ANDI | Twitter | Discursive strategy | Coalition management Frame the debate | ANDI a retweeté Bruce Mac Master Compte certifié @BruceMacMaster  29 juil. Las Empresas en Colombia han tenido una presencia histórica en el apoyo a las actividades deportivas que hoy nos dan tantas alegrias  Estas son algunas de las que mayor presencia han tenido por años en este proceso | Bruce Mac MasterCompte certifié @BruceMacMaster Economista / trabajando por el desarrollo económico y social / Presidente de @ANDI_Colombia / miembro Consejo Superior @Uniandes | <https://twitter.com/BruceMacMaster/status/1155894118729605120> | 29-Jul-19 |
| A23 | ANDI | Twitter | Discursive strategy | Frame the debate | ANDI a retweeté Felipe Torres @l_felipetorres  24 juil. Felipe Torres a retweeté ANDI Desde #ANDIBebidas apoyamos iniciativas que promuevan la educación y los hábitos de vida saludable. El autocuidado es una de las principales herramientas para mejorar la salud de cualquier persona. #Autocuidado #HinchasDelAutocuidado @ANDI_Colombia |  | <https://twitter.com/l_felipetorres/status/1154157981069651968> | 29-Jul-19 |
| A24 | ANDI | Twitter | Discursive strategy | Frame the debate | ANDI a retweeté Camilo Montes Pineda @CamiloMontes  25 juin Estamos tan de acuerdo con que #EmpresariosEtiqueten que promovemos la implementación de un #EtiquetadoFrontalInformativo, que desde hace algunos años empezamos a implementarlo de manera voluntaria, con el fin de brindar a los consumidores información clara |  | <https://twitter.com/CamiloMontes/status/1143502260573523968> | 30-Jul-19 |
| A25 | ANDI | Twitter | Discursive strategy | Economy | Camilo Montes Pineda @CamiloMontes  12 juin La Industria de alimentos en Colombia es un motor del desarrollo económico y social: + Gran generador de empleo formal (260.000 trabajadores) + Más de 65.000 empresas (98% Mipymes) + Altamente Exportador: Más de USD900 mill a 129 países ¡Creamos bienestar económico y social! |  | <https://twitter.com/CamiloMontes/status/1138875209514196992> | 30-Jul-19 |
| A26 | ANDI | Twitter | Discursive strategy | Frame the debate | ANDI ‏   @ANDI_Colombia  27 mai Plus Es necesario avanzar en un etiquetado frontal informativo para los alimentos. Columna de @CamiloMontes Dir de Cámara #ANDIAlimentos en https://www.dinero.com/pais/articulo/es-necesario-avanzar-en-un-etiquetado-frontal-informativo-para-los-alimentos-por-camilo-montes/272464 … vía @RevistaDinero @BruceMacMaster |  | <https://twitter.com/ANDI_Colombia/status/1132993082167894017> | 30-Jul-19 |
| A27 | ANDI | Twitter | Discursive strategy | Frame the debate | Fundación ANDI @FundacionANDI  27 juin En alianza con Fundación ANDI, productores de cebolla aseguran proveeduría a multinacional Nestlé | Other examples on Twitter - support from USAID - see also website: http://www.vamoscolombia.org/fundacion-andi/ | <https://twitter.com/FundacionANDI/status/1144222595610529793> | 30-Jul-19 |
| A28 | ANDI | Twitter | Discursive strategy | Governance | Adicionalmente, es esencial que el etiquetado esté alineado con las Guías de Etiquetado Nutricional del Codex Alimentarius, establecido por la FAO y la OMS y del cual Colombia hace parte junto con 187 países, para que se le facilite al consumidor elegir su alimentación con discernimiento a partir de sus propias realidades nutricionales. |  | <https://twitter.com/FundacionANDI/status/1085912914131705856> | 30-Jul-19 |
| A29 | ANDI | Twitter | Discursive strategy | Frame the debate | Bogotá, 17 de junio de 2019. La Cámara de la Industria de Alimentos considera primordial que se dé un debate sobre el etiquetado frontal con base en información técnica y científica que respalde cualquier decisión que pueda tener un impacto en la salud pública. Además, este debe proveer información clara, veraz y suficiente, lo cual tendría que ir acompañado de educación para que los consumidores puedan interpretar las cantidades para tomar decisiones conscientes de lo que consumen. En ese sentido, es importante tener en cuenta que existen más de 24 formas diferentes de etiquetado que se están discutiendo en el mundo. “Estamos de acuerdo en la implementación y regulación del Etiquetado Frontal Informativo, que al día de hoy solo se encuentra presente en los alimentos de forma voluntaria, porque la norma nacional aún no exige de manera obligatoria su uso, como sí sucede en otros países del mundo. (...) De hecho, las empresas han implementado el etiquetado frontal desde 2016 en más de 4.771 referencias de alimentos, el cual provee información con referencia en las porciones efectivamente consumidas, respeta la autonomía y preserva el derecho de elección del consumidor. Además, presenta la cantidad y el aporte a la dieta de una porción del alimento respecto a nutrientes sensibles como grasas, azúcar y sodio. Así como la cantidad de energía (calorías). |  | <http://www.andi.com.co/Home/Noticia/12432-el-etiquetado-de-alimentos-debe-respond> | 30-Jul-19 |
| A30 | ANDI | Twitter | Discursive strategy | Frame the debate | "Tenemos que revisar entre todos los actores (industria, ONG, Congreso, academia, Gobierno, consumidores) el modelo que más se ajuste a las necesidades nutricionales de los colombianos, y esto incluye que discutamos la forma, el tamaño, el color y los íconos que le permitan al consumidor tomar decisiones informadas. Además, debería ser obligatorio”, añade Montes. (...) Porlo tanto, “reiteramos desde la Cámara de la Industria de Alimentos de la ANDI que estamos comprometidos en trabajar conjuntamente con autoridades y consumidores para lograr contar con estándares que permitan prevenir desafíos de salud pública, educar a los consumidores y seguir alimentando responsablemente a los colombianos, desde una industria que le apuesta al consumidor, al crecimiento y desarrollo empresarial, a la generación de empleo, a las exportaciones y a la competitividad del país”. |  | <http://www.andi.com.co/Home/Noticia/12432-el-etiquetado-de-alimentos-debe-respond> | 30-Jul-19 |
| A31 | ANDI | Media | Information management | Amplification Suppression | A diferencia de otras opciones de etiquetado, el GDA no discrimina, educa sobre el aporte nutricional de cada alimento y ayuda a los consumidores a tomar decisiones de consumo responsable que se ajustan a su estilo de vida y hábitos alimenticios. Desde 2016 las empresas vinculadas a la Cámara de la Industria de Bebidas de la ANDI adoptaron el GDA como esquema de autorregulación. (...) Algunos de los principales riesgos de los etiquetados de advertencia son: i) desconocen la normativa internacional, ii) no cuentan con fundamento científico y iii) en los países que se han implementado hasta el momento (caso Chile) no evidencian un impacto en la reducción de la obesidad. | See article for more details | <https://m.portafolio.co/negocios/costo-beneficio-del-etiquetado-en-los-alimentos-y-las-bebidas-531934> | 21-Aug-19 |
| A32 | ANDI | Twitter | Information management | Suppression | ANDI a retweeté Felipe Torres ‏@l_felipetorres  26 juil. Plus El debate sobre etiquetado carece de un riguroso análisis costo-beneficio según lineamientos de la @OECD. ¿Sabemos el impacto de esta medida en Mipymes que son el 95% del aparato productivo y sustento de miles de familias colombianas? @ANDI_Colombia | Felipe Torres @l_felipetorres Director Ejecutivo Cámara de la Industria de Bebidas \| Executive Director Soft Drinks Association @ANDI_Colombia MPP. LL.B. B.A. | https://twitter.com/l_felipetorres/status/1154736778173132801 | 29-Jul-19 |
| A33 | ANDI | Twitter | Information management | Amplification | ANDI a retweeté Bruce Mac Master Compte certifié @BruceMacMaster  25 juin El sector alimentos representados en la @ANDI_Colombia está de acuerdo con una reglamentación de etiquetado obligatorio. Son muchos los modelos del mundo, dentro de ellos el de la Unión Europea es considerado uno de los más modernos por la completa información que ofrece |  | <https://twitter.com/BruceMacMaster/status/1143528144839880709> | 30-Jul-19 |
| A34 | ANDI | Twitter | Information management Discursive strategy | Amplification Frame the debate | ANDI @ANDI_Colombia  25 juin La Cámara Industria #AlimentosANDI en su proceso de innovación constante, ha asumido una serie de compromisos relacionados con brindar un sistema de etiquetado que le dé al consumidor información clara y completa, para así generar un cambio positivo en los hábitos de consumo. |  | <https://twitter.com/ANDI_Colombia/status/1143518652349767681> | 30-Jul-19 |
| A35 | ANDI | Twitter | Information management Discursive strategy | Amplification Frame the debate | ANDI a retweeté Camilo Montes Pineda @CamiloMontes  19 juin Hoy están en discusión más de 24 formas diferentes de #EtiquetadoFrontal en el mundo.  Inclusive @FAOWHOCodex está trabajando el tema a nivel global! Aquí nuestra posición desde #ANDIAlimentos http://www.andi.com.co/Home/Noticia/12432-el-etiquetado-de-alimentos-debe-respond … |  | <https://twitter.com/CamiloMontes/status/1141383271642779655> | 30-Jul-19 |
| A36 | ANDI | Twitter | Information management Discursive strategy | Amplification Frame the debate | ANDI a retweeté Camilo Montes Pineda @CamiloMontes  18 juin Durante estos días, desde #ANDIAlimentos, hemos explicado a los colombianos la importancia de debatir sobre el #EtiquetadoFrontalInformativo que tenga como base información técnica y científica para respaldar cualquier decisión en la salud pública. http://www.andi.com.co/Home/Noticia/12432-el-etiquetado-de-alimentos-debe-respond … |  | <https://twitter.com/CamiloMontes/status/1140799611650662400> | 30-Jul-19 |
| A37 | ANDI | Twitter | Information management Discursive strategy | Amplification Frame the debate | Bebidas de tu lado a retweeté ANDI ‏  @ANDI_Colombia  15 mai Cámara Industria #BebidasANDI  Aprende y enseña a otros a leer el etiquetado GDA de las bebidas. Así, con #InformaciónClara, llevar una dieta balanceada será más fácil. #DeTuLado @l_felipetorres |  | <https://twitter.com/ANDI_Colombia/status/1128721710377832450> | 12-Aug-19 |
| A38 | ANDI | Industry website | Information management Discursive strategy | Amplification Frame the debate | El temido mito del sabor (...) de acuerdo con Camilo Montes, director Ejecutivo de la Cámara de la Industria de Alimentos de la ANDI, los dulces deben ser visto como ‘indulgencias’. Sin duda, lo más importante es mantener un equilibrio y un balance en la dieta para asegurar una buena salud que incluya diversidad en la alimentación de acuerdo a las necesidades y actividades de la vida diaria. |  | <https://decidoloquecomo.com/mitos-y-tendencias/el-temido-mito-del-sabor/> | 14-Aug-19 |
| A39 | ANDI | Twitter | Information management | Amplification | Para nuestro sector, un etiquetado de advertencia sataniza todas las categorías de alimentos procesados por la industria, además el etiquetado de octógonos de advertencia, solo ha sido implementado en un país, aún sin resultados positivos en salud pública”, explica Camilo Montes, Director de la Cámara de la Industria de Alimentos de la ANDI. |  | <http://www.andi.com.co/Home/Noticia/12432-el-etiquetado-de-alimentos-debe-respond> | 30-Jul-19 |
| A40 | ANDI ACTA | Media | Information management | Suppression | De acuerdo con Liliana Peralta, presidente de la Asociación de Ciencia y Tecnología de Alimentos, ACTA, la ponencia como estaba definida de los sellos de alto en azúcar y en nutrientes específicos, tiene un inconveniente y es que está basada en un sistema de clasificación de alimentos que no tiene una base científica fuerte y habla de los mal llamados alimentos procesados. Entonces, lo que podría causar es miedo en el consumidor y no va a producir cambio en la industria como pasó con Chile. “En Chile se pusieron los sellos con base en guías alimentarias y lo que ha pasado es que al final el consumidor no los deja de comprar, y el sello se volvió un paisaje y no pasa absolutamente nada porque no están diseñados basándose en qué puedan decir los contenidos, sino que se volvió más emocional”, explica la presidente de la ACTA. De igual manera, Luis Felipe Torres, director ejecutivo de la Cámara de Bebidas de la Asociación Nacional de Empresarios (Andi), considera que la industria está de acuerdo con que se establezcan en los alimentos y en las bebidas etiquetados frontales para informar mejor a los consumidores: “no nos oponemos estamos de acuerdo, pero el debate es frente a cuál es el etiquetado que debe implementar el país, pues a nivel internacional existen alrededor de 27 modelos de etiquetado, eso lo que muestra es que es un tema bastante variado porque depende de las condiciones y necesidades de cada una de las poblaciones que existen”. En este mismo sentido, Camilo Montes, director ejecutivo de la Cámara de Alimentos de la Andi, precisa que “indiscutiblemente el etiquetado es una herramienta a través de la cual los consumidores acceden a más información fundamental en la construcción de los hábitos de los consumidores y, específicamente, en el caso de los alimentos es la oportunidad de acceder para construir dietas balanceadas”. |  | <https://www.elpais.com.co/economia/etiquetas-en-alimentos-altos-en-azucar-no-pasaron-en-el-congreso-se-debe-insistir.html> | 06-Aug-19 |
| A41 | ANDI ACTA | Media | Information management Discursive strategy | Amplification Frame the debate | Recuerda Montes que desde el año 2016 la Cámara de Alimentos se viene implementando un etiquetado frontal informativo por porciones en términos de la cantidad de nutrientes que son sensibles en términos de salud pública como son las grasas, los azúcares y el sodio. Para Montes se debe dar una discusión para que se implemente lo que realmente le sirve al consumidor colombiano en cuanto al tamaño, el color, la forma, los iconos en términos de las preferencias de los consumidores. Hoy en Colombia no es obligatoria esa información, pero se ha adoptado de forma voluntaria y al menos 4.770 alimentos que hacen parte de la categoría de la industria de los alimentos tienen etiquetado que no es universal y es el GDA, que contiene porcentajes de la tabla nutricional y que actualmente la usan 57 países y que nació en el Reino Unido finalizando los años 90. |  | <https://www.elpais.com.co/economia/etiquetas-en-alimentos-altos-en-azucar-no-pasaron-en-el-congreso-se-debe-insistir.html> | 06-Aug-19 |
| A42 | Coca-Cola | Industry website | Coalition management | Community | Vehiculo atencion de emergencias Mas de 5.800.000 Lt de agua potable han sido entregados desde el 2010 a poblaciones vulnerables y con escasez de este recurso, a traves del vehiculo atencion de emergencias. |  | <https://www.coca-colafemsa.com/presencia/colombia/nuestra-gente-colombia.html> | 13-Aug-19 |
| A43 | Coca-Cola | Industry website | Coalition management Discursive strategy | Community Frame the debate | COLOMBIA Vive bailando es un modelo de intervención social que se enfoca en los adolescentes; a través de clases de baile como una herramienta transformacional, promovemos un estilo de vida sano y un impacto positivo y sostenible en su conducta, liderazgo, unidad familiar y capacidad de cambiar su entorno afectado por la violencia. |  | <https://img.coca-colafemsa.com/assets/files/es/inversionistas/Coca-Cola-FEMSA-Reporte-Anual-2018.pdf> | 13-Aug-19 |
| A44 | Coca-Cola | Media | Direct involvement and influence in policy | Threats (unemployment) | Tras la aprobación de la ley de financiamiento, la multinacional mexicana Coca-Cola Femsa –embotellador en el país de la emblemática marca de gaseosas y refrescos estadounidense– anunció el retiro de 177 empleados en Colombia. Durante el trámite de la reforma tributaria, Coca-Cola Femsa dijo que aumentar los gravámenes a gaseosas tendría efectos contraproducentes para la economía. El Estado recibirá cerca de un billón de pesos anuales por el llamado IVA plurifásico a esos productos y a la cerveza. Según Coca-Cola Femsa Colombia, la compañía se afectó de manera importante, haciendo necesario e inminente ajustar el modelo operativo y la estructura organizacional a las nuevas condiciones tributarias y de mercado. Por su parte, el Sistema Coca-Cola, conformado por The Coca-ColaCompany y Coca-Cola Femsa Colombia, dijo que no renovará su contrato de patrocinio para las selecciones oficiales de fútbol de Colombia, que duró cuatro años.  “Las razones de esta decisión están soportadas tanto en factores de la coyuntura de nuestro sector como en nuestra estrategia de negocio. En ningún caso está relacionada con alguna diferencia con la Federación Colombiana de Fútbol (FCF) o tiene relación alguna con procesos judiciales o investigaciones por parte de la Fiscalía”, sostuvo. | See here as well: https://www.eltiempo.com/economia/empresas/despidos-en-la-planta-de-coca-cola-en-colombia-318518 and https://www.eltiempo.com/economia/empresas/coca-cola-retira-patrocinio-a-la-seleccion-colombia-y-realiza-despidos-318032 | <https://www.eltiempo.com/economia/empresas/los-despidos-en-coca-cola-colombia-por-ley-de-financiamiento-318324> | 06-Aug-19 |
| A45 | Coca-Cola | Twitter | Discursive strategy | Frame the debate | Bebidas de tu lado ‏@bebidasdetulado  22 juil. Dentro del marco de #ApúntateAMoverte, la Corporación Juego y Niñez gracias al apoyo de @CocaColaCol y @FEMSA desarrollo vacaciones recreativas beneficiando a 655 niños en 7 municipios del país. #VidaBalanceada |  | <https://twitter.com/bebidasdetulado/status/1153303684614197250> | 12-Aug-19 |
| A46 | Coca-Cola | Twitter | Discursive strategy | Frame the debate | Bebidas de tu lado @bebidasdetulado  22 juil. #VidaBalanceada: Con el apoyo de la @FundacionFEMSA , la Corporación Juego y Niñez pudo enseñar con Ludonutrición hábitos de vida saludables a más de 4 mil estudiantes. Más información en http://www.bebidasdetulado.com |  | <https://twitter.com/bebidasdetulado/status/1153107393007382529> | 12-Aug-19 |
| A47 | Coca-Cola | Twitter | Discursive strategy | Frame the debate | Bebidas de tu lado @bebidasdetulado Una dieta balanceada y una vida activa son bases del bienestar. Con #JuntosContamos, las bebidas apoyamos este propósito. Visita http://co.juntoscontamos.com #VidaBalanceada #DeTuLado 10:30 - 21 juil. 2019 |  | <https://twitter.com/bebidasdetulado/status/1152994145860034561> | 12-Aug-19 |
| A48 | Coca-Cola | Twitter | Discursive strategy | Frame the debate | Bebidas de tu lado @bebidasdetulado  6 juil. El juego es una herramienta pedagógica fundamental en el aprendizaje. Es por ello que la @FundacionFEMSA financia programas como Ludonutrición. Visita http://www.bebidasdetulado.com para más información sobre una #VidaBalanceada. |  | <https://twitter.com/bebidasdetulado/status/1147497929755619328> | 12-Aug-19 |
| A49 | Coca-Cola | Twitter | Discursive strategy | Frame the debate | Bebidas de tu lado @bebidasdetulado  20 juin Ludonutrición, el programa que promueve hábitos de vida saludables y activos financiado por la @FundacionFEMSA, benefició a más 4 mil niños en Tocancipá, Montería y Cartagena. #VidaBalanceada | Many more Tweet about this | <https://twitter.com/bebidasdetulado/status/1141692175375319040> | 12-Aug-19 |
| A50 | Coca-Cola | Twitter | Discursive strategy | Frame the debate | Bebidas de tu lado ‏@bebidasdetulado  7 juin El proyecto #ApúntateAMoverte durante 2 años logró implementar de manera satisfactoria estratégicas pedagógicas para que 24.000 niños entendieran la importancia y practicaran al menos 60 minutos diarios de actividad física. |  | <https://twitter.com/bebidasdetulado/status/1136981134104047616> | 12-Aug-19 |
| A51 | Coca-Cola | Industry website | Discursive strategy | Frame the debate | Coca-Cola FEMSA esta presente en Colombia hace mas de una decada fortaleciendo su compromiso con el pais a traver de une robusta estrategia de sostenibilidad que genera valor econonomico, social y ambiantal para la tranformacion positiva de las comunidades en Colombia. (...) Con deversas iniciativas y proyectos estrategicos, la compania usca transformar positivamente las comunidades donde opera por medio de la generacion de valor economico, social y ambiental. |  | <https://www.coca-colafemsa.com/presencia/presencia-colombia.html> | 13-Aug-19 |
| A52 | Coca-Cola | Twitter | Information management Discursive strategy | Amplification Frame the debate | Bebidas de tu lado @bebidasdetulado  25 juil.  Enseñar a los niños a cuidar y amar su cuerpo a través del juego, la actividad física, la hidratación y una correcta alimentación fue un propósito de #ApúntateAMoverte. #VidaBalanceada | See here as well: https://twitter.com/bebidasdetulado/status/1152729904561172480 | <https://twitter.com/bebidasdetulado/status/1154390852468219904> | 12-Aug-19 |
| A53 | Coca-Cola | Industry website | Discursive strategy | Frame the debate | Apuntate a moverte El sistema Coca-Cola busca generar habitos duraderos para promover una vida saludable y activa en ninos, de manera que se cumpla con la recomendacion de la Organizacion Mundial de la Salud de moverse al menos 1 hora por dia y satisfacer las necesidades ed alimentacion e hidratacion en el entorno escolar, con la formacion de educadores, padre, 24,000 ninos entre 6 y 12 anos de 10 municipios del pais han sido beneficiados. |  | <https://www.coca-colafemsa.com/presencia/colombia/nuestra-gente-colombia.html> | 13-Aug-19 |
| A54 | Coca-Cola | Industry website | Information management Discursive strategy | Amplification Frame the debate | Educacion alimentaria Es una iniciativa de educacion donde fomentamos estilos de vida activa y saludable y proporcionamos hechos y datos para contribuir a la toma de decisiones informadas a la hora de escoger alimentos. Este proyecto lo desarollamos en asocio con la Fundacion Alpina y consiste en brindar a los mas jovenes informacion util para la toma de decisiones en sus habitos alimenticios. LLas charlas tratan diferentes tematicas como: alimentacion balanceada, educacion nutricional para la toma de decisiones informadas e hidratacion. Ya son 1300 ninos capacitors en localidad de Fontibon en Bogota |  | <https://www.coca-colafemsa.com/presencia/colombia/nuestra-gente-colombia.html> | 13-Aug-19 |
| A55 | Coca-Cola | Industry website | Information management Discursive strategy | Amplification Frame the debate | Charlas de nutricion Desarrollamos espacios informativos, dirigidos a entidades gubernamentales, en los que brindamos informacion sobre el balance energetico y la adecuada hidratacion, contribuyendo a la promocion de estilos de vida activaa y saludable. A la fecha, hemos beneficiado a mas de 900 personas. |  | <https://www.coca-colafemsa.com/presencia/colombia/nuestra-gente-colombia.html> | 13-Aug-19 |
| A56 | Colanta | Twitter | Coalition management | Community | Colanta ‏@ColantaOficial  May 9 Colanta mejora las condiciones educativas de los niños y jóvenes de la IE Tricentenario de Medellín. Invirtió en mobiliario para sus áreas administrativas y comunes, además de mejoras tecnológicas. Colanta hacia la educación con calidad. En alianza con @AlcaldiadeMed |  | <https://twitter.com/ColantaOficial/status/1126542428520034304> | 07-Aug-19 |
| A57 | Colanta | Twitter | Coalition management | Health organisations Community | Colanta Retweeted Alcaldía de Sonsón @sonsonprogresa  Apr 30 Con la vinculación de @ColantaOficial @cornare Cooabejorral, @Bancolombia @Davivienda @BancoAgrario la ESE hospital San Juan de Dios, la mesa municipal de Infancia y Adolescencia, clausuró esta tarde el mes de la niñez🎊🎉 en el parque principal. |  | <https://twitter.com/sonsonprogresa/status/1123371945112866816> | 07-Aug-19 |
| A58 | Colanta | Twitter | Coalition management | Community | Colanta ‏@ColantaOficial  Mar 1 En este 2019 volvimos a hacer esas donaciones que nos hacen felices. Más de 200.000 cuadernos fueron regalados a niños y niñas de Colombia. Colanta Sabe Más, Sabe a Campo. | See the video: branding | <https://twitter.com/ColantaOficial/status/1101521494650306560> | 07-Aug-19 |
| A59 | Colanta | Twitter | Coalition management | Community | Colanta Retweeted Alcaldía de Cereté. @AlcaldiaCerete  Feb 19 Hace pocos días llegamos con @ColantaOficial a Martínez y La Ceibita, donde se distribuyeron los más de 3.500 cuadernos de su donación para niños de 1° a 3° 🧒👧🏻 🤸‍♂️🤸🏻‍♀️ . Aquí les contamos cómo fue la visita de ese día. Seguimos gestionando más aportes como este. 📔#ProgresoSeguro | See the video: branding  See also: https://twitter.com/AlcaldiaCerete/status/1095794565506846721 | <https://twitter.com/AlcaldiaCerete/status/1097938284481785856> | 07-Aug-19 |
| A60 | Colanta | Industry website | Coalition management | Government bodies Community | El espíritu altruista de COLANTA se ratifica también con el apoyo, a través de un distribuidor, al programa “Vaso de leche” de la Alcaldía de Medellín, en escuelas y colegios de la capital antioqueña y que beneficia a 120 mil niños cada día con una bolsita de leche fortificada y adicionada con vitaminas de 200 centímetros cúbicos, para combatir la desnutrición infantil. COLANTA suscribió contrato mediante el cual atiende el Programa Maná de la Gobernación de Antioquia. Son 140 mil niños a los que diariamente se les suministra una ración de leche en polvo saborizada, como golosina complemento nutricional y la cual pueden consumir directamente o diluyéndola en agua. El Ex Gobernador de Antioquia Luis Alfredo Ramos, lo extendió a los 365 días del año. Con el programa del Ex Presidente de La República, Álvaro Uribe, denominado “Desayunos Infantiles” y ejecutado a través del Instituto Colombiano del Bienestar Familiar – ICBF, COLANTA suministra a la población infantil de bajos recursos más 500 mil raciones de leche saborizada líquida y una galleta fortificada. (...) En síntesis COLANTA es un claro ejemplo de lo que se puede lograr gracias al sistema cooperativo y a la solidaridad de asociados, trabajadores y consumidores y a los gobiernos que entienden los beneficios de este sistema como solución con un solo objetivo: contribuir con el desarrollo del campo y el fortalecimiento de la economía del país. (...) Donaciones de Leche en todo el país Durante años COLANTA les ha comprado a los campesinos los excedentes de leche en época de abundancia para distribuirlos gratuitamente a las clases menos favorecidas del país, con prelación a los niños, madres, ancianos y desplazados. En los últimos seis años ha regalado 55 millones de litros de leche en capitales de Colombia como Bogotá, Medellín, Cali, Barranquilla, Armenia. Pereira y Manizales. |  | <https://colanta.com/corporativo/trayectoria/#colanta-solidaria> | 13-Aug-19 |
| A61 | Colanta | Twitter | Coalition management | Government bodies (foreign) | Colanta Retweeted  US Embassy Bogota Verified account @USEmbassyBogota  Jul 22 MT @usaid_colombia: 28 #FuturosLecheros del país se reúnen en #Agroexpo2019 para compartir y aprender de sus procesos productivos. @ColantaOficial @USEmbassyBogota  #AlianzasCompetitivas #YucaAmargaParaUnaDulceLeche #CosecheYVendaALaFija #ElCampoEsDeTodos | See also (and additionnal examples online) https://twitter.com/USAID_Colombia/status/1151882832119312384 | <https://twitter.com/USEmbassyBogota/status/1153319010139869185> | 07-Aug-19 |
| A62 | Colanta | Twitter | Coalition management | Government bodies Media | Colanta Retweeted Radio Más COLANTA @RadiomasColanta  Jul 22 Los integrantes del Equipo Radio Más estamos felices por el éxito del Festival Colanta, expresado por el gerente, Sergio González Villa y la jefa del Departamento de Educación y Promoción Cooperativa, Cecilia Sofía Cardona Escudero. |  | <https://twitter.com/RadiomasColanta/status/1153424584470929409> | 07-Aug-19 |
| A63 | Colanta | Industry website | Information management | Amplification | Frente a esta situación, los científicos y expertos de la salud, no solo reiteran la importancia que tiene incluir leche y productos lácteos en la dieta diaria, sino que también desmienten los mitos que han alejado a cientos de consumidores de este alimento.  De acuerdo con la Fundación Iberoamericana de Nutrición (FINUT), la leche y sus derivados se constituyen como un alimento vital e irremplazable en la dieta tanto de niños, como de adultos, por su alto valor nutricional, ya que es una excelente fuente de proteína, grasas, vitaminas y minerales.  Además de aportar energía, calcio y zinc, nutrientes esenciales para el crecimiento y óptimo desarrollo de todos los seres humanos.  En este sentido, la leche y los productos lácteos son alimentos básicos en una dieta saludable y equilibrada, pues cuenta con nutrientes necesarios para llevar una vida sana.  Por todo lo anterior, es más que válido afirmar que la leche y sus derivados son alimentos irremplazables. Y no debe eliminarse su consumo, a menos que sea por recomendación médica. | Much more examples of amplification with no references to scientific literature: https://colanta.com/sabe-mas/noticias/nutricion/5-mitos-sobre-la-leche-y-su-consumo/ https://colanta.com/sabe-mas/noticias/5-consejos-para-lograr-una-alimentacion-saludable-2/ | https://colanta.com/sabe-mas/noticias/nutricion/toma-leche-sin-miedo-2/ | 13-Aug-19 |
| A64 | Colanta | Industry website | Information management | Amplification | El queso es uno de los derivados lácteos más beneficiosos para la salud, gracias a su aporte de proteínas de alto valor biológico, vitaminas y minerales, como fósforo y calcio. Asimismo, los bajos niveles de lactosa presente en algunos quesos, lo convierten en un alimento de fácil digestión.  El alto contenido de calcio y vitaminas A, B, D, E y K, son indispensables para la sana salud del sistema óseo. El triptófano, aminoácido presente en el queso, aumenta los niveles de serotonina y melatonina, sustancias químicas que ayuda a regular el apetito, estado de ánimo y ciclo del sueño, respectivamente.  Según los últimos estudios publicado por la Universidad de Copenhague, el consumo de queso no aumenta los niveles de colesterol LDL y además ayuda a prevenir la diabetes tipo 2.  Finalmente, el queso puede ayudar a prevenir la aparición de caries, pues su consumo estimula la producción de saliva. Después de todo, la costumbre de comer una pequeña porción de queso a manera de postre, tiene una razón que va más allá de lo puramente gastronómico. |  | <https://colanta.com/sabe-mas/noticias/por-que-es-bueno-comer-queso/> | 13-Aug-19 |
| A65 | Colanta | Industry website | Information management | Amplification | Gracias a su alto valor nutricional, la leche y sus derivados se consideran alimentos indispensables en la dieta de cualquier ser humano, especialmente si es menor de 25 años.  En los últimos años han surgido movimientos que han descalificado este grupo de alimentos y sus propiedades, hasta lograr que cada vez más jóvenes eliminen de su dieta el consumo de leche y productos lácteos.  De acuerdo con la Sociedad Nacional para la Osteoporosis del Reino Unido, los jóvenes menores de 25 que reducen o eliminan de su dieta la leche, yogures y quesos no desarrollan huesos, ni dientes lo suficientemente fuertes para la vida adulta y corren el riesgo de padecer múltiples afecciones óseas, diabetes y sobrepeso en el largo plazo.  Lo que más alarma a los profesionales de la salud, es que las nuevas generaciones al suspender los lácteos no reemplazan la ingesta de calcio, zinc, proteína, grasas y de más nutriente presentes en la leche y sus derivados.  Por lo anterior, los expertos recomiendan no eliminar los lácteos de la alimentación diaria, pues son uno de los alimentos más completos y necesarios para un sano crecimiento y óptimo desarrollo en la juventud. |  | <https://colanta.com/sabe-mas/noticias/dile-no-a-las-dietas-sin-lacteos-2/> | 13-Aug-19 |
| A66 | Colanta | Twitter | Coalition management | Community Governement bodies (President, foreign) | @USAID_Colombia Follow Follow @USAID_Colombia More USAID/Colombia Retweeted Presidencia Colombia Felicitaciones a nuestros aliados @ColantaOficial, @CorpInteractuar, @FRestrepoBarco, @Discovery_Co, @FunCarvajal, @FundacionLuker, @Mineros_sa, @JA_Asociados, @Ideaspaz y #Bancamia por su compromiso con la transformación de los territorios golpeados por la violencia.USAID/Colombia added,  Presidencia Colombia Verified account @infopresidencia Los representantes de las #AlianzasCompetitivas para la Equidad, presentadas este martes en la Casa de Nariño, al lado del Presidente @IvanDuque y el Embajador de EEUU en Colombia, Kevin Whitaker.  8:27 PM - 9 Apr 2019 | Other Tweets available for the same event | <https://twitter.com/USAID_Colombia/status/1115818557282058241> | 07-Aug-19 |
| A67 | Danone | Twitter | Coalition management | Community | Granitos de Paz @Granitosdepaz  30 jul. Agradecemos a @alqueriaoficial la donación de producto para los cartageneros menos favorecidos, vinculados a Granitos de Paz. en Olaya. Rafael Nuñez, Bolívar https://www.instagram.com/p/B0jDoJUA3jW/?igshid=gpen7lu6kl7r … |  | <https://twitter.com/Granitosdepaz/status/1156241522951520256> | 07-Aug-19 |
| A68 | Danone | Twitter | Coalition management | Community | Carlos Enr Cavelier @CavelierE  31 jul. 'Alquería cada año impulsa la campaña Alimenta Compartiendo, al entregar 1,5 millones de vasos leche a 5.200 niños distribuidos a través de 14 bancos de alimentos.' - @larepublica_co   @abacocolombia @BancoAAlimentos | See also: https://twitter.com/CavelierE/status/1154139189329272832 | <https://twitter.com/CavelierE/status/1156600658650210304> | 07-Aug-19 |
| A69 | Danone | Twitter | Coalition management | Health and other organisations | Foros Semana @ForosSemana  29 jul. #CLE2019 \| HOY a las 11:00 a.m. ¿El mundo productivo está conectado con la academia? Conéctese al #FBLive con Andrea Escobar, directora ejecutiva de la Fundación Empresarios por la Educación y Carlos Enrique Calvelier, presidente de @AlqueriaOficial. #LaEducaciónTransforma |  | <https://twitter.com/ForosSemana/status/1155839573559336962> | 07-Aug-19 |
| A70 | Danone | Twitter | Coalition management Discursive strategy | Community Frame the debate | Alquería Cuenta verificada @AlqueriaOficial Seguir Seguir a @AlqueriaOficial Hoy es el día mundial del reciclaje y nosotros lo celebramos sembrando árboles, no olvides que para reciclar nuestras bolsa debes 1. Abrir 2. Enjuagar 3. Reciclar ♻ #VoluntariosAlquería #DíaMundialDelReciclaje  10:27 - 17 may. 2019 |  | <https://twitter.com/AlqueriaOficial/status/1129438229290246144> | 07-Aug-19 |
| A71 | Danone | Twitter | Coalition management Discursive strategy | Community Frame the debate | Alqueria @AlqueriaOficial Seguir Seguir a @AlqueriaOficial Estamos en el #FBLI2019, congreso que reúne a Bancos de Alimentos del mundo para reducir el hambre y el desperdicio alimentario. Somos conscientes de la importancia de nuestro rol en la cadena alimentaria y de nuestro compromiso para erradicar el hambre en Colombia. #CeroHambre  9:45 - 28 mar. 2019  Alquería Cuenta verificada @AlqueriaOficial  28 mar. Más #HambreCero #CeroDesperdicios #CeroHunger #ZeroWaste #TacklingFoodWaste #FoodBanks #FoodHeroes #FightingHunger #AlqueríaEsMiOrgullo 🥛🌎🌍🌏 🥛 |  | <https://twitter.com/AlqueriaOficial/status/1111308340905328642> | 07-Aug-19 |
| A72 | Danone | Twitter | Coalition management Discursive strategy | Community Frame the debate | Alquería Compte certifié @AlqueriaOficial Hoy junto con el @BancoAAlimentos y nuestros #VoluntariosAlquería vivimos una jornada con los niños de la Fundación La Esperanza en La Calera, enseñamos prácticas de cuidado del medio ambiente y compartimos en un espacio de recreación para ellos. 👦🏻🥰🧒🏻  08:17 - 9 mars 2019  Alquería Compte certifié @AlqueriaOficial  9 mars Gracias al @BancoAAlimentos por acompañarnos en esta jornada. #ÚneteALosHéroes 🙌🏻 |  | <https://twitter.com/AlqueriaOficial/status/1104415855180419072> | 07-Aug-19 |
| A73 | Danone | Twitter | Coalition management Discursive strategy | Community Frame the debate | Alquería a retweeté Asoc.BancoAlimentos @abacocolombia  4 mars @AlqueriaOficial es una aliado en la nutrición infantil, a través del Programa Desayuno Saludables 11 toneladas de leche llegan a 8 ciudades del país, alimentando a 3.400 niños en situación de vulnerabilidad.  Comprometidos con el desarrollo de los más vulnerables. |  | <https://twitter.com/abacocolombia/status/1102656398968664066> | 07-Aug-19 |
| A74 | Danone | Twitter | Coalition management Information management | Community Amplification | Alquería Cuenta verificada @AlqueriaOficial  10 jun. ¡LO LOGRAMOS! Gracias a ustedes llegamos a 1.5 millones de visualizaciones en nuestros videos del #DíaMundialdelaLeche, 🥛 hoy la magia se vuelve realidad y más de 5.000 niños en Colombia podrán disfrutar de un vaso de leche. 👦💖#LaNutriciónEsParaSiempre #AlimentaCompartiendo |  | <https://twitter.com/AlqueriaOficial/status/1138226611063590913> | 07-Aug-19 |
| A75 | Danone | Twitter | Coalition management Information management | Community Amplification | Banco de Alimentos de Bogotá ‏ @BancoAAlimentos Seguir Seguir a @BancoAAlimentos Más Banco de Alimentos de Bogotá Retwitteó Alquería En el #DiaMundialDeLaLeche, junto a @AlqueriaOficial celebramos que estamos #JuntosContraElHambreBanco de Alimentos de Bogotá agregado,  0:12 Alquería Cuenta verificada  @AlqueriaOficial Sigamos construyendo historia cada 1 de Junio en el #DíaMundialDeLaLeche, 🥛mira este video completo 👉 http://bit.ly/RayDMDL y dona un vaso de leche para alimentar diariamente a los niños de Colombia. 👦💖👧 … 15:57 - 1 jun. 2019 desde Chaparral, Colombia |  | <https://twitter.com/BancoAAlimentos/status/1134957230757990401> | 07-Aug-19 |
| A76 | Danone | Twitter | Coalition management Information management | Community Amplification | Fundación SACIAR @FundacionSaciar Seguir Seguir a @FundacionSaciar Más Fundación SACIAR Retwitteó Alquería Celebremos que la #NutriciónEsParaSiempre   Hoy en el #DíaMundialDeLaLeche @FundacionSaciar se une a @AlqueriaOficial además para agradecerles y felicitarles por la gestión y bondadosa labor con el @BancoAAlimentos  #AlimentaCompartiendo #ÚneteALosHéroes #HambreCero #ZeroHungerFundación SACIAR agregado,  0:12 Alquería Cuenta verificada   @AlqueriaOficial Sigamos construyendo historia cada 1 de Junio en el #DíaMundialDeLaLeche, 🥛mira este video completo 👉 http://bit.ly/RayDMDL y dona un vaso de leche para alimentar diariamente a los niños de Colombia. 👦💖👧 … 14:01 - 1 jun. 2019 |  | <https://twitter.com/FundacionSaciar/status/1134928089807511552> | 07-Aug-19 |
| A77 | Danone | Twitter | Coalition management Information management | Community Amplification | Juan Carlos Buitrago Ortiz ‏@juanbuitrago_  1 jun. Hoy en el día mundial de la leche, @AlqueriaOficial hace una donaciòn a 14 bancos de alimentos de 1.500.000 vasos de leche para mejorar la alimentaciòn de niños en situaciòn de vulnerabilidad! @abacocolombia @CavelierE @CamiloMontes @carolinachica @BancoAAlimentos @Nutricion2030 | Big event, see also: https://twitter.com/AlqueriaOficial/status/1134817215767875589 https://twitter.com/AlqueriaOficial/status/1134514704599179269 https://twitter.com/BancoAAlimentos/status/1134506291068641281 https://twitter.com/BancoAAlimentos/status/1134185913179955201 https://twitter.com/AlqueriaOficial/status/1133516275119005696 | <https://twitter.com/juanbuitrago_/status/1134911280320565248> | 07-Aug-19 |
| A78 | Danone | Twitter | Direct involvement and influence in policy Discursive strategy | Lobby Frame the debate | Alquería a retweeté  Juan Carlos Buitrago Ortiz @juanbuitrago_  13 mars Presentamos a @ICBFColombia @JPungiluppi avances de la Alianza por la Nutrición Infantil. Trabajo conjunto: @ANDI_Colombia + @abacocolombia + @Fundacion_Exito  Iniciamos plan de trabajo con Gobierno!  @CamiloMontes @CavelierE @AlqueriaOficial @CpriveraMarin @AsebiolA |  | <https://twitter.com/juanbuitrago_/status/1105928511942987777> | 07-Aug-19 |
| A79 | Danone | Industry website | Information management | Amplification | 5 razones por las que toda familia debe tomar leche La humanidad ha consumido leche desde tiempos inmemoriales. La lista de sus beneficios es larga. Aquí mostramos cinco de ellos. El consumo de leche y sus derivados se ha extendido por todo el mundo desde los inicios de la humanidad. Sus beneficios se han intuido desde temprano en la historia, pero con el desarrollo en las últimas décadas de las ciencias de la nutrición se conocen con más exactitud sus aportes a la salud. La leche es fuente de vitaminas y minerales, de proteínas para los músculos y de carbohidratos como energía. Es ideal para mantener el esqueleto en buenas condiciones, gracias a que es uno de los alimentos con mayor cantidad de calcio por porción. Es además un alimento versátil y la tecnología ha ampliado la variedad de sus presentaciones: entera, descremada, deslactosada, enriquecida, orgánica, con sabores… La lista de beneficios de la leche es larga. Aquí mostramos cinco de ellos. (...) Es fuente de proteínas Los aminoácidos son la base de las proteínas y estas, a su vez, forman los músculos. La leche contiene aproximadamente ocho gramos de proteínas por porción (una taza) y casi todos los aminoácidos esenciales (los que el cuerpo no puede sintetizar por sí mismo y, por lo tanto, hay que obtenerlos de los alimentos); de allí que la leche sea un alimento clave para mantener y hacer crecer los músculos. (...) Una mayor musculatura acelera el metabolismo y, con ello, el consumo de calorías, que a su vez mantiene bajo control el peso corporal. Cuando se ingiere leche descremada, a los beneficios de las proteínas se les añaden los de un menor consumo de grasas (la leche entera tiene alrededor de 4% de grasa por porción, mientras que la descremada se reduce a 1% o menos). (...) Está vinculada con el control de la diabetes Varios estudios científicos han confirmado que la leche puede reducir la incidencia de diabetes tipo 2, que se desarrolla en la adultez. Se trata del estudio de las mujeres saludables (Healthy Women’s Study), el estudio de las enfermeras (Nurses Study) y el Estudio Multiétnico sobre la Ateroesclerosis (MESA, por sus siglas en inglés), los tres llevados a cabo en Estados Unidos; en Europa, la Investigación Europea Prospectiva sobre Cáncer y Nutrición (EPIC, en inglés). El efecto benéfico de la leche para reducir la diabetes se debe a que la lactosa (el azúcar de la leche) induce al páncreas a producir menos insulina, en contraste con otros alimentos, que tienen glucosa. Es un alimento para todos La leche es beneficiosa para personas de cualquier edad y un alimento que se consume casi en su estado original y con un mínimo procesamiento. También contiene agua, por lo que ayuda a prevenir que el cuerpo se deshidrate. En el caso de los niños, la incorporación de la leche a su dieta ayuda a fortalecer su sistema inmunológico y favorece el aumento de la estatura: por cada 245 mililitros de leche al día que se añaden a la dieta, un niño puede crecer 0,4 centímetros adicionales al año, de acuerdo con el documento “La leche como vehículo de salud de la población”, publicado por la Federación Española de la Nutrición y por la Fundación Iberoamericana de Nutrición. | See website for more info Federación Española de la Nutrición cited here is funded by the food industry, including dairy companies: http://www.fen.org.es/index.php/sobre-fen/promotores | <https://www.mundoalqueria.co/5-razones-las-toda-familia-tomar-leche/> | 15-Aug-19 |
| A80 | Danone | Industry website | Information management | Amplification | Lácteos en el desayuno: ¿una estrategia para controlar el hambre y el peso? Eliminar las calorías de tu dieta no es la solución para bajar de peso. Los lácteos en el desayuno son la opción ideal a incluir en esta comida. Elegir alimentos nutritivos en el desayuno y que generen saciedad es fundamental para controlar la ingesta calórica total en el día. Además, la evidencia demuestra que los estudiantes acostumbrados a desayunar, obtienen mejores resultados académicos, mejoran la memoria y la atención. Concluyeron el estudio recomendando un desayuno diario basado en alimentos variados, principalmente ricos en fibra, granos integrales, frutas y productos lácteos desnatados (Gail Rampersaud et al, 2005). La leche de vaca aporta múltiples nutrientes como carbohidratos, grasas, proteínas, vitaminas y minerales, haciendo de este un alimento completo. Gracias a que proporciona proteína y grasas, la leche tiene una digestión más lenta en el estómago por lo que tiene la capacidad de hacernos sentir más llenos, es decir que genera mayor sensación de saciedad. En un estudio publicado en 2016, en la Revista Europea de Nutrición, se evaluó el efecto del consumo de leche en niños con obesidad en edades entre los diez y doce años, encontrando que esta producía mayores niveles de saciedad o sensación de llenura, lo cual se considera una estrategia válida para el control del apetito y, por ende, para el control del peso. (Sanaz Mehrabani, 2016). Asimismo, Dove y colaboradores en su artículo publicado en 2009, en la Revista Americana de Nutrición Clínica, reportan que el consumo de leche de vaca en horas de la noche produce mayor saciedad que la producida por los jugos de fruta (Dove, 2009). Teniendo en cuenta estos estudios te invitamos a que la bebida elegida para el desayuno sea a base de lácteos, ya que no solamente aporta gran cantidad de nutrientes esenciales para el cuerpo, sino que además contribuye al control de saciedad a lo largo del día por lo que puede ser una gran estrategia para el control del peso. | See also other example here: https://www.mundoalqueria.co/importancia-la-leche-los-escolares/ | <https://www.mundoalqueria.co/bebidas-lacteas-desayuno/> | 15-Aug-19 |
| A81 | Danone | Industry website | Information management Discursive strategy | Amplification Frame the debate | Alimentación Un estilo de vida balanceado y lleno de bienestar comienza por una buena alimentación, por eso Alquería te trae consejos, ideas y combinaciones deliciosas para que puedas mejorar tus rutinas de alimentación sin complicaciones, alimentación Alquería.  ¡Conoce más del mundo de alimentación que Alquería por tu Bienestar trae para ti! Cuando escuchar hablar de alimentación es probable que vengan a tu cabeza palabras como saludable, nutrición y bienestar. Y en la actualidad esto es lo más común, porque día a día vamos cambiando nuestra forma de pensar y de cuidar nuestra dieta. ¡Cada vez somos más conscientes de lo que verdaderamente necesita nuestro organismo! Una alimentación balanceada y saludable. Así que si andas en búsqueda de recomendaciones e información valiosa que beneficie tu cuerpo y tu bienestar, llegaste al lugar indicado, porque en Alquería Por Tu Bienestar entendemos que lo que necesitas son ideas, consejos, combinaciones balanceadas y deliciosas para que puedas hacer un cambio y tener una rutina de alimentación llena de bienestar y sin complicaciones. Entra y regístrate aquí para recibir contenido nuevo e interesante y así no te perderás ninguna de las mejores notas de Alquería por tu bienestar. | See website for specific examples of amplification: http://www.alqueriaportubienestar.com/blog/estilo-de-vida http://www.alqueriaportubienestar.com/blog/alimentacion/ | <http://www.alqueriaportubienestar.com/blog/alimentacion> | 15-Aug-19 |
| A82 | Exito | Industry website | Coalition management | Health organisations and government bodies Internal | Alianza por la nutrición infantil Martes, 5 Marzo 2019  Evento de oficialización Bogotá, martes 5 de marzo de 2019  Como símbolo del compromiso ineludible e inaplazable y la corresponsabilidad que asiste al Estado, la sociedad y la familia, el despacho de la Primera Dama de la Nación y la Fundación Éxito, líder del movimiento nacional Gen Cero, que busca la primera generación con cero desnutrición crónica en Colombia para el 2030, unen ideales y articulan capacidades y acciones en la “Alianza por la nutrición infantil”.  Las partes representan la necesaria articulación del sector público y privado para favorecer los derechos de los niños, llamando la atención sobre los fundamentales que parten desde la nutrición, como eje del desarrollo físico, emocional y cognitivo de los seres humanos. |  | <https://www.grupoexito.com.co/es/sala-de-prensa/noticias/alianza-por-la-nutricion-infantil> | 21-Aug-19 |
| A83 | Exito | Industry website | Coalition management | Internal | ¿Quiénes son nuestros aliados? Alcaguete Alsea Belleza Express SA Coca-Cola FEMSA Genoma Lab De mi pueblo |  | <https://www.fundacionexito.org/nuestros-aliados/sector-privado> | 21-Aug-19 |
| A84 | Exito | Industry website | Coalition management | Health organisations and government bodies Internal | ¿Quiénes son nuestros aliados Gen Cero? Alcaldías Gobernaciones Ministerio de salud ICBF SNBF DNP Procuraduría  Niñez Ya Fundaciones |  | <https://www.fundacionexito.org/nuestros-aliados/sector-publico> | 21-Aug-19 |
| A85 | Exito | Twitter | Coalition management | Community | Fundación Éxito a retweeté  Sinfónica de Antioquia @SinfonicaAnt  23 avr. Con nuestro programa de Iniciación Musical en distintas instituciones educativas hemos buscado que niños y niñas del país se acerquen a la música de forma creativa e inventiva. ¡Nos emociona tener a más de 1.100 beneficiados! 🧒🏽👧🏼🧒🏻 ¡Gracias @Fundacion_Exito! | See more tweet on the twitter account | <https://twitter.com/SinfonicaAnt/status/1120705319632609280> | 21-Aug-19 |
| A86 | Exito | Twitter | Coalition management | Community | Fundación Éxito a retweeté  San Vicente Fundación @sanvicentefund  28 janv. Gracias a la alianza con @Fundacion_Exito, en estos últimos 6 meses, más de 2.000 niños entre 6 meses y 5 años de nuestro #HospitalInfantil, han contado con apoyo para tener una media mañana que aporta a una recuperación exitosa basada en una nutrición completa y saludable. |  | <https://twitter.com/sanvicentefund/status/1090021876011810817> | 21-Aug-19 |
| A87 | Exito | Industry website | Coalition management Discursive strategy | Community Frame the debate | Las madres gestantes y los niños que son atendidos por la Fundación Éxito deben hacer parte de otra oferta de asistencia alimentaria del Estado (Hogares FAMI del ICBF, modalidad de Entorno Familiar del ICBF, programas de Alcaldías municipales, entre otros). La Fundación Éxito complementa las diferentes ofertas disponibles en los territorios para la población con riesgo nutricional. Son priorizadas las familias que presentan mayo vulnerabilidad socioeconómica  Entidades creadas para recolectar, pasteurizar y almacenar leche materna y ofrecerla a los niños prematuros o con bajo peso al nacer que están hospitalizados. la Fundación Éxito apoya a los 15 bancos de leche humana en el país. Esta ayuda incluye: Formación del talento humano Redotación de equipos. Implementación y/o fortalecimiento de sistemas de información y formulación de los lineamientos de bancos de leche.  El Grupo Éxito a través de la Fundación Éxito, dona comestibles y no comestibles que son aptos para el consumo humano, pero no para la venta. Así el Banco de Alimentos logra beneficiar a miles de niños y familias en situación de vulnerabilidad con sus programas de alimentación y apoyo integral. | See also: https://www.fundacionexito.org/como-operamos | <https://www.fundacionexito.org/nuestros-beneficiarios> | 21-Aug-19 |
| A88 | Exito | Twitter | Coalition management Discursive strategy | Community Frame the debate | Fundación Éxito a retweeté  Asoc.BancoAlimentos ‏@abacocolombia  11 juil. Plus Los 19 Bancos de Alimentos del país se encuentran en Nutrium, una empresa que nos ayuda a conocer el proceso de transformación de frutas; un proceso innovador que ayudará a intensificar el rescate de de alimentos en REAGRO.  #innovacion #Alimentos  @Fundacion_Exito | Several Tweets for this - see account | <https://twitter.com/abacocolombia/status/1149352193637662720> | 21-Aug-19 |
| A89 | Exito | Twitter | Coalition management Discursive strategy | Health organisations and government bodies Frame the debate | Fundación Éxito a retweeté  LuisGuillermo Patiño ‏@LuisGPatino  10 avr. @Fundacion_Exito y @AlcaldiadeMed, a través de @tejiendohogares y el programa @Buen_Comienzo, impulsan la Gran Alianza por la Nutrición Infantil por medio de la cual se busca una primera generación de niños con cero desnutrición crónica en Colombia para el 2030 \| @FicoGutierrez |  | <https://twitter.com/LuisGPatino/status/1116017331556290560> | 21-Aug-19 |
| A90 | Exito | Twitter | Coalition management Discursive strategy | Health organisations and government bodies Frame the debate | Fundación Éxito a retweeté Alex Camacho Erazo @ALEXCAMINA  2 avr. Plus Alex Camacho Erazo a retweeté Seguridad Alimentaria y Nutricional Cali Alianza de la Secretaría de Salud Pública con Fundación éxito, permitirá aportes significativos por la nutrición de los niños y las madres gestantes en Cali. @AlcaldiaDeCali @MauriceArmitage @NelsonSinister1 @SaludCali @Fundacion_Exito #Caliprogresacontigo | See more info on the Twitter account | <https://twitter.com/ALEXCAMINA/status/1113133345607704579> | 21-Aug-19 |
| A91 | Exito | Twitter | Coalition management Discursive strategy | Health organisations and government bodies Frame the debate | Fundación Éxito a retweeté  Fernando Carrillo F. ‏ Compte certifié @fcarrilloflorez  2 avr. Firmamos pacto con la @Fundacion_Exito para prevenir y erradicar la desnutrición infantil en #Colombia, una iniciativa que representa compromiso y voluntad por acabar con este dramático flagelo. Estas son las batallas éticas que deben unirnos como país | Fernando Carrillo F.Compte certifié @fcarrilloflorez Procurador General de la Nación. @PGN_COL Constituyente de 1991. | <https://twitter.com/fcarrilloflorez/status/1112902013715705856> | 21-Aug-19 |
| A92 | Exito | Twitter | Coalition management Discursive strategy | Health organisations and government bodies Frame the debate | Fundación Éxito  @Fundacion_Exito  2 avr. “Que la @PGN_COL [Procurador General] nos esté apoyando es un orgullo, le da importancia y contundencia al problema para ponerlo en la mente de los de los gobernantes y de todos los colombianos.” - Gonzalo Restrepo López, Presidente Junta Directiva Fundación Éxito #GenCero |  | <https://twitter.com/Fundacion_Exito/status/1112898869455511552> | 21-Aug-19 |
| A93 | Exito | Twitter | Coalition management Discursive strategy | Health organisations and government bodies Frame the debate | Fundación Éxito a retweeté  NiñezYA – Sociedad Civil por los NNA @Ninez_Ya  8 mars Esta estrategia convoca a todos los sectores del país para que se unan a la Gran Alianza por la Nutrición de la Niñez, estrategia que se inició con la participación de la Consejería Presidencial para la Niñez y la Adolescencia y @Fundacion_Exito |  | <https://twitter.com/Ninez_Ya/status/1104064239424036864> | 21-Aug-19 |
| A94 | Exito | Twitter | Coalition management Discursive strategy | Health organisations and government bodies Frame the debate | Fundación Éxito a retweeté  Consejería Niñez y Adolescencia ‏@ConsejeriaNinez  5 mars Plus Hoy lanzamos la Gran Alianza por la Nutrición y nuestra primera acción la hacemos en conjunto con @Fundacion_Exito y @MinSaludCol. Trabajaremos para que los niños y niñas tengan el mejor comienzo de vida. ¡Únete! | Many more Tweets for this event as well - see Twitter account | <https://twitter.com/ConsejeriaNinez/status/1103042230489620480> | 21-Aug-19 |
| A95 | Exito | Twitter | Coalition management Discursive strategy | Health organisations and government bodies Frame the debate | Fundación Éxito a retweeté  Alcaldía de Soacha ‏@Alcaldia_Soacha  5 févr. #Soacha y Fundación Éxito firman convenio para combatir la desnutrición infantil 👍  Ver más: https://bit.ly/2REcsUT |  | <https://twitter.com/Alcaldia_Soacha/status/1092823974982152192> | 21-Aug-19 |
| A96 | Exito | Twitter | Coalition management Information management | Health organisations Amplification | Fundación Éxito a retweeté  Fundación Santa Fe de Bogotá ‏   @FSFB_Salud  9 juil. Esta semana, el equipo de #SaludPoblacional está en Cartagena dictando el curso integrado de consejería en alimentación del lactante y niño pequeño. En el marco de la alianza con @MinSaludCol y @Fundacion_Exito, certificaremos a 13 profesionales de la salud. |  | <https://twitter.com/FSFB_Salud/status/1148684350122332162> | 21-Aug-19 |
| A97 | Exito | Twitter | Coalition management Information management | Health organisations Amplification | Fundación Éxito a retweeté  Fundación Santa Fe de Bogotá ‏@FSFB_Salud  26 juin Plus #SaludPoblacional. En alianza con @MinSaludCol y @Fundacion_Exito, hemos capacitado a 45 funcionarios públicos y jefes de programas de Copacabana, Barranquilla y Cartagena, en herramientas para la inteligencia epidemiológica: cartografía y sistemas de información. | More tweets on this - see Twitter account | <https://twitter.com/FSFB_Salud/status/1143980169704693763> | 21-Aug-19 |
| A98 | Exito | Twitter | Coalition management Information management | Health organisations Amplification | Fundación Éxito  @Fundacion_Exito  23 mai Iniciamos la presentación de los avances del proyecto para la primera Guía de Atención Integral para la desnutrición crónica en alianza con @Bogota @integracionbta @FSFB_Salud @SectorSalud @ICBFColombia #GenCero | More tweets on this - see Twitter account | <https://twitter.com/Fundacion_Exito/status/1131665269695365120> | 21-Aug-19 |
| A99 | Exito | Twitter | Coalition management Information management | Health organisations Amplification | Fundación Éxito a retweeté  Fundación Santa Fe de Bogotá  @FSFB_Salud  21 mai En el marco del #DíaMundialdelaDonacióndeLecheHumana resaltamos la alianza público privada con @MinSaludCol y @Fundacion_Exito que ha permitido influir positivamente en la salud materna e infantil del país. |  | <https://twitter.com/FSFB_Salud/status/1130976717844230146> | 21-Aug-19 |
| A100 | Exito | Twitter | Coalition management Information management | Media Amplification | Fundación Éxito ‏@Fundacion_Exito  16 mai Nos encontramos con algunos de los medios comunicación más importantes del país hablando sobre la importancia de la nutrición para el desarrollo del cerebro #GenCero |  | <https://twitter.com/Fundacion_Exito/status/1129044089536745472> | 21-Aug-19 |
| A101 | Exito | Twitter | Coalition management Information management | Health organisations Amplification | Fundación Éxito @Fundacion_Exito  7 mai Aida Milena Gutiérrez de @MinSaludCol destaca el trabajo intersectorial al referirse a la primera alianza público privada con la @Fundacion_Exito que data desde el 2017 #CongresoLactanciaMaterna @infopresidencia @ConsejeriaNinez |  | <https://twitter.com/Fundacion_Exito/status/1125770659047661571> | 21-Aug-19 |
| A102 | Exito | Twitter | Coalition management Information management | Health organisations Amplification | Fundación Éxito @Fundacion_Exito  7 mai Nos enorgullece hacer parte de la Gran Alianza por la Nutrición Infantil, y agradecemos a @MinSaludCol, la Asociación Nacional de Neonatologia ASCON y la Fundacion Universitaria Sanitas - Paula Escobar @infopresidencia @ConsejeriaNinez @MinSaludCol #CongresoLactanciaMaterna | See also other example on account: https://twitter.com/Fundacion_Exito/status/1125760931034796032 | <https://twitter.com/Fundacion_Exito/status/1125767221760921600> | 21-Aug-19 |
| A103 | Exito | Industry website | Coalition management Information management Discursive strategy | Community Amplification Frame the debate | La atención integral: Es eje transversal de nuestro modelo de intervención con el propósito de mejorar las condiciones para el crecimiento y desarrollo del niño en sus mil primeros días de vida. Esta atención se compone de:  Nutrición:  - Promoción de prácticas saludables en nutrición, higiene, salud, crianza, cuidado y lactancia materna. - Realización de tamizajes antropométricos y seguimiento nutricional periódico.  Salud: - Afiliación de los beneficiarios al Sistema general de Seguridad Social y acceso efectivo a los servicios de salud (control prenatal, crecimiento y desarrollo, consumo de micronutrientes y vacunación)   Desarrollo familiar: Acompañamiento a la familia para acercarla a la oferta disponible en el territorio en temas de educación formal, emprendimiento, empleo y garantía de derechos.  Potencial social - Implementación de rutas de atención para los casos de riesgo sicosocial y los casos de malnutrición. |  | <https://www.fundacionexito.org/modelo-intervencion> | 21-Aug-19 |
| A104 | Exito | Industry website | Coalition management Discursive strategy | Community Frame the debate | Incentivar una vida sana En el Grupo Éxito reconocemos nuestro importante papel en la alimentación, la salud y la nutrición de nuestros grupos de interés, por esta razón nos comprometemos y aportamos a través de las siguientes estrategias:  Política de Nutrición Grupo Éxito  1. Gen Cero: estrategia gestionada por la Fundación Éxito, su principal objetivo es lograr que ningún niño menor de 5 años sufra de desnutrición crónica en Colombia al año 2030. 2. Lugar de trabajo saludable: promovemos la salud integral de nuestros empleados a través de programas de promoción de la salud y prevención de la enfermedad. 3. Sostenibilidad de la cadena alimentaria: apoyamos el desarrollo de pequeños productores y pymes proveedoras de nuestras marcas propias, intercambiamos y ponemos al servicio la información necesaria para la co-innovación y desarrollo de nuevos productos con beneficios nutricionales y de salud. 4. Alimentos nutritivos, saludables y accesibles: brindamos alternativas de productos saludables y nutritivos, que responden a las necesidades nutricionales y de salud. Facilitamos acceso a través de nuestras marcas propias, productos con precios bajos y adaptados a las necesidades de nuestros clientes. 5. Educación alimentaria y nutricional a clientes: educamos en alimentación y nutrición para ayudar a la toma de decisiones alimentarias más saludables. |  | <https://www.grupoexito.com.co/es/sostenibilidad> | 21-Aug-19 |
| A105 | Exito | Twitter | Discursive strategy | Frame the debate | Grupo Éxito ‏@Grupo_Exito  12 avr. En 2018 más de 63.400 niños fueron beneficiados con una mejor #nutrición en #Colombia, gracias al trabajo de @Fundacion_Exito |  | <https://twitter.com/Grupo_Exito/status/1116749530777649153> | 21-Aug-19 |
| A106 | Exito | Twitter | Discursive strategy | Frame the debate | Grupo Éxito @Grupo_Exito  5 avr. No te pierdas el cubrimiento del evento de la feria #CarullaEsSaludable en http://www.instagram.com/placerescarulla y conoce la programación de las experiencias que te ayudará a llevar un estilo de vida activo y sano. | See also: https://twitter.com/Grupo_Exito/status/1113917973285277697 https://twitter.com/Grupo_Exito/status/1113843022016413708 https://www.grupoexito.com.co/es/sala-de-prensa/noticias/celebracion-del-dia-mundial-de-la-actividad-fisica-con-la-primera-manana-de-bienestar https://www.grupoexito.com.co/es/sala-de-prensa/noticias/vida-sana-nutricion-y-actividades-deportivas-en-el-marco-del-dia-mundial-de-la-actividad-fisica-y-el-dia-mundial-de-la-salud | <https://twitter.com/Grupo_Exito/status/1114222637172121601> | 21-Aug-19 |
| A107 | Exito | Twitter | Discursive strategy | Frame the debate | Grupo Éxito ‏@Grupo_Exito  14 mar Nuestra @Fundacion_Exito trabaja por la nutrición de los niños y estamos seguros que esta prioridad es el camino para combatir la desnutrición crónica sobre todo en los primeros 1000 días y así construir juntos un país con mejores oportunidades #GenCero #GoticasGenCero |  | <https://twitter.com/Grupo_Exito/status/1106235272138764290> | 21-Aug-19 |
| A108 | Exito | Industry website | Discursive strategy | Frame the debate | En el Mes de la Niñez, nos la jugamos por la nutrición infantil Viernes, 5 Abril 2019  29 marcas se unen a Gen Cero para celebrar el mes de la niñez y recreación en beneficio de la nutrición infantil.  5 de abril de 2019. Cuando un niño cuenta con una adecuada alimentación y con bienestar emocional, crece con todas las condiciones para transformar el mundo. En el Mes de la Niñez y la Recreación, en la Fundación Éxito nos la jugamos por la nutrición infantil, porque estamos convencidos de que el juego es uno de los principales alimentos del alma, y que cuando un niño cuenta con todas las condiciones nutricionales, crece con un corazón sano y feliz. |  | <https://www.grupoexito.com.co/es/sala-de-prensa/noticias/en-el-mes-de-la-ninez-nos-la-jugamos-por-la-nutricion-infantil> | 21-Aug-19 |
| A109 | Exito | Industry website | Discursive strategy | Frame the debate | Goticas que suman para alimentar a miles de niños colombianos Martes, 5 Febrero 2019  Las Goticas son donaciones voluntarias que los clientes del Grupo Éxito realizan, y que se suman cada año a los recursos gestionados por la Fundación Éxito para llevar una mejor alimentación a los niños que sufren desnutrición crónica en Colombia. En 2019 sus protagonistas son 6 niños que representan a los miles que necesitan este apoyo para crecer sanos, como es su derecho. (...) En 2018 las Goticas junto a los vueltos y a las alcancías se sumaron a otros recursos para beneficiar 63.487 niños de 123 municipios y 27 departamentos colombianos. Estas donaciones superaron los $7.440 millones el año pasado. Junto con el reciclaje donado por el Grupo Éxito que superó los 12.338 millones, son una importante fuente de recaudo que permite a la Fundación Éxito invertir en la nutrición infantil para cumplir su mega Gen Cero: la primera generación en Colombia con cero desnutrición crónica para el 2030 en menores de 5 años. | See more info on the webpage See also: https://www.grupoexito.com.co/es/sala-de-prensa/noticias/con-las-goticas-de-navidad-y-otras-fuentes-solidarias-3-200-ninos-se-alimentaran-mejor-todo-el-2019 | <https://www.grupoexito.com.co/es/sala-de-prensa/noticias/goticas-que-suman-para-alimentar-a-miles-de-ninos-colombianos> | 21-Aug-19 |
| A110 | Exito | Twitter | Discursive strategy | Frame the debate | Fundación Éxito a retweeté  FAN @FundacionFAN  31 juil. El próximo viernes 02 de agosto, viviremos la Lactatón Nacional Gen Cero.  La @FundacionFAN , hará parte de esta gran movilización y pondrá como eje fundamental la Lactancia, ese acto de amor líquido que deberían recibir todos los niños de nuestro país. @Fundacion_Exito #Lactaton | Much much more examples for this event on Twitter | <https://twitter.com/FundacionFAN/status/1156578849544712192> | 21-Aug-19 |
| A111 | Exito | Twitter | Discursive strategy | Frame the debate | Fundación Éxito ‏  @Fundacion_Exito  4 févr. Plus Niñoooos ya casi llegan los cajeros! Los #CajerosGenCero ¿Quiénes son ellos? ¿Por qué están aquí? Son aliados incondicionales de los niños y estarán en Cartagena para seguir dejando huella... #GenCero #GoticasGenCero | See pictures: branding | <https://twitter.com/Fundacion_Exito/status/1092530504031719425> | 21-Aug-19 |
| A112 | Exito | Industry website | Information management | Amplification | Se abre la convocatoria del máximo reconocimiento para quienes en Colombia trabajan por la Nutrición infantil Martes, 5 Febrero 2019  El Premio por la Nutrición Infantil promovido por la Fundación Éxito convoca a las instituciones públicas y privadas que desde diferentes sectores actúan para mejorar la nutrición de los niños en sus primeros 1.000 días de vida como contribución a la causa nacional Gen Cero que busca erradicar la desnutrición crónica para el año 2030.  Desde el 1 de febrero y hasta el 29 de marzo la Fundación Éxito recibirá y revisará los proyectos, iniciativas y trabajos que promueven el crecimiento sano de los niños menores de 2 años en las categorías que se evaluarán por postulación y hasta el 30 de junio aquellas categorías por monitoreo. |  | <https://www.grupoexito.com.co/es/sala-de-prensa/noticias/se-abre-la-convocatoria-del-maximo-reconocimiento-para-quienes-en-colombia-trabajan-por-la-nutricion-infantil> | 21-Aug-19 |
| A113 | FENALCO | Industry website | Direct involvement and influence in policy | Conflict of interest | Guillermo Botero Nieto MINISTRO DE DEFENSA NACIONAL (...) Desde noviembre de 2003 hasta julio de 2018, ocupó la Presidencia de la Federación Nacional de Comerciantes (Fenalco), entidad a la cual ha estado vinculado como miembro de su Junta Directiva en diferentes oportunidades desde 1985. |  | <https://id.presidencia.gov.co/gobierno/mindefensa> | 31-Jul-19 |
| A114 | ILSI | Industry website | Coalition management | Internal | Consejo Directivo Regional ILSI Nor-Andino 2018-2020 Directora Académica, Ana Silvia Bermúdez, Química, Master of Science, Profesora pensionada de la Universidad Nacional de Colombia  Miembros academicos *(information here from Colombians only)* Valentina Guzmán, Pontificia Universidad Javeriana  Miembros industria *(information here from Colombians only)* Presidente Regional y Representante Legal ILSI Nor-Andino: Juan Carlos Farfán, Nestlé de Colombia Tesorero ILSI Nor-Andino: Paola Yanquen, Alpina José Ortegón, Herbalife Laura Otálora, Mead Johnson Nutrition |  | <https://ilsinorandino.org/consejo-directivo/> | 14-Aug-19 |
| A115 | ILSI | Industry website | Coalition management | Internal | Miembros de la Industria Abbott Laboratories de Colombia S.A. Alimentos Polar Colombia S.A.S. Alpina Productos Alimenticios S.A. Amway Colombia Casa Luker Coca-Cola Servicios de Colombia, S.A. Compañía Agrícola S.A.S. – Monsanto Danisco Colombia Ltda./DuPont Nutrition & Health DSM Nutritional Products Colombia S.A. HLF Colombia Ltda. (HERBALIFE) Kellogg de Colombia S.A. Mead Johnson Nutrition Colombia Ltda. Meals de Colombia S.A.S. Mondelez Colombia S.A.S. Nestlé de Colombia Omnilife de Colombia S.A.S. Pepsico Alimentos Colombia LTDA. Postobón S.A. Procesadora Nacional de Alimentos CA – Pronaca Team Foods Colombia Ltda. Tonicorp Unilever Andina Colombia Ltda. |  | <https://ilsinorandino.org/miembros/> | 14-Aug-19 |
| A116 | ILSI | Conference | Information management | Amplification | ACODIN Congreso 2019 - Dia 2 Edulcorantes no caloricos en niños y embarazadas ¿cuál es la evidencia? Samuel Durán. PhD CHILE. Apoya: ILSI Nor Andino |  | https://drive.google.com/file/d/15wsLa88rSWC4t3va9O-jAgHxY3xCJBRK/view?usp=sharing | 06-Aug-19 |
| A117 | ILSI | Conference | Information management | Amplification | ACODIN Congreso 2019 - Dia 2 TALLER ILSI NOR ANDINO Rotulado nutricional Consuelo Quinche - Yuri Milena Castillo - COLOMBIA |  | <https://drive.google.com/file/d/15wsLa88rSWC4t3va9O-jAgHxY3xCJBRK/view?usp=sharing> | 06-Aug-19 |
| A118 | ILSI | Twitter | Information management | Amplification | ILSI Nor-Andino  @ILSINorAndino  25 may. La Comunicación Responsable en Ciencia y Salud, un tema fundamental para las sucursales de ILSI en Latinoamerica. |  | <https://twitter.com/ILSINorAndino/status/1132110064284852224> | 07-Aug-19 |
| A119 | ILSI | Twitter | Information management | Amplification | ILSI Nor-Andino @ILSINorAndino  Apr 11 Colección de artículos gratuitos con #investigaciones sobre la #microbiota #intestinal, el consumo de #aceite de #coco, #inocuidad de #alimentos, entre otros en la Revista #científica de ILSI Nutrition Reviews. http://ow.ly/GbBx50oqDsR |  | <https://twitter.com/ILSINorAndino/status/1116371072352493569> | 07-Aug-19 |
| A120 | ILSI | Twitter | Information management | Amplification | ILSI Nor-Andino @ILSINorAndino  Apr 1 El Centro de Investigación y Educación en Nutrición - Cienutrition con el apoyo de ILSI Nor-Andino invitan al Workshop: Beikost Alimentación Complementaria que se realizará el 27 de abril en Bogotá. |  | <https://twitter.com/ILSINorAndino/status/1112792106844782592> | 07-Aug-19 |
| A121 | ILSI | Twitter | Information management | Amplification | ILSI Nor-Andino @ILSINorAndino  Mar 29 Webinar: Importancia del consumo de lácteos en el adulto mayor, organizado por ILSI Nor-Andino y dirigido por Dra. Geraldine Altamar Canales. | More info here: https://drive.google.com/file/d/1meSPcVeqYY_FTH9WxyATRJIi2o7gtU08/view | <https://twitter.com/ILSINorAndino/status/1111634156453326848> | 07-Aug-19 |
| A122 | ILSI | Twitter | Information management | Amplification | ILSI Nor-Andino ‏@ILSINorAndino  Feb 8 Conoce el impacto de la #reformulación de #alimentos en la #ingesta de #nutrientes y la #salud en el estudio publicado por BMC Nutrition. http://ow.ly/OX2r50koZ8A | Study funded by Nestlé: https://bmcnutr.biomedcentral.com/articles/10.1186/s40795-018-0263-6#Abs1 Authors thank Dr. Zsuzsa Hutton (Nestlé Research Center, Lausanne, Switzerland) for her support in the organization of the project and for the revision of the final manuscript. Funding These findings are the results of work supported by Nestec SA. The views expressed in this paper are those of the authors, and no official endorsement by Nestec SA is intended or should be inferred. | <https://twitter.com/ILSINorAndino/status/1094008266894569472> | 07-Aug-19 |
| A123 | ILSI | Twitter | Information management | Amplification | ILSI Nor-Andino @ILSINorAndino  Jan 15 "Los #alimentosprocesados tienen un impacto significativo en la entrega de #nutrientes a las #personas. La adición de #ingredientes de #fortificación han reducido la prevalencia de #enfermedades", Roger Clemens, Profesor de USC School of Pharmacy USA. http://ow.ly/nJVJ50k8Bnk |  | <https://twitter.com/ILSINorAndino/status/1085265757364195328> | 07-Aug-19 |
| A124 | ILSI | Twitter | Information management | Amplification | ILSI Nor-Andino  @ILSINorAndino  Jan 9 ¿Sabías que es recomendado #consumir hasta tres #porciones de #lácteos entre #leche, #yogurt y #queso al día? |  | <https://twitter.com/ILSINorAndino/status/1083030821727031298> | 07-Aug-19 |
| A125 | ILSI | Twitter | Information management | Amplification | ILSI Nor-Andino ‏@ILSINorAndino  Jan 8 Si te perdiste el webinar sobre #Cereales: fuente de #energía para la #vida, lo puedes ver aquí: |  | <https://twitter.com/ILSINorAndino/status/1082721123752730624> | 07-Aug-19 |
| A126 | ILSI | Industry website | Information management | Amplification | Conferencias web Encuentre aquí todas las conferencias virtuales que los grupos de trabajo de ILSI Nor-Andino han realizado. Importancia del consumo de lácteos en el adulto mayor 1 de marzo de 2019 Dirigido por Dra. Geraldine Altamar Canales, Médico Cirujano de la Universidad Libre de Colombia, especialista en Gerencia en Servicios de Salud en la Universidad Cooperativa de Colombia y con doble especialidad en Medicina Interna y Geriatría de la Universidad de Caldas. |  | <https://ilsinorandino.org/conferencias-web/> | 14-Aug-19 |
| A127 | ILSI | Industry website | Information management | Amplification | Taller de Rotulado Nutricional para estudiantes de la Pontificia Universidad Javeriana Claudia Manzano, Directora Ejecutiva de ILSI Nor-Andino; Yuri Castillo, Profesora de la Facultad de Ciencias de la Pontificia Universidad Javeriana; Adriana Castaño, miembro académico de ILSI Nor-Andino; y Juan Carlos Farfán Presidente Regional de ILSI Nor-Andino.  Con el objetivo de seguir trabajando de la mano con la academia, el pasado lunes 11 de marzo de 2019, se realizó el Taller sobre Rotulado Nutricional para los estudiantes de 6º semestre de la carrera de Nutrición y Dietética de la Facultad de Ciencias de la Pontificia Universidad Javeriana. El Taller fue dirigido por el Presidente Regional de ILSI Nor-Andino, Juan Carlos Farfán, contó con la presencia de Adriana Castaño, Bióloga, Msc. en Gestión Ambiental y en Bioseguridad OGM y miembro académico de la sucursal; y fue coordinado por la Directora Ejecutiva de la misma, Claudia Manzano y la profesora de la asignatura del énfasis Rotulado Nutricional, Yuri Castillo, Nutricionista Msc. en Ciencia y Tecnología de Alimentos de la Universidad Nacional de Colombia. Durante el Taller, al que asistieron 23 estudiantes, se abordaron las generalidades y el rotulado nutricional del etiquetado de los alimentos empacados, así mismo se dio a conocer la herramienta de Excel para el cálculo preliminar de la Tabla de Información Nutricional elaborada por Juan Carlos Farfán. Al finalizar se resolvieron las dudas de los estudiantes respecto al tema y se destacó la importancia del papel del profesional en nutrición para la salud pública en los diferentes campos laborales. | Juan Carlos Farfán from Nestlé - https://co.linkedin.com/in/juan-carlos-farfan-perez-b14b24102  Adriana Castaño worked for Mondelez https://co.linkedin.com/in/adriana-casta%C3%B1o-hernandez-55090519  Claudia Manzano worked for Alpina https://co.linkedin.com/in/claudia-lucia-manzano-arenas-b78267149 Yuri Castillo | <https://ilsinorandino.org/2019/03/15/taller-de-rotulado-nutricional-para-estudiantes-de-la-pontificia-universidad-javeriana/> | 14-Aug-19 |
| A128 | ILSI | Industry website | Information management | Amplification | Premios a los nuevos talentos de la investigación científica y tecnológica de Colombia Harold Hernández, ganador segundo lugar; Esperanza Flórez, docente de la Universidad del Atlántico; Claudia Manzano, Directora Ejecutiva de ILSI Nor-Andino; y Rosa Angélica Ricardo, ganadora primer lugar. Con la representación de Claudia Manzano, Directora Ejecutiva de ILSI Nor-Andino, estuvimos presentes en la entrega del Premio a los nuevos talentos de la investigación científica y tecnológica de Colombia 2018, realizado el 8 de febrero del 2019. Rosa Angélica Ricardo (primer lugar) y Harold Hernández (segundo lugar) fueron los estudiantes premiados por sus trabajos de grado de la Universidad del Atlántico bajo la asesoría de la docente Esperanza Flórez. El evento contó con la presencia de Andrés Peñuela, Director General Herbalife Nutrition Colombia; Clara Lucía Valderrama, Integrante del Consejo Consultor de Dietistas y fue dirigido por José Ortegón, Gerente de Relaciones Gubernamentales, ambos de la misma empresa. |  | <https://ilsinorandino.org/2019/02/11/premios-a-los-nuevos-talentos-de-la-investigacion-cientifica-y-tecnologica-de-colombia/> | 14-Aug-19 |
| A129 | Kellogg | Conference | Information management | Amplification | ACODIN Congreso 2019 - Dia 2 Fibra dietética para la microbiota intestinal. ND. MSc Guadalupe Esquivel Flores - MÉXICO  Apoya: KELLOGS |  | https://drive.google.com/file/d/15wsLa88rSWC4t3va9O-jAgHxY3xCJBRK/view?usp=sharing | 06-Aug-19 |
| A130 | McDonald's | Twitter | Coalition management | Community | McDonald's Colombia Retweeted Aldeas Infantiles @AldeasColombia  Jun 13 ¡Estamos felices de ser #LaGranCausa de @McDonaldsCol! Hamburguesa Prosciutto Melt, al adquirirla, una parte se destinará a respaldar el proyecto de vida de los jóvenes que participan en los servicios de Aldeas Infantiles SOS. 😋🍔 |  | <https://twitter.com/AldeasColombia/status/1139329149192089600> | 07-Aug-19 |
| A131 | McDonald's | Twitter | Coalition management | Community | FeriadelLibroBogotá @FILBogota  May 6 Gracias a @McDonaldsCol y su programa Mc Lectura feliz, que promueve los libros como primer vínculo de la familia con la lectura.  Gracias por llenar de alegría y felicidad a cada niño y familia asistente a la feria. |  | <https://twitter.com/FILBogota/status/1125599895711252481> | 07-Aug-19 |
| A132 | McDonald's | Twitter | Coalition management | Community | McDonald's Colombia Retweeted FeriadelLibroBogotá @FILBogota  May 1 ¡Novedad #FILBo2019! Podrás disfrutar de McLectura Feliz en donde encontrarás más de 50 actividades gratuitas del 24 de abril al 6 de mayo.   Apoyo institucional: @McDonaldsCol | See also - well promoted by the Book Festival https://twitter.com/FILBogota/status/1122580243187998721 https://twitter.com/McDonaldsCol/status/1122227100516220936 https://twitter.com/McDonaldsCol/status/1121102976288985089 https://twitter.com/FILBogota/status/1120809679523807243 https://twitter.com/FILBogota/status/1118596435514208257 https://twitter.com/FILBogota/status/1113160439783985154 | <https://twitter.com/FILBogota/status/1123573055392026624> | 07-Aug-19 |
| A133 | McDonald's | Twitter | Coalition management | Community | Daniela @Valdedrama Follow Follow @Valdedrama More Daniela Retweeted Alejandra Crear el hábito no de la lectura, sino del gusto y la curiosidad por la misma desde temprana edad. Algo que puede agradecer una persona toda su vida. 💚 #McLecturaFelizDaniela added,  Alejandra @Tuiteretera Creo que la clave está en hacer que los niños se enamoren de la lectura y que no lo vean como una imposición del colegio. Amo que creen estos espacios tan bonitos donde ellos pueden explorar, crear y aprender. #McLecturaFeliz  Show this thread 12:50 PM - 27 Apr 2019 from Bogotá, D.C., Colombia  Daniela @Valdedrama  Apr 27 Si tienen chiquis en casa y tienen la oportunidad de llevarlos a la @FILBogota, no duden en pasar por el stand de @McDonaldsCol que está pensando especialmente para brindarle una experiencia lectora a los niños. 🌱💚 #McLecturaFeliz |  | <https://twitter.com/Valdedrama/status/1122226444636180482> | 07-Aug-19 |
| A134 | McDonald's | Industry website | Information management | Amplification | ¿Cómo alimentar a los niños de hoy? (…) La cadena de restaurantes McDonald’s, por ejemplo, ha trabajado los últimos ocho años en un menú infantil más nutritivo y equilibrado, con reducciones de sodio y azúcar y sin grasas trans; con menos de 600 calorías, es decir, menos de un tercio de las necesidades energéticas para niños entre 6 y 10 años, según lo recomendado por la Organización Mundial de la Salud. “La Cajita Feliz de McDonald’s aporta nutrientes para el crecimiento de los niños, como la proteína de buena calidad. La carne que usamos proviene solo de cortes enteros y magros, es decir, con bajo porcentaje de grasa; y no contiene aditivos ni conservantes artificiales. Tampoco agregamos grasa en su cocción, ya que la carne se cocina en sus jugos naturales. En los ‘nuggets’ usamos únicamente pechuga de pollo apanado y sazonador natural, sin aditivos artificiales”, señala Adriana Garzón, vocera de la marca en Colombia. | Other webpages: https://decidoloquecomo.com/buenos-habitos/en-el-corte-esta-la-clave-para-que-la-carne-de-cerdo-sea-saludable/ | <https://decidoloquecomo.com/alimentacion-infantil/como-alimentar-a-los-ninos-de-hoy/> | 14-Aug-19 |
| A135 | McDonald's | Industry website | Information management Discursive strategy | Amplification Frame the debate | El desafío de alimentar a los niños fuera de casa: ‘La cajita feliz’ De acuerdo con un estudio realizado por la consultora Trendsity*, seis de cada diez familias que comen en locales de comidas rápidas visitan McDonald’s. Aun así, para muchos padres es una indulgencia ocasional que tienen con sus hijos; no están seguros de que sea una comida nutritiva y balanceada para ellos. Diana Peláez, Directora Divisional de Marketing de Arcos Dorados—la franquicia de McDonald’s en 20 países de Latinoamérica—participó en “Sé lo que como”, el primer conversatorio de Semana sobre alimentación saludable, un evento realizado como parte de su iniciativa del mismo nombre. Diana explicó a los asistentes porqué la marca define y promueve el menú infantil como una opción saludable cuando las familias comen fuera de casa. |  | <https://decidoloquecomo.com/mitos-y-tendencias/el-desafio-de-alimentar-a-los-ninos-fuera-de-casa-la-cajita-feliz/> | 14-Aug-19 |
| A136 | Nestle Coca-Cola Kellogg | Civil society website | Coalition management | Community | Nuestros aliados Para Corporación Juego y Niñez el trabajo interinstitucional e intersectorial constituye la clave para alcanzar nuestro propósito primordial de empoderar a las niñas y a los niños de Colombia al fortalecer sus habilidades para la vida y su creatividad jugando. Con nuestros aliados nos sentamos y nos ponemos de acuerdo, gracias a ellos nos acercamos a los 20 años de trabajo articulado por la niñez del país. ACTORES PRIVADOS CHEVRON NESTLE PETROBRAS COCA COLA FEMSA ARGOS PEPE GANGA INSTITUCIÓN UNIVERSITARIA POLITÉCNICO GRANCOLOMBIANO KELLOG's INC PROCTER & GAMBLE |  | <https://juegoyninez.org/nuestros-aliados/> | 20-Aug-19 |
| A137 | ANDI Coca-Cola McDonald's Mars | Other | Coalition management | Internal | Concordia is a registered 501(c)(3) nonprofit, nonpartisan organization dedicated to actively fostering, elevating, and sustaining cross-sector partnerships for social impact (https://www.concordia.net/about/)  Organization (only our actors of interest): FENALCO, ANDI, Coca-Cola, McDonald's, Mars |  | <https://www.concordia.net/organization/> | 21-Aug-19 |
| A138 | Multiple | Industry website | Coalition management | Internal | UNA INICIATIVA DE: Semana, Fundacion Exito, Gourmet, Nestle PRESENTA: Zenu, Tetrapack, Doria, Levapan, Pork Colombia APOYA: ANDI, Live Life |  | <https://decidoloquecomo.com/> | 14-Aug-19 |
| A139 | Danone Exito Nutresa Unilever ANDI | Industry website | Coalition management | Community | PROGRAMA DE NUTRICIÓN Mejorar la nutrición y la salud de poblaciones vulnerables de Colombia es un compromiso de Alquería. Nuestro programa de nutrición ha beneficiado a miles de familias mediante donaciones, campañas de sensibilización y distintos frentes de trabajo para reducir la pobreza, el hambre y la desnutrición  Por ello, y desde hace más de 12 años, hemos trabajado de manera optimista con diferentes bancos de alimentos, organismos sin ánimo de lucro que centran sus esfuerzos para garantizar la alimentación de población necesitada. Comprometida a luchar contra la pobreza extrema y el hambre, en 2009 Alquería se unió con el Grupo y Fundación Éxito, el Grupo y Fundación Nutresa, Unilever y la ANDI para fundar la Asociación de Bancos de Alimentos de Colombia (ABACO), que reúne a 19 bancos de alimentos a nivel nacional. Hoy, Carlos Enrique Cavelier, Coordinador de Sueños de Alquería, es parte activa de la junta directiva de ABACO. En los últimos 5 años hemos donado más de 9.735.237 litros de leche a 15 bancos de alimentos en 12 ciudades de Colombia. FAMILIAS Y BENEFICIOS El corazón de Alquería es la familia. Por eso, y a través de distintas iniciativas como nuestro programa de Nutrición, contribuimos para mejorar la salud y la calidad de vida de los colombianos. Con el apoyo de más de 700 empresas y en alianza con distintos bancos de alimentos en todo el país, en los últimos 10 años han sido beneficiadas más de 116.000 personas y 14.000 familias. Ellos han compartido con nosotros sus testimonios y voces de esperanza. |  | <https://www.alqueria.com.co/programa-de-nutricion/#familias> | 15-Aug-19 |
| A140 |  | Government website | Direct involvement and influence in policy | Lobby | Congresso de la Republica de Colombia - Camara de Representantes - Registro Público de Cabilderos - Registro Cabilderos 2014-2018 |  | <http://www.camara.gov.co/camara/visor?doc=/sites/default/files/2017-07/Registro%20Cabilderos%20%282015-04-21%29.pdf> | 22-Aug-19 |
| A141 | Nutresa Coca-Cola | Media | Direct involvement and influence in policy | Lobby (revolving door) Incentives | o Antes de ser electo presidente de Colombia, Iván Duque Márquez fue senador entre los años 2014 y 2018. Durante su etapa como parlamentario lideró una campaña en contra del impuesto a las bebidas azucaradas y apareció en un video en el que afirmó que el azúcar no es la principal causa de la obesidad. La campaña al Senado de 2014 de Iván Duque, así como la de los otros candidatos del partido Centro Democrático de ese periodo legislativo, fue financiada por ingenios azucareros, Nutresa y Bavaria. o La empresa Econcept es una consultora económica que ha elaborado estudios para Coca-Cola, SaludCoop EPS y Asocaña. Entre sus fundadores y socios se encuentran funcionarios que han ocupado ministerios y altos cargos en el sector público. JUAN CARLOS ECHEVERRY GARZÓN MINISTERIO DE HACIENDA Y CRÉDITO PÚBLICO Ministro (2010-2012) MAURICIO SANTAMARÍA SALAMANCA ECONCEPT Representante legal (2014-2017) ANDRÉS ESCOBAR ARANGO ECONCEPT Presidente (2009-2014) |  | <https://saludconlupa.com/reportajes/la-salud-en-la-mesa-del-poder/puertas-que-giran-sin-parar/> | 22-Aug-19 |
| A142 | ANDI | Media | Direct involvement and influence in policy | Lobby (revolving door) | Santiago Durán Otero, director ejecutivo de la Cámara de la Industria de Bebidas de la ANDI en el 2018, sabe trabajar con todo tipo de público. Eligió uno muy selecto a inicios de junio de 2018, durante el debate de la "Ley de la comida chatarra", un proyecto que buscaba regular la publicidad de alimentos no saludables y ultraprocesados. En esa oportunidad se acercó personalmente a algunos senadores de la República. Según relató la Liga contra el Silencio, a Durán se le vio muy activo, de lado a lado del recinto, hablando con los congresistas que votarían el articulado que aumentaba las regulaciones para la industria. Aunque no es posible asegurar que las votaciones en contra de la llamada Ley de la comida chatarra se debieron a la intervención de Otero, su acto fue parte de una serie de jugadas a favor del sector que él representaba. (...) Luego de que el espectáculo del etiquetado llegara a su fin, Durán Otero desapareció de la ANDI y, en un abracadabra, apareció como asesor del presidente Iván Duque y como su representante en el proyecto Pazcifico. |  | <https://saludconlupa.com/reportajes/la-salud-en-la-mesa-del-poder/los-trucos-del-lobby-de-la-salud-en-colombia/> | 22-Aug-19 |
| A143 | ANDI, Postobon | Media | Direct involvement and influence in policy Discursive strategy | Lobby Frame the debate | Sí hubo presencia de gremios como la Asociación Nacional de Empresarios de Colombia (ANDI) y la Federación Nacional de Comerciantes (Fenalco); y oficinas de cabildeo como Axis, cuyo director, Felipe Campo, asistió a varias sesiones donde el proyecto de Ley de Etiquetado estuvo en el orden del día.  “En todos los debates están. En comisión sacan a los representantes, les dicen más o menos qué tienen que hacer, se acercan a los asesores y les dicen: ‘Levanten ya esta sesión’”, dijo un representante de la sociedad civil que pidió no ser identificado. “Eso lo vimos en la sesión cuando llegó Santrich; una persona de Axis le dijo al asesor de un miembro de la mesa directiva: ‘Ya no más, levanten esta sesión’. Esa era la orden”. (...) Estas dos fuentes que observaron el proceso aseguran que la industria, y las organizaciones que la apoyan, sostienen reuniones con los congresistas uno por uno, donde repiten los mismos argumentos que presentaron en el debate por el impuesto a las bebidas azucaradas. (...) El representante Mauricio Toro, dice haber visto similitudes entre el lobby contra el etiquetado, y la manera como ha operado en la comisión el lobby del tabaco y del asbesto, para impedir regulaciones. “Había una fuerza que estaba trabajando con mensajes para algunos de los ponentes del proyecto, porque cuando usted empieza a oír las razones y las motivaciones que aducen, se nota que no son construcciones propias, se nota que es información que les han entregado personas que tienen un interés en el proyecto“, dijo Toro. (...) Postobón ejerció la presión más evidente para impedir que prosperara la propuesta original del etiquetado de advertencia. Varias fuentes confirmaron que cuando arreció la presión de la sociedad civil y los medios de comunicación, representantes de la empresa se acercaron a los congresistas para entregarles un texto distinto de proyecto de ley, que conciliara las dos posiciones.  “Los congresistas nos dijeron que les había ido a buscar Postobón para poner una propuesta que bajara los ánimos de la sociedad civil, porque sabían que la presión ha estado muy fuerte. A los congresistas la industria les dijo: ‘haga una propuesta conciliadora, cree esta comisión y agárrese de que no podemos copiar simplemente porque sí el modelo de Chile’”, dijo el representante Toro.  Contactamos a Postobón para conocer su versión sobre este tema, y en comunicado dirigido a La Liga respondió: “Junto con empresas pertenecientes a las cámaras de Bebidas y de Alimentos de la ANDI, participamos en espacios como audiencias públicas, foros y reuniones, convocados no sólo por los legisladores, sino también por otros grupos de interés, en los que siempre explicamos la posición de la industria desde los puntos de vista técnico y científico”. (...) Postobón, sin embargo, dijo también en su comunicado: “Es importante resaltar que la industria de bebidas ha avanzado en el etiquetado frontal sin necesidad de que exista una obligación legal, aportando elementos de juicio para que los esfuerzos regulatorios se encaminen a informar mejor a los consumidores”. |  | <https://ligacontraelsilencio.com/2019/07/11/asi-fue-el-lobby-en-el-congreso-contra-la-ley-de-etiquetado/> | 22-Aug-19 |
| A144 | Not specified | Media | Direct involvement and influence in policy | Actor in government decision making | Las empresas más grandes de bebidas azucaradas de Colombia acordaron en 2016 no vender ciertos productos en las escuelas primarias. Tres años más tarde, siguen sin cumplir el compromiso. |  | <https://pacifista.tv/notas/empresas-de-bebidas-azucaradas-incumplen-acuerdos-de-venta-en-colegios/> | 22-Aug-19 |
| A145 | Multiple | Twitter | Discursive strategy | Frame the debate | Sé Lo Que Como a retweeté  Revista Semana Compte certifié @RevistaSemana  17 juil. En @decidoloquecomo \| Alimentarnos es uno de los actos más importantes del día. Hacerlo correctamente requiere incluir las porciones adecuadas, sin excesos, de todos los tipos de alimento de forma dosificada a lo largo del día.  #SéLoQueComo   🔗 http://bit.ly/2xPOxKI |  | <https://twitter.com/RevistaSemana/status/1151284378716315650> | 12-Aug-19 |
| A146 | Multiple | Twitter | Discursive strategy | Frame the debate | Sé Lo Que Como a retweeté Revista Semana Compte certifié @RevistaSemana  15 juil. .@decidoloquecomo \| Alimentarse de forma equilibrada no se limita a acciones con plazo definido, ni a seguir unas reglas únicas; es parte de un estilo vida saludable.   #SéLoQueComo y por eso #DecidoLoQueComo  🔗 http://bit.ly/2xPOxKI |  | <https://twitter.com/RevistaSemana/status/1150853791228416001> | 12-Aug-19 |
| A147 | Multiple | Twitter | Discursive strategy | Frame the debate | Sé Lo Que Como @decidoloquecomo  12 juil. “El secreto de comer está en el cómo se porciona esa ingesta colectiva para no recargar el cuerpo de un exceso de calorías que no va a consumir”, dice Luis Bonet, médico experto en metabolismo. 🥗🍱   #SéLoQueComo y por eso #DecidoLoQueComo |  | <https://twitter.com/decidoloquecomo/status/1149772738187452416> | 12-Aug-19 |
| A148 | Multiple | Twitter | Discursive strategy | Frame the debate | Sé Lo Que Como  @decidoloquecomo  2 avr. Actividad física: desde una perspectiva fisiológica, es cualquier movimiento corporal que de como resultado un gasto energético.   Ej: trabajar, sentarse, pararse, caminar, tocar un instrumento musical, bailar, limpiar.   ¡Así que muévete!  #SéLoQueComo y por eso #DecidoLoQueComo |  | <https://twitter.com/decidoloquecomo/status/1113112199306887168> | 12-Aug-19 |
| A149 | Multiple | Twitter | Discursive strategy | Frame the debate | Sé Lo Que Como @decidoloquecomo  6 mars Vivimos en un mundo que se transforma rápidamente. Cada día surgen desafíos que nos impulsan a cambiar costumbres e implementar nuevos hábitos que, en la mayoría de ocasiones, no corresponden a las prácticas de una nutrición responsable.  #SéLoQueComo y por eso #DecidoLoQueComo |  | <https://twitter.com/decidoloquecomo/status/1103406249327161347> | 12-Aug-19 |
| A150 | Multiple | Twitter | Discursive strategy | Frame the debate | Bebidas de tu lado @bebidasdetulado  31 juil. Plus Conoce nuestros 5 compromisos de autorregulación y descubre que estamos haciendo las bebidas para estar #DeTuLado. Visita http://www.bebidasdetulado.com | See also here: https://twitter.com/bebidasdetulado/status/1152941298007101440 https://twitter.com/bebidasdetulado/status/1151846582846808064 https://twitter.com/bebidasdetulado/status/1150563126544412672 | <https://twitter.com/bebidasdetulado/status/1156580280636989441> | 12-Aug-19 |
| A151 | Multiple | Twitter | Discursive strategy | Frame the debate | Bebidas de tu lado @bebidasdetulado  31 juil. Desde 2016 tenemos el compromiso de continuar con la #InnovaciónPositiva de nuestro portafolio para poder ofrecerte bebidas que se adapten mejor con tu estilo de vida. |  | <https://twitter.com/bebidasdetulado/status/1156361332402212869> | 12-Aug-19 |
| A152 | Multiple | Twitter | Discursive strategy | Frame the debate | Bebidas de tu lado  @bebidasdetulado  30 juil. Las bebidas queremos poner la información nutricional a tu alcance para contribuir a la toma de decisiones conscientes de consumo. Visita http://www.bebidasdetulado.com para más #InformaciónClara | See also here: https://twitter.com/bebidasdetulado/status/1152027777304092672 https://twitter.com/bebidasdetulado/status/1151287902300622849 | <https://twitter.com/bebidasdetulado/status/1156195238110736384> | 12-Aug-19 |
| A153 | Multiple | Twitter | Discursive strategy | Frame the debate | Bebidas de tu lado @bebidasdetulado  28 juil. Nuestros consumidores merecen #InformaciónClara y eso es lo que queremos darles. Por eso hace más de 1 año firmamos un compromiso de autorregulación para cambiar nuestras etiquetas. ¡Las bebidas estamos #DeTuLado! |  | <https://twitter.com/bebidasdetulado/status/1155530860550115328> | 12-Aug-19 |
| A154 | Multiple | Twitter | Discursive strategy | Frame the debate | Bebidas de tu lado @bebidasdetulado  28 juil. ¿Quieres saber qué hace la industria de las bebidas para promover una #VidaBalanceada? Trabajamos con @tudiscovery y #HealthyWeightc en #JuntosContamos. Más información en este link --> http://co.juntoscontamos.com |  | <https://twitter.com/bebidasdetulado/status/1155274168176267265> | 12-Aug-19 |
| A155 | Multiple | Twitter | Discursive strategy | Frame the debate | Bebidas de tu lado @bebidasdetulado Respetamos la autonomía de los padres de familia, por eso hacemos una #PublicidadConsciente. Visita http://www.bebidasdetulado.com para saber por qué estamos #DeTuLado.  07:00 - 26 juil. 2019 | See also: https://twitter.com/bebidasdetulado/status/1150200738079027201 | <https://twitter.com/bebidasdetulado/status/1154753238459133952> | 12-Aug-19 |
| A156 | Multiple | Twitter | Discursive strategy | Frame the debate | Bebidas de tu lado @bebidasdetulado Suivre Suivre @bebidasdetulado Las decisiones nutricionales de los niños deben ser tomadas por sus padres. Visita http://www.bebidasdetulado.com para más información sobre la #PublicidadConsciente.  17:00 - 25 juil. 2019 |  | <https://twitter.com/bebidasdetulado/status/1154541844044353542> | 12-Aug-19 |
| A157 | Multiple | Twitter | Discursive strategy | Frame the debate | Bebidas de tu lado @bebidasdetulado  16 juil. Tenemos más opciones para ti. Aprende más sobre la #InnovaciónPositiva que estamos haciendo en nuestro portafolio. http://www.bebidasdetulado.com | See also: https://twitter.com/bebidasdetulado/status/1150759419434524674 https://twitter.com/bebidasdetulado/status/1150464979570741249 | <https://twitter.com/bebidasdetulado/status/1151129362097422337> | 12-Aug-19 |
| A158 | Multiple | Twitter | Discursive strategy | Frame the debate | Bebidas de tu lado ‏@bebidasdetulado  16 juil. #JuntosContamos enseña a niños y a sus familias a llevar una dieta adecuada y una #VidaBalanceada. Visita http://co.juntoscontamos.com para más información. |  | <https://twitter.com/bebidasdetulado/status/1150925514565025792> | 12-Aug-19 |
| A159 | Multiple | Twitter | Discursive strategy | Frame the debate | Bebidas de tu lado @bebidasdetulado  14 juil. Las bebidas te damos las herramientas para que puedas tomar con plena autonomía las decisiones nutricionales de tus hijos. Estamos #DeTuLado. Visita http://www.bebidasdetulado.com |  | <https://twitter.com/bebidasdetulado/status/1150389484199874560> | 12-Aug-19 |
| A160 | ILSI Unilever | Conference | Information management | Amplification | Nuestros Patrocinadores ACODIN no tiene conflicto de intereses con empresas, organizaciones o la industria frente a su información y productos. La información de conferencistas y sus posturas de apoyo son responsabilidad exclusiva de cada una de ellas. ACODIN es una entidad que no excluye y que escucha todas las posturas de los actores institucionales, no institucionales, académicos y civiles como una medida de paz y respeto para los que conformamos una sociedad.   Categoría Oro Boydorr  Categoría Estándart Pietran Laboratorio Laproff FENAVI Celan **ILSI** Gatorade LATAM Ookio Fedepalma eBiody Abbott MenusPlus **Naatu (Unilever)** Cafe de Colombia Efigas Alanur IdeoLifes Instituto de Nutricion y Salud Kellog's CPDCaldas Taeq Fresenius Kabi Ron Viejo de Caldas Industria Licorera de Caldas Universidad Catolica de Manizales |  | <https://acodin.org/congreso-2019/> | 06-Aug-19 |
| A161 | Unilever | Conference | Information management | Amplification | ACODIN Congreso 2019 - Dia 3 SIMPOSIO (22) TEMÁTICAS SELECTAS EN NUTRICIÓN 5  MODERADOR: Ana María Castaño Ramírez Nd Msc ICBF Caldas **8:00 - 8:45 Los cereales, una opción en el desarrollo de alimentos, nutritivos, económico y viable. Sara Valdes. Presidente ALACCTA. MEXICO** 8:45 - 9:15 **TAEQ** 9:15 - 10:00 Comida del Futuro: dietas sostenibles para personas y planeta sanos. Fernanda de Oliveira Martins. ND Msc. BRASIL  **Apoya: Unilever** |  | https://drive.google.com/file/d/1Nqc5zkLrMZoSWw6zKfGSSX7JHNoXfRRH/view | 06-Aug-19 |
| A162 | Danone ILSI McDonald's | Conference | Information management | Amplification | 33° Congreso de Nutrición Clínica y Metabolismo y 4° Congreso FELANPE Regional Andino Patrocinadores: Nutricia (Danone), ILSI, FENAVI, Fedepalma, Cenipalma, Arcos Dorados, Abbott, etc  Viernes 03 Mayo: 10:10 - 11:40: Nutrición Infantil -ILSI - ACNC - Liliana Ladino ND, Col |  | https://www.nutriclinicacolombia.org/congreso/ https://www.nutriclinicacolombia.org/wp-content/uploads/2019/04/programa-congreso-2019-F2.pdf | 06-Aug-19 |
| A163 | Multiple | Twitter | Information management | Amplification | Sé Lo Que Como a retweeté  Revista Semana Compte certifié @RevistaSemana  23 juil. En @decidoloquecomo \| Los cereales son los que le darán a su hijo la energía necesaria para estudiar, correr y jugar. Las proteínas contribuyen a la fabricación de nuevos tejidos, a la elaboración de enzimas y al aumento de masa muscular.  #SéLoQueComo  🔗 http://bit.ly/2StHzET |  | <https://twitter.com/RevistaSemana/status/1153760443585798144> | 12-Aug-19 |
| A164 | Multiple | Twitter | Information management | Amplification | Bebidas de tu lado @bebidasdetulado  17 mai Las personas no tenemos las mismas necesidades nutricionales. Los etiquetados de advertencia no ofrecen #InformaciónClara sobre el aporte energético y nutricional de cada bebida. |  | <https://twitter.com/bebidasdetulado/status/1129386088764715010> | 12-Aug-19 |
| A165 | Multiple | Twitter | Information management | Amplification | Bebidas de tu lado ‏@bebidasdetulado  16 mai El etiquetado GDA aporta información confiable, distinto a lo que un etiquetado de advertencia puede hacer. #InformaciónClara | See also: https://twitter.com/bebidasdetulado/status/1128751908099747842 | <https://twitter.com/bebidasdetulado/status/1129114296674209792> | 12-Aug-19 |
| A166 | Multiple | Twitter | Information management | Amplification Actor in government | En la Comisión Séptima de @SenadoGovCo se presentaron los argumentos de Susana Socolovsky, Ph. D., sobre #EtiquetadoFrontal como representante de la academia. Socolovsky tiene una amplia experiencia en estos temas y cuenta con el reconocimiento internacional de sus pares. 1:16  Bebidas de tu lado ‏@bebidasdetulado  15 mai Susana Socolovsky, Ph. D., nos explica de forma precisa qué es el #EtiquetadoFrontal de acuerdo con lo que determina el Codex Alimentarius @FAOWHOCodex. #InformaciónClara 1:33  Bebidas de tu lado ‏@bebidasdetulado  15 mai La discusión en Colombia sobre el #EtiquetadoFrontal coincide con la reunión del Codex Alimentarius @FAOWHOCodex en #Canadá en la que se trata el mismo tema. #InformaciónClara 0:40  Bebidas de tu lado ‏@bebidasdetulado  15 mai Susana Socolovsky, Ph. D., nos cuenta que en el mundo únicamente cuatro países tienen esquemas de #EtiquetadoFrontal obligatorios. Esto es debido a que el Codex Alimentarius @FAOWHOCodex no cuenta con una directriz al respecto. #InformaciónClara 0:59  Bebidas de tu lado ‏@bebidasdetulado  15 mai Susana Socolovsky, Ph. D., nos explica qué es Codex Alimentarius @FAOWHOCodex y por qué es importante saberlo para discutir con propiedad los temas de #EtiquetadoFrontal. Si quieres tener más información al respecto, visita --> http://www.fao.org/fao-who-codexalimentarius/es/ …  Bebidas de tu lado ‏@bebidasdetulado  15 mai ¿Por qué es importante y para qué sirve el #EtiquetadoFrontal? Aquí lo fundamental es educar al consumidor. #InformaciónClara 2:20  Bebidas de tu lado ‏@bebidasdetulado Existe un mito que afirma que la población no aprende a leer el #EtiquetadoGDA. Susana Socolovsky, Ph. D., nos aclara que no existe sustento científico para tal afirmación y que hay países que sí lograron educar a los consumidores. #InformaciónClara 1:07 15:30 - 15 mai 2019 |  | https://twitter.com/bebidasdetulado/status/1128789861576708096 | 12-Aug-19 |
| A167 | Multiple | Twitter | Information management | Amplification | Bebidas de tu lado ‏@bebidasdetulado  14 mai El etiquetado GDA cuenta con respaldo científico y está basado en las guías aprobadas por las autoridades internacionales. #InformaciónClara | Many more Tweet about this in mid May - criticism of warning labels and promotion of GDA | <https://twitter.com/bebidasdetulado/status/1128314021558530052> | 12-Aug-19 |
| A168 | Multiple | Twitter | Information management Direct involvement and influence in policy | Amplification Actor in government decision making | En la Comisión Séptima de @CamaraColombia, Susana Socolovsky, Ph.D., hizo una intervención sobre el etiquetado frontal en nombre de la ciencia de los alimentos que en sus palabras no había estado representada en dicha audiencia. #InformaciónClara  ANDI  Bebidas de tu lado @bebidasdetulado  9 mai "El arte de etiquetar alimentos forma parte de una de las premisas de la @WHO que vela por la salud de los consumidores en el mundo entero": Susana Socolovsky, Ph.D. #InformaciónClara  Bebidas de tu lado ‏@bebidasdetulado  9 mai "En 1963, la @WHO y la @FAO convirtieron a un grupo de personas interesadas en la salud de la población en una entidad que se llama Codex Alimentarius": Susana Socolovsky, Ph.D. #InformaciónClara Si quieres saber qué es el Codex Alimentarius 🍉🍏🥤🥛--> http://www.fao.org/fao-who-codexalimentarius/home/es/ …  Bebidas de tu lado ‏@bebidasdetulado  9 mai El Codex Alimentarius tiene dos misiones fundamentales: velar por la salud poblacional global y garantizar las prácticas equitativas de comercio. Esta entidad tratará el tema del etiquetado frontal la próxima semana: Susana Socolovsky, Ph.D. #InformaciónClara  Bebidas de tu lado @bebidasdetulado  9 mai El tema del etiquetado frontal constituye un tema de primordial importancia para el mundo entero: Susana Socolovsky, Ph.D. #InformaciónClara  ANDI  Bebidas de tu lado @bebidasdetulado  9 mai La tabla nutricional es una creación del Codex Alimentarius y como tal ha sido armonizada por la intervención de todos los países. En el Codex se sienta el 99% de los países del mundo con voz y voto: Susana Socolovsky, Ph.D. #InformaciónClara  Bebidas de tu lado @bebidasdetulado  9 mai El Codex Alimentarius regula para el mundo entero y constituye la base normativa de todos los países. Permítanme decirles que también para el @invimacolombia el Codex es la base normativa: Susana Socolovsky, Ph.D. #InformaciónClara  Bebidas de tu lado @bebidasdetulado  9 mai Y la norma colombiana dice que en ausencia de norma, la norma es el Codex: Susana Socolovsky, Ph.D. #InformaciónClara  Bebidas de tu lado @bebidasdetulado  9 mai Partiendo de esto es importante resaltar dos cosas: 1. El Proyecto de Ley 214, que está cursando en esta Cámara, no es el proyecto de Chile pues tiene solamente uno de los elementos del etiquetado chileno que es la señal de advertencia: Susana Socolovsky, Ph.D. #InformaciónClara  Bebidas de tu lado @bebidasdetulado 2. Si leen el proyecto verán que usa definiciones de alimentos a los que llama mínimamente procesados, ingredientes primarios, alimentos procesados y ultraprocesados, definiciones que no están aceptadas por la normatividad internacional: Susana Socolovsky, Ph.D. #InformaciónClara 16:21 - 9 mai 2019 |  | <https://twitter.com/bebidasdetulado/status/1126628184965963776> | 12-Aug-19 |
| A169 | Multiple | Twitter | Information management Direct involvement and influence in policy | Amplification Actor in government decision making | En este momento inicia su intervención en la Comisión Séptima de @CamaraColombia el director ejecutivo de la Cámara de la Industria de Bebidas, @l_felipetorres. Las bebidas creemos en la #InformaciónClara.   ANDI Bebidas de tu lado ‏@bebidasdetulado  9 mai "Todos estamos de acuerdo con que el etiquetado actual, es decir, la tabla nutricional no es suficiente información para el consumidor. Lo que estamos buscando aquí es identificar el mejor modelo de etiquetado": @l_felipetorres #InformaciónClara  Bebidas de tu lado ‏@bebidasdetulado  9 mai "Este proyecto [sobre etiquetado] debe buscar qué es lo mejor para el consumidor colombiano": @l_felipetorres #InformaciónClara  Bebidas de tu lado ‏@bebidasdetulado  9 mai Es un compromiso de Colombia adoptar estudios de impacto normativo donde se haga un análisis de costo-beneficio al momento de expedir una medida regulatoria: @l_felipetorres #InformaciónClara   ANDI Bebidas de tu lado ‏@bebidasdetulado  9 mai Vemos como industria [de bebidas] que el debate actual de etiquetado carece de este análisis de costo-beneficio lo cual le impide al país avanzar en una política pública basada en evidencia científica: @l_felipetorres #InformaciónClara  Bebidas de tu lado ‏@bebidasdetulado  9 mai El etiquetado informativo, que utilizan la mayoría de países europeos y los Estados Unidos, se conoce como GDA. Éste se basa en las guías aprobadas por la @WHO, en las recomendaciones de la autoridad sanitaria de la Unión Europea y la FDA: @l_felipetorres #InformaciónClara  Bebidas de tu lado ‏@bebidasdetulado  9 mai El etiquetado GDA responde a los lineamientos de la normativa del Codex Alimentarius: @l_felipetorres #InformaciónClara  (¿Quieres conocer qué es el Codex Alimentarius🥚🥛🥤🍉🍍? Visita esta página web de la @FAO --> http://www.fao.org/fao-who-codexalimentarius/es/ …)  Bebidas de tu lado ‏@bebidasdetulado 186 países del mundo hacen parte del Codex Alimentarius y los lineamientos del etiquetado GDA hacen parte de sus recomendaciones: @l_felipetorres #InformaciónClara  09:59 - 9 mai 2019  Bebidas de tu lado ‏@bebidasdetulado  9 mai El etiquetado GDA es claro, sencillo y comprensible porque indica de manera exacta el aporte de las energías calóricas y de nutrientes (grasas, azúcares y sodio) : @l_felipetorres #InformaciónClara  Bebidas de tu lado ‏@bebidasdetulado  9 mai A diferencia de otras opciones el etiquetado GDA no discrimina y sí ayuda a educar a los consumidores sobre el aporte nutricional de cada alimento: @l_felipetorres #InformaciónClara  Bebidas de tu lado ‏@bebidasdetulado  9 mai Desde la @ANDI_Colombia y con las empresas que hacen parte de la Cámara de la Industria de Bebidas, desde 2016 hemos adoptado el etiquetado GDA como un mecanismo de autorregulación [pues no lo exige la ley]: @l_felipetorres #InformaciónClara  Bebidas de tu lado ‏@bebidasdetulado  9 mai Existen otros esquemas de etiquetado como los de advertencia. Nosotros los consideramos altamente cuestionados pues crean alertas en los consumidores a través de un juicio subjetivo sobre la idoneidad de un producto: @l_felipetorres #InformaciónClara |  | <https://twitter.com/bebidasdetulado/status/1126532123857387521> | 12-Aug-19 |
| A170 | Multiple | Twitter | Information management Discursive strategy | Amplification Frame the debate | Bebidas de tu lado @bebidasdetulado  28 juil. La @WHO recomienda que una persona adulta con un peso saludable consuma 2.000 calorías al día. Nosotros te damos #InformaciónClara basándonos en ese número de calorías. #DeTuLado | Present GDA in the picture See also: https://twitter.com/bebidasdetulado/status/1155168472852078592 https://twitter.com/bebidasdetulado/status/1152631757201530881 | <https://twitter.com/bebidasdetulado/status/1155485560821047296> | 12-Aug-19 |
| A171 | Multiple | Twitter | Information management Discursive strategy | Amplification Frame the debate | Bebidas de tu lado  @bebidasdetulado  23 juil. La #InformaciónClara que te presentamos en nuestras bebidas incluye las calorías y los cuatro nutrientes reconocidos como importantes desde el punto de vista de salud pública: grasa total, grasa saturada, azúcares y sodio. #DeTuLado |  | <https://twitter.com/bebidasdetulado/status/1153809517316059136> | 12-Aug-19 |
| A172 | Multiple | Twitter | Information management Discursive strategy | Amplification Frame the debate | Bebidas de tu lado @bebidasdetulado  23 juil. Aprende y enseña a otros a leer el etiquetado frontal de las bebidas. Así, con #InformaciónClara, llevar una dieta balanceada será más fácil. #DeTuLado http://www.bebidasdetulado.com |  | <https://twitter.com/bebidasdetulado/status/1153658523550720001> | 12-Aug-19 |
| A173 | Multiple | Twitter | Information management Discursive strategy | Amplification Frame the debate | Bebidas de tu lado @bebidasdetulado  23 juil. #JuntosContamos es un proyecto que busca el bienestar de la comunidad y la promoción de los buenos hábitos de vida. ¡Las bebidas estamos #DeTuLado! Visita http://co.juntoscontamos.com |  | <https://twitter.com/bebidasdetulado/status/1153462229624328194> | 12-Aug-19 |
| A174 | Multiple | Twitter | Information management Discursive strategy | Amplification Frame the debate | Bebidas de tu lado  @bebidasdetulado  16 mai No hay información científica que demuestre que el etiquetado de advertencia ayuda a disminuir los índices de obesidad. #InformaciónClara |  | <https://twitter.com/bebidasdetulado/status/1129023702132187143> | 12-Aug-19 |
| A175 | Multiple | Industry website | Information management Discursive strategy | Amplification Frame the debate | Por eso se hace tan necesario modificar los hábitos alimenticios sin necesidad de satanizar la comida y teniendo en cuenta las características y rutinas de cada persona. De acuerdo con el nutriólogo Benjamín Ramírez, “no hay alimentos que aumenten o disminuyan de peso. Hay unos que tienen densidades energéticas altas y densidades energéticas bajas, pero el problema no es el aporte calórico sino cómo se comporta este aporte energético con la parte hormonal de cada individuo”. |  | <https://decidoloquecomo.com/el-secreto-de-comer/hay-alimentos-buenos-o-malos/> | 14-Aug-19 |
| A176 | Nestle | Twitter | Coalition management | Community | Nestlé Colombia a retweeté  Ashoka Colombia @Ashoka_Colombia  30 juil. Buscamos jóvenes que puedan crear y desarrollar proyectos de emprendimiento que impacten de manera positiva en la sociedad y el medio ambiente. Si eres uno de ellos, ¡postula a …http://www.iniciativaporlosjovenes-comprometidos.org ! Tu idea   Organizan: @AshokaAr @SocialabUY @Unesco_es Apoya: @NestleColombia |  | <https://twitter.com/Ashoka_Colombia/status/1156232985680572416> | 09-Aug-19 |
| A177 | Nestle | Industry website | Coalition management | Health organisations and government bodies Internal | Nuestros Aliados ALIADOS Unidos por Niños Saludables es un verdadero esfuerzo de equipo. Está conformado por organizaciones con ideas afines, padres, creativos, científicos y académicos. Es un grupo diverso que trabaja unido para lograr nuestro único objetivo: ayudar a los padres a educar hijos felices y saludables. Nestlé “La razón que nos convoca son nuestros hijos, a quienes queremos llegar a través de sus padres y educadores y en quienes queremos promover hábitos saludables para que tengan un futuro más sano” Ogilvy Como agencia creativa, estamos comprometidos con los niños y creemos que la diversión es el camino para sembrar en ellos y en sus padres, hábitos más saludables. Mind Share “Estamos comprometidos con ayudar a difundir hábitos saludables en los niños. Queremos que cada vez más personas conozcan la iniciativa y se unan por un futuro más Feliz.” SCP “El Bienestar de los niños y adolescentes en el país es nuestra razón de ser por esto es importante enseñarles hábitos saludables para que crezcan sanos y Fuertes.” Mi Señal Señal Colombia es un canal de televisión de carácter público, que ofrece contenido educativo, deportivo y cultural. A través de Mi Señal podemos dar a conocer y promover estos hábitos saludables a muchas personas en Colombia. Discovery La red Discovery a través de Discovery Kids y su programa Discovery en la Escuela tiene una gran audiencia cautiva de padres, maestros y niños. La iniciativa “Unidos por Niños Saludables” contará con el apoyo de esta red para llevar este mensaje de hábitos saludables a muchas más personas. "Estamos muy orgullosos de ser parte de esta iniciativa". FCC La Fundación Colombiana del Corazón se une a la iniciativa “Unidos por Niños Saludables”. La cual busca promover el aprendizaje temprano de hábitos saludables de nutrición y actividad física en los niños Colombianos. "Es en los niños que debemos sembrar la cultura del cuidado". Juego y Niñez Creemos que promover hábitos saludables en los niños, los ayuda a tener un mejor fututo y esa es la razón por la cual hacemos parte de esta iniciativa. | Looks like an independent charity but created by Nestle | <https://www.unidosporninossaludables.com.co/aliados> | 19-Aug-19 |
| A178 | Nestle | Twitter | Coalition management Discursive strategy | Community Frame the debate | Nestlé Colombia Retweeted Corp. Juego y Niñez @juegoyninez  Jun 24 ¡14.000 niñas y niños, 500 docentes y 3.700 padres de familia! Serán beneficiados por la iniciativa @UnidosxNinosCol de @NestleColombia.  Conoce más aquí >> https://bit.ly/2rrFCdd  #PorNiñosSaludablesYO👧👦🍉🥬 |  | <https://twitter.com/juegoyninez/status/1143210739408265218> | 09-Aug-19 |
| A179 | Nestle | Twitter | Coalition management Information management | Community Amplification | Unidos Colombia @UnidosxNinosCol  Mar 14 Iniciamos intervención con la Corporación Juego y Niñez en 12 Colegios distribuidos en las ciudades de Neiva, Valledupar y Sabana Centro, llegando aproximadamente a 4.100 Niños y Niñas con nuestra iniciativa Unidos por Niños Saludables. |  | <https://twitter.com/UnidosxNinosCol/status/1106312446891958278> | 09-Aug-19 |
| A180 | Nestle | Twitter | Coalition management Information management | Health and other organisations? Amplification | Unidos Colombia @UnidosxNinosCol  May 30 More Hoy estuvimos en la Primera Asamblea de Aliados 2019 en compañía de @Discovery_Co, @unisabana, @juegoyninez, @Educacionbogota, @Ogilvy, @SocPediatria y @MindshareCol en el que revisamos el plan de desarrollo y los tres ejes foco para el 2019. | See also: https://twitter.com/juegoyninez/status/1134135209371295744 | <https://twitter.com/UnidosxNinosCol/status/1134138017583685632> | 09-Aug-19 |
| A181 | Nestle | Twitter | Coalition management Information management | Health and other organisations? Amplification | Unidos Colombia Retweeted Corp. Juego y Niñez @juegoyninez  Jul 25 More ¡Asistencia técnica en #Neiva con @UnidosxNinosCol de @NestleColombia! Un encuentro de #Juego con 22 docentes y 2 coordinadores de la Institución José Eustacio Rivera, donde compartimos conocimientos para sembrar en la niñez hábitos saludables. #PorNiñosSaludablesYO🍎🥑 |  | <https://twitter.com/juegoyninez/status/1154425380100562944> | 09-Aug-19 |
| A182 | Nestle | Twitter | Discursive strategy | Frame the debate | Nestlé Colombia Verified account @NestleColombia  Jul 12 En Nestlé estamos trabajando para disminuir el azúcar en nuestros alimentos sin afectar su sabor. Conoce cómo lo estamos haciendo aquí. |  | <https://twitter.com/NestleColombia/status/1149695619264417792> | 09-Aug-19 |
| A183 | Nestle | Twitter | Discursive strategy | Frame the debate | Nestlé Colombia Verified account @NestleColombia  Jul 8 Así celebró @milocolombia sus 75 años con el #DíaDelDesayuno en Medellín ¡Un día de actividad física en familia! | See pictures : branding, marketing | <https://twitter.com/NestleColombia/status/1148255494387175425> | 09-Aug-19 |
| A184 | Nestle | Twitter | Discursive strategy | Frame the debate | Nestlé Colombia Verified account @NestleColombia  Jun 26 More Conoce cómo Nestlé® está contribuyendo día a día a un futuro más prometedor para las nuevas generaciones. Aquí podrás encontrar más detalles sobre las porciones de verduras que le estamos añadiendo a nuestros productos http://bit.ly/2WjPlGx |  | <https://twitter.com/NestleColombia/status/1143957508689735682> | 09-Aug-19 |
| A185 | Nestle | Twitter | Discursive strategy | Frame the debate | Nestlé Colombia Retweeted Corp. Juego y Niñez  @juegoyninez  Jun 21 ¡Aprendiendo y jugando con 15 pedagogos y nutricionistas de la iniciativa @UnidosxNinosCol de @NestleColombia! Los equipos del proyecto fortalecieron esta semana, los principales hábitos saludables que se deben promover en las niñas y niños. #PorNiñosSaludables | See pictures : branding, marketing | <https://twitter.com/juegoyninez/status/1142137140576432133> | 09-Aug-19 |
| A186 | Nestle | Twitter | Discursive strategy | Frame the debate | Nestlé Colombia Verified account @NestleColombia  Jun 6 Nestlé® ha contribuido en mejorar la calidad de vida y en asegurar un futuro más saludable para los niños. Descubre cómo lo hemos logrado llegando a las vidas de más de 50 millones de niños a nivel mundial. Conoce más en [Video] |  | <https://twitter.com/NestleColombia/status/1136722206942224385> | 09-Aug-19 |
| A187 | Nestle | Industry website | Discursive strategy | Frame the debate | Nuestras ambiciones Hemos definido tres ambiciones globales para el 2030, que guían nuestro trabajo y apoya el logro de los objetivos de desarrollo sostenible de la ONU. Ayudar a 50 millones de niños a tener vidas más saludables Ayudar a mejorar 30 millones de hogares en las comunidades directamente relacionadas con nuestras actividades empresariales Luchar para alcanzar el impacto ambiental cero en nuestras operaciones |  | <https://www.corporativa.nestle.com.co/conocenos> | 19-Aug-19 |
| A188 | Nestle | Media | Information management | Production | Durante 2018, más de 1.000 pequeños de siete colegios de la región Sabana Centro, hicieron parte del estudio “Validación de la estrategia de promoción de la salud Unidos por Niños Saludables” desarrollado por investigadores de la facultad de Enfermería y Rehabilitación de la Universidad de la Sabana, que evidenció cómo, por ejemplo, el 75,8% de los menores ahora toma más agua. (...) Vale la pena resaltar que a lo largo de la Región Sabana Centro fueron desplegadas actividades orientadas a mejorar las prácticas de familias, profesores y niños sobre la hidratación, la elección de alimentos saludables, comer y cocinar en familia, la actividad física y el manejo de las porciones. La iniciativa incluyó la instalación de bebederos en los colegios, la celebración del día del Chef; y el fortalecimiento de una red de aliados a favor de niños(as) saludables en Colombia y el mundo.  “El impacto positivo que estamos logrando y nuestra ambición de convertir esta iniciativa de educación en política pública, nos motiva a seguir realizando esfuerzos, pues más allá de los resultados, desde Nestlé le apostamos al trabajo conjunto con instituciones educativas y socios estratégicos como la Corporación Juego y Niñez o la secretaría de educación, en una alianza que nos ha permitido generar cambios tangibles en la adopción de estilos de vida saludables” afirma Martha Arbeláez, directora de Marketing y Comunicaciones de Nestlé. |  | <https://www.elnuevosiglo.com.co/articulos/05-2019-ninos-estan-cambiando-habitos-de-hidratacion-por-agua> | 22-Aug-19 |
| A189 | Nestle | Twitter | Information management Discursive strategy | Amplification Frame the debate | Nestlé Colombia Compte certifié @NestleColombia  31 juil. ¿Cómo alimentar a los niños de hoy? La respuesta está en volver a las bases de la alimentación. #DecidoLoQueComo @decidoloquecomo http://bit.ly/2yslzRi |  | <https://twitter.com/NestleColombia/status/1156599148839428103> | 09-Aug-19 |
| A190 | Nestle | Twitter | Information management | Amplification | Nestlé Colombia Compte certifié @NestleColombia  30 juil. ¿Se valen las harinas en las noches? Desde nuestra Iniciativa de Nestlé Contigo y @decidoloquecomo, te contamos cómo los carbohidratos deben estar presentes diariamente en la alimentación. http://bit.ly/2YcF6Vw #DecidoLoQueComo |  | <https://twitter.com/NestleColombia/status/1156344973710757888> | 09-Aug-19 |
| A191 | Nestle | Twitter | Information management | Amplification | Nestlé Colombia Compte certifié @NestleColombia  29 juil. Elegir, combinar, porcionar y disfrutar. Claudia Cortés, gerente de nutrición, salud y bienestar de Nestlé Colombia, recuerda hábitos que aportan a esa búsqueda del equilibrio al comer. Conoce más aquí http://bit.ly/2GAE6j0 #DecidoLoQueComo |  | <https://twitter.com/NestleColombia/status/1155957889732710403> | 09-Aug-19 |
| A192 | Nestle | Twitter | Information management | Amplification | Nestlé Colombia a retweeté  Revista Semana Compte certifié @RevistaSemana  24 juil. En @decidoloquecomo encuentra todo lo que necesita saber sobre alimentación: hábitos, mitos, tendencias, alimentar a las siguientes generaciones, entre otros.   Una iniciativa de @RevistaSemana @NestleColombia @Grupo_Exito y Grupo Team  🔗 http://bit.ly/30TPTjY |  | <https://twitter.com/RevistaSemana/status/1154002035580162054> | 09-Aug-19 |
| A193 | Nestle | Twitter | Information management | Amplification | Nestlé Colombia Verified account @NestleColombia  Mar 31 ¡Gracias a todos los que hicieron parte del #DíaDelDesayuno! Compártenos tu experiencia en el gran evento con #DíaDelDesayuno | See pictures for amplification + branding, marketing | <https://twitter.com/NestleColombia/status/1112397648697458691> | 09-Aug-19 |
| A194 | Nestle | Twitter | Information management | Amplification | Nestlé Colombia Retweeted  MILO Colombia Verified account @milocolombia  Mar 31 Acércate a nuestra Estación de Nutrición en el #DíaDelDesayunoMILO |  | <https://twitter.com/milocolombia/status/1112340434255298560> | 09-Aug-19 |
| A195 | Nestle | Twitter | Information management | Amplification | Unidos Colombia Retweeted Corp. Juego y Niñez @juegoyninez  Apr 24 "Yo vengo de #Córdoba y hoy aprendí que el plato debe ser saludable y variado, me fascinó cocinar en familia", dijo Samuel, uno de los 19 invitados de @UnidosxNinosCol en el marco de la celebración del #DíaDeLaNiñez. #Gracias @NestleColombia  #SúmateJugando |  | <https://twitter.com/juegoyninez/status/1121151142849908736> | 09-Aug-19 |
| A196 | Nestle | Industry website | Information management | Amplification | ¿Cómo leer una etiqueta nutricional?  Las tablas nutricionales describen los nutrientes en porcentajes, peso y porciones; datos que no necesariamente sabemos cómo interpretar o simplemente no entendemos. A través de su iniciativa, ‘Nestlé Contigo’, la compañía Nestlé ofrece esta guía de cinco pasos para leer una etiqueta. |  | <https://decidoloquecomo.com/el-secreto-de-comer/como-leer-una-etiqueta-nutricional/> | 14-Aug-19 |
| A197 | Nestle | Industry website | Information management | Amplification | Los hábitos más saludables Como el corazón de Unidos por Niños Saludables en Colombia, hemos establecido junto con nutricionistas y expertos en ciencias del comportamiento, cinco hábitos saludables: Maneja las porciones  Escoge variedad y nutrición  Comer y cocinar en familia  Toma más agua  Muévete más | The website and programme is built around these topics See specific webpages for more information about each topic | <https://www.unidosporninossaludables.com.co/acerca> | 19-Aug-19 |
| A198 | Nestle | Twitter | Information management Discursive strategy | Amplification Frame the debate | Nestlé Colombia Verified account @NestleColombia  May 28 Hoy en el #DíaMundialDeLaNutrición, Claudia Cortés, Gerente de Nutrición, Salud y Bienestar de Nestlé, nos cuenta que "..las porciones son la clave para el balance..." en tus comidas favoritas. #LaNutriciónEnTuIdioma | See also: https://twitter.com/NestleColombia/status/1133522087837020160 https://twitter.com/NestleColombia/status/1133462657309270017 https://twitter.com/NestleColombia/status/1133410837400567808 | <https://twitter.com/NestleColombia/status/1133547883310010368> | 09-Aug-19 |
| A199 | Nestle | Twitter | Information management Discursive strategy | Amplification Frame the debate | Nestlé Colombia ‏ Verified account @NestleColombia  May 3 Estamos comprometidos con @UnidosxNinosCol en promover hábitos de vida saludable en los niños. Conoce más sobre la iniciativa y algunos de los resultados obtenidos en una de las regiones donde opera en http://bit.ly/2IWJS1f | See Twitter @UnidosxNinosCol from the company for more information and Tweets | <https://twitter.com/NestleColombia/status/1124451046238060545> | 09-Aug-19 |
| A200 | Nestle | Twitter | Information management Discursive strategy | Amplification Frame the debate | Nestlé Colombia ‏ Verified account   @NestleColombia Follow Follow @NestleColombia More Hacer actividad física trae múltiples beneficios. Te esperamos hoy en la carrera de @milocolombia en su primer edición del #DíaDelDesayuno.  6:26 AM - 31 Mar 2019 |  | <https://twitter.com/NestleColombia/status/1112345468024033280> | 09-Aug-19 |
| A201 | Nestle | Twitter | Information management Discursive strategy | Amplification Frame the debate | Unidos Colombia ‏@UnidosxNinosCol  May 2 Hoy estamos en el Colegio IED La Fuente con 35 niños en compañía de sus familias celebrando el #DiaDelNiño con un delicioso taller de Comer y Cocinar en familia, enfocado en nuestros 5 hábitos de alimentación saludable. | See also: https://twitter.com/juegoyninez/status/1125439831759134722 https://twitter.com/juegoyninez/status/1126871798556110848 https://twitter.com/juegoyninez/status/1130473249224183811 | <https://twitter.com/UnidosxNinosCol/status/1124043375437668352> | 09-Aug-19 |
| A202 | Nestle | Twitter | Information management Discursive strategy | Amplification Frame the debate | Unidos Colombia  @UnidosxNinosCol  May 27 Tomar más agua, comer y cocinar en familia, moverse más, escoger nutrición y manejar las porciones son algunos de los hábitos que puedes promover en tus hijos de manera divertida todos los días. Entérate de todo lo que tenemos para ti en http://bit.ly/2uWJXJe |  | <https://twitter.com/UnidosxNinosCol/status/1133095527191465985> | 09-Aug-19 |
| A203 | Nestle | Industry website | Information management Discursive strategy | Amplification Frame the debate | Alimentar el hábito (...) Claudia Cortés, gerente de nutrición, salud y bienestar de Nestlé, recuerda hábitos que aportan a esa búsqueda del equilibrio al comer, como incluir variedad de alimentos en las comidas diarias y tener un manejo adecuado de porciones; preocuparse porque al menos la mitad de los cereales que se consuman sean integrales e incluir lácteos, pues estos aportan nutrientes que ayudan a mantener el sistema óseo sano. Por supuesto, no pueden faltar los vegetales y frutas, beber entre uno y dos litros de agua al día, utilizar métodos adecuados de cocción y escuchar al cuerpo. La rutina diaria, además, tiene que acompañarse de pausas activas y algún tipo de actividad física. |  | <https://decidoloquecomo.com/buenos-habitos/alimentar-el-habito/> | 14-Aug-19 |
| A204 | Nestle | Industry website | Information management Discursive strategy | Amplification Frame the debate | Unidos por Niños Saludables está aquí para ayudar con un enfoque práctico a que los niños hagan más actividad física y desarrollen mejor sus habilidades de coordinación, aptitud física y fuerza a través del juego. Para comenzar:   • Destina tiempo para que los niños pasen más tiempo jugando al aire libre. • Promueve que practiquen deportes desde pequeños y hagan parte de algún equipo. • Crea actividades divertidas en casa para que tus hijos reduzcan el tiempo de pantalla y pasen menos tiempo sentados y acostados. • Estimula el juego con otros niños para ayudar a desarrollar habilidades sociales  Para formas sencillas y divertidas de adoptar este hábito saludable visita Ideas Saludables y la sección ¡Vamos a Cocinar! |  | <https://www.unidosporninossaludables.com.co/habitos_saludables/muevete_mas> | 19-Aug-19 |
| A205 | Nestle | Twitter | Information management | Amplification | Unidos Colombia @UnidosxNinosCol Estuvimos con los niños de tercero de la institución educativa José Eustacio Rivera aprendiendo sobre cómo manejar las porciones adecuadas. #UnidosPorNiñosSaludables /> 0:34 12:53 PM - 23 Jul 2019 |  | <https://twitter.com/UnidosxNinosCol/status/1153755131684540418> | 09-Aug-19 |
| A206 | Nutresa | Twitter | Coalition management | Community | Fundación Nutresa retwitteó Uaesp Cuenta verificada @Uaesp  12 jul. Junto con voluntarios de @FNutresa y Lime, la Alcaldía de @Bogota a través de la @Uaesp llevó a cabo una jornada de limpieza en el parque Veraguas realizando barrido, limpieza y pintura de postes; recolección de residuos y sensibilización con bolsa blanca y negra. |  | <https://twitter.com/Uaesp/status/1149800733837053954> | 07-Aug-19 |
| A207 | Nutresa | Twitter | Coalition management | Community | Grupo Nutresa S. A.  @Grupo_Nutresa Seguir Seguir a @Grupo_Nutresa #UnFuturoEntreTodos lo construimos desde la colaboración y solidaridad con nuestro planeta. Participa de la @CarreraVerdeCol y aportemos con la siembra de cerca de 7.500 árboles nativos #CarreraVerdeColombia #UnidosPorLosBosques 14:19 - 17 may. 2019 |  | <https://twitter.com/Grupo_Nutresa/status/1129496667739303936> | 07-Aug-19 |
| A208 | Nutresa | Twitter | Coalition management | Community | Fundación Nutresa retwitteó Asoc.BancoAlimentos @abacocolombia  12 feb. En la segunda etapa de la jornada; @Fundacion_Exito @Grupo_Exito nos brindo una capacitación en desnutrion crónica. @FNutresa nos ayuda a fortalecer nuestros procesos internos y externos, además de procesos éticos dentro de la Asociación. – en Conferencia Episcopal De Colombia |  | <https://twitter.com/abacocolombia/status/1095437878375735296> | 07-Aug-19 |
| A209 | Nutresa | Industry website | Coalition management | Health organisations and government bodies Internal | se desarrolla un piloto en Colombia a través de una alianza público-privada con el Ministerio de Educación, el Programa Mundial de Alimentos y Unicef que busca probar este modelo para beneficio de 20 entidades educativas, con miras a generar definiciones de política pública. | From 2016 | <http://informe2016.gruponutresa.com/vida-saludable/nutricion-y-vida-saludable-y-mercadeo-responsable/> | 20-Aug-19 |
| A210 | Nutresa | Industry website | Coalition management | Community | Fortalecer la gestión escolar e impulsar alianzas en educación. Se formaron maestros y directivos docentes de 494 colegios de Colombia, lo que permitió mejorar la administración escolar y la cultura institucional. El 73,9% de los colegios intervenidos en el 2016 con el programa Líderes Siglo XXI, obtuvo un desempeño satisfactorio en las pruebas de Estado y el 71% mantuvo o mejoró los resultados en el Índice Sintético de Calidad Educativa del Ministerio de Educación Nacional. Fomentar la competencia tecnológica de maestros y directivos. Se desarrollaron capacidades tecnológicas en 459 maestros y 59 directivos docentes de 20 colegios. El 89,8% de los beneficiarios logró un nivel óptimo de desempeño en competencias tecnológicas. | From 2016 | <http://informe2016.gruponutresa.com/ciudadania-corporativa/desarrollo-de-capacidades-y-educacion/> | 20-Aug-19 |
| A211 | Nutresa | Industry website | Coalition management | Community | Agregamos valor a la entrega de productos a los Bancos de Alimentos Somos parte activa los Bancos de Alimentos de Colombia, Costa Rica, México, Ecuador, Perú, República Dominicana, Estados Unidos y Chile. Nuestra labor es complementada por los Negocios y Voluntarios de Grupo Nutresa quienes participan del proceso de fortalecimiento institucional, a través de capacitaciones y desarrollo de habilidades.  La gestión con los Bancos de Alimentos en la región estratégica ha permitido reducir la pérdida y desperdicio de comida, impactar la disponibilidad local de alimentos y generar una economía solidaria, en donde las poblaciones de escasos recursos acceden a productos de alta calidad. Este trabajo es acompañado por un proceso de capacitación, monitoreo y evaluación en buenas prácticas de manufactura por parte de los voluntarios de las compañías especialistas en el tema. |  | <http://fundacionnutresa.com/nutricion/> | 20-Aug-19 |
| A212 | Nutresa | Industry website | Coalition management | Health and other organisations | Nutrición ENTIDAD PROYECTO 2018 Banco de Alimentos de Medellín Buen provecho Complementación Alimentaria para gestantes con bajo peso gestacional 2 6 Fundación Saciar Atención Integral para 370 Niños de Vallejuelos, Maruchenga y Andes. 5 7 Secretariado de pastoral social Comedor escolar para 236 niños 4 8 Fundación Bambi Atención integral de niños y niñas menores de un año hasta seis años de edad. 3 1  Nutriamor Medición del impacto nutricional de un nuevo desarrollo de la marca NUTRIAMOR® con altos aportes nutricionales. 3 1 Proyecto buen vecino Doria Estrategia de relacionamiento con vecinos planta"NUTREJUEGO DORIA: HACIA ESTILOS DE VIDA SALUDABLE” 4 7 |  | <http://fundacionnutresa.com/wp-content/uploads/Fundaci%C3%B3n-Nutresa-Informe-de-Gesti%C3%B3n-2018.pdf> | 20-Aug-19 |
| A213 | Nutresa | Industry website | Coalition management | Community | Construir una mejor sociedad ENTIDAD Proyecto 2018 Institución Educativa San Vicente ( premio Congreso) Premio Congreso Nacional de Educación 20 Fundación Victor Salvi Cartagena Festival Internacional de Música 2010 168 Secretos para Contar Secretos Para Contar, Educación Activa para el Campo Colombiano 59 Fundación Empresarios por la Educación Cuota Anual para Mejoramiento de la Gestión Escolar 127 Corporación Pueblo de los niños Protección Institucional Para Niños, Niñas y Adolescentes en Situación de Riesgo Sociofamiliar 24 Fundación La Cueva Carnaval Internacional de las Artes 47 Fundación Notas de Paz Orquesta Sinfónica Infantil y Juvenil Notas de Paz 29 Fundación Sura Becas Nicanor Restrepo Becas Nicanor Restrepo 21  Teatro Metropolitano Temporada Internacional de Música Clásica 63 Fundación Soleira Observatorio en derechos humanos de infancia y adolescencia 10  Alianza Colombo Francesa Fiesta de la Música 67 Teatro Metropolitano Sillas Adopción de 8 Sillas 50 Donación Proantioquia ( premio) Premio a la Educación 21 |  | <http://fundacionnutresa.com/wp-content/uploads/Fundaci%C3%B3n-Nutresa-Informe-de-Gesti%C3%B3n-2018.pdf> | 20-Aug-19 |
| A214 | Nutresa | Industry website | Coalition management Discursive strategy | Community Frame the debate | Este programa lúdico-pedagógico promocionó estilos de vida saludables en más de 15.000 niños de comunidades rurales y urbanas vulnerables, en las cuales los altos índices de inseguridad no facilitan las operaciones de la Organización. En materia rural se busca mejorar las condiciones de acceso a la educación de niños y niñas de comunidades campesinas con la entrega de 6.000 kits escolares, en donde la disponibilidad de los recursos para el aprendizaje es limitada. En lo urbano se promueve la integración social de los actores barriales con énfasis en la población infantil. | See pictures - branding - from 2016 | <http://informe2016.gruponutresa.com/ciudadania-corporativa/desarrollo-de-capacidades-y-educacion/> | 20-Aug-19 |
| A215 | Nutresa | Industry website | Coalition management Discursive strategy | Community Frame the debate | El reto en el 2017 será lograr la instalación de más de 5.000 m2 de huertas en Montes de María, Chocó y sierra nevada de Santa Marta. Adicionalmente, a corto plazo se seguirá implementando el plan de alimentación rural para que las comunidades beneficiadas no solo obtengan el alimento, sino que también conozcan sus propiedades y garanticen para sus miembros una alimentación balanceada acompañada de actividad física y prácticas claves de higiene.  Se afianzarán alianzas público-privadas con bancos de alimentos y otras entidades para desarrollar iniciativas de alto impacto que beneficien a las comunidades vulnerables. | From 2016 | <http://informe2016.gruponutresa.com/ciudadania-corporativa/seguridad-alimentaria-y-nutricion/> | 20-Aug-19 |
| A216 | Nutresa | Industry website | Coalition management Discursive strategy | Health organisations Frame the debate | Nutresa Quiere a los Niños promociona los estilos de vida saludable en pilares como alimentación balanceada, actividad física, hábitos de higiene y espacios para compartir en familia. En 2018 llegó a 92 sedes educativas en Colombia y benefició cerca de 6.000 estudiantes de zonas rurales de alta vulnerabilidad y difícil acceso. Adicionalmente, desarrolló en zonas urbanas 18 festivales artísticos comunitarios con la asistencia de 7.804 niños y niñas. | See pictures: branding | <https://s3.amazonaws.com/grupo-nutresa/wp-content/uploads/2019/04/02172007/informe-integrado-grupo-nutresa-2018.pdf> | 20-Aug-19 |
| A217 | Nutresa | Industry website | Coalition management Information management Discursive strategy | Health organisations Amplification Frame the debate | Alianzas por la nutrición En Fundación Nutresa y las empresa del Grupo Nutresa trabajamos en coherencia con nuestra filosofía corporativa de impulsar estrategias que promuevan estilos de vida y alimentación saludable, por ello junto al Ministerio Educación de Colombia, UNICEF, el Programa Mundial de Alimentos, el Instituto Nacional de Tecnología en Alimentos de la Universidad de Chile, el Instituto Nacional de Salud Pública Mexicano y el Instituto Mexicano Nacional de Pediatría, consolidamos la ruta de gestión escolar que busca la adopción de proyectos pedagógicos entorno a la alimentación y el cuidado de la vida, como una forma de prevenir o reducir los problemas de salud y las consecuencias de la desnutrición o mala alimentación de los niños y niñas en etapa escolar.  En Colombia hemos venido avanzando con la implementación del modelo de EVS (Estilos de Vida Saludable) en 20 establecimientos educativos de 10 secretarías de educación del país. | See more info in report - they use UNICEF logo etc | <http://fundacionnutresa.com/nutricion/> | 20-Aug-19 |
| A218 | Nutresa | Industry website | Discursive strategy | Frame the debate | Propósito Ofrecer productos y menús que brinden a los consumidores alternativas que cumplan con sus expectativas de nutrición y bienestar, y promover activamente estilos de vida saludable a través de campañas y programas de sensibilización y formación. El compromiso prioritario de Grupo Nutresa es incentivar el consumo responsable por medio de un etiquetado claro y una publicidad íntegra que permitan la toma de decisiones informadas por parte del consumidor. |  | <https://www.gruponutresa.com/sostenibilidad/fomentar-una-vida-saludable/> | 20-Aug-19 |
| A219 | Nutresa | Industry website | Discursive strategy | Frame the debate | Estrategia y Progreso Ajustar el perfil nutricional de los productos. Se alcanzó un total de 2.945 referencias ajustadas al perfil nutricional Nutresa, que corresponden al 63% del total de las ventas.   Implementar el rotulado nutricional de panel frontal en todos los productos. El 85,8% del portafolio quedó cubierto con etiquetado de panel frontal, lo que equivale a 3.432 referencias, atendiendo a la autorregulación en la mayoría de los casos y a los etiquetados obligatorios en donde estos están vigentes.  Promover estilos de vida saludable. Se continuó con el desarrollo de la campaña “Disfruta una Vida Saludable” y con el proyecto de formación en hábitos de vida saludable en la primera infancia en Colombia, así como con la estrategia de Espacios Saludables en México y Chile.  Disminuir los nutrientes de interés en salud pública. Se llevaron a cabo 143 reformulaciones en sodio, 78 en azúcar y 75 en grasas saturadas y 41 en grasas trans*.  Gestionar responsablemente la publicidad. Se aplicó la autorregulación publicitaria para menores de seis años y se estableció el compromiso para el 2017 de llevar la autorregulación hasta los 12 años. | See webpage for more details - but info seems to be from 2016  See also CSR report and these webpages:  https://www.gruponutresa.com/nutricion-y-vida-saludable/politica-de-nutricion-grupo-nutresa/ https://www.gruponutresa.com/nutricion-y-vida-saludable/perfil-nutricional-nutresa/ https://www.gruponutresa.com/nutricion-y-vida-saludable/guia-para-leer-nuestros-empaques/ | <http://informe2016.gruponutresa.com/vida-saludable/nutricion-y-vida-saludable-y-mercadeo-responsable/> | 20-Aug-19 |
| A220 | Nutresa | Industry website | Discursive strategy | Frame the debate | Grupo Nutresa ha entendido su responsabilidad y desde su compromiso con el consumidor viene adelantando una estrategia nutricional apoyada en la reformulación de productos, comunicación al consumidor, etiquetado, mercadeo responsable y promoción de estilos de vida saludable. (...) Esta realidad ha hecho que Grupo Nutresa avance en la apropiación de su política nutricional y continúe con sus programas de reformulación, ajuste al perfil nutricional, promoción de estilos de vida saludable, mercadeo responsable, comunicación al consumidor y rotulado de panel frontal. Para el 2020, Grupo Nutresa buscará mantener su liderazgo con alternativas de productos saludables y sostenibles, para esto continuará trabajando en alcanzar la meta de multiplicar por 2,5 veces la oferta de productos ajustados al perfil nutricional Nutresa frente a la línea base de 2012. | From 2016 | <http://informe2016.gruponutresa.com/vida-saludable/nutricion-y-vida-saludable-y-mercadeo-responsable/> | 20-Aug-19 |
| A221 | Nutresa | Industry website | Discursive strategy | Frame the debate | Propósito Promover el empoderamiento de las comunidades mediante el desarrollo de capacidades pedagógicas, de liderazgo y de gestión, en ambientes escolares y comunitarios, con el fin de fomentar la solidaridad, la transferencia de conocimiento y las redes de colaboración con voluntarios de las diferentes compañías de Grupo Nutresa, para contribuir a la disminución de la inequidad.  Propósito Diseñar y emprender iniciativas enfocadas a la erradicación del hambre que generen posibilidades de nutrición y desarrollo de capacidades en hábitos saludables como alimentación balanceada, prácticas de higiene efectiva y actividad física en poblaciones de escasos recursos. De igual forma, promover el acceso y la producción de alimentos a través de huertas comunitarias. |  | <https://www.gruponutresa.com/sostenibilidad/construir-una-mejor-sociedad/> | 20-Aug-19 |
| A222 | Nutresa | Industry website | Discursive strategy | Frame the debate | Bajo el mensaje “Disfrutar la vida te alimenta”, esta estrategia se soporta sobre cuatro pilares fundamentales: actividad física, alimentación balanceada, compartir en familia y disfrutar al aire libre.  El objetivo de esta campaña es promocionar la adopción de estilos de vida saludable a través de historias de la vida real y de la ejecución de actividades memorables de educación y concientización, con contenidos y mensajes relevantes, simples, positivos y propositivos.  Esta iniciativa se une a la estrategia de “realidad aumentada”, lanzada en el año 2013 y que busca fortalecer la toma informada de decisiones por parte de los consumidores. |  | <https://www.gruponutresa.com/nutricion-y-vida-saludable/estilos-de-vida-saludable/> | 20-Aug-19 |
| A223 | Nutresa | Industry website | Discursive strategy | Frame the debate | Fomentar una vida saludable *Base 2010. Por tonelada producida en Colombia. Productos con rotulado frontal (GDA) 2018: 86,4% 2017: 86,3% Producción fabricada en centros certificados 2018: 84,6% 2017: 79,2% Volumen de ventas de productos que cumplen el perfil nutricional Nutresa 2018: 68,2% 2017: 63,6% | Its own nutritional profile | <https://s3.amazonaws.com/grupo-nutresa/wp-content/uploads/2019/04/02172007/informe-integrado-grupo-nutresa-2018.pdf> | 20-Aug-19 |
| A224 | Nutresa | Industry website | Discursive strategy | Frame the debate | La adopción de los Objetivos de Desarrollo Sostenible de las Naciones Unidas es un tema prioritario en la agenda de trabajo global, particularmente de América Latina (...) En este sentido, Grupo Nutresa ha identificado en cada una de sus prioridades estratégicas, cómo puede contribuir más efectivamente al logro de los ODS y ha seleccionado los siguientes indicadores para mostrar su avance de cara al logro de las metas de la agenda 2030. | 300 pages long | <https://s3.amazonaws.com/grupo-nutresa/wp-content/uploads/2019/04/02172007/informe-integrado-grupo-nutresa-2018.pdf> | 20-Aug-19 |
| A225 | Nutresa | Industry website | Discursive strategy | Frame the debate | En la región estratégica, contribuimos con la disminución de la malnutrición y la promoción de estilos de vida saludable en las comunidades y a través del desarrollo de capacidades en los bancos de alimentos. Asimismo, fortalecimos prácticas clave de alimentación saludable, higiene y actividad física en comunidades escolares de Colombia, Chile y México. En cuanto a los retos de seguridad alimentaria, promovemos el autoabastecimiento de alimentos saludables en comunidades urbanas y rurales a través de nuestro programa Germinar. |  | <https://s3.amazonaws.com/grupo-nutresa/wp-content/uploads/2019/04/02172007/informe-integrado-grupo-nutresa-2018.pdf> | 20-Aug-19 |
| A226 | Nutresa | Industry website | Information management | Production | En Grupo Nutresa, la investigación en alimentación, nutrición y salud se enfoca en entender la relación entre la alimentación y los estados nutricionales, para que las alternativas planteadas aporten a la solución. Hoy, el centro de investigación Vidarium se encuentra abordando, dentro de los asuntos centrales relacionados a ECNT, el tema de obesidad a través de sus líneas de investigación en microbiota y antioxidantes, y el de salud cardiovascular a través de la línea de antioxidantes. | From 2016 | <http://informe2016.gruponutresa.com/vida-saludable/nutricion-y-vida-saludable-y-mercadeo-responsable/> | 20-Aug-19 |
| A227 | Nutresa | Industry website | Information management | Production Amplification | En alianza con la Universidad de Antioquia, Fundación Nutresa desarrolló su primera cartilla didáctica de promoción de estilos de vida saludable para niños y niñas de zonas rurales de alto riesgo social. Esta herramienta es entregada en el marco del Programa Nutresa Quiere a los Niños en Colombia, y promueve la apropiación de estilos de vida saludable como alimentación balanceada, actividad física, prácticas claves de higiene y compartir en familia | See webpage for more details - but info seem to be from 2016 | <http://informe2016.gruponutresa.com/ciudadania-corporativa/seguridad-alimentaria-y-nutricion/> | 20-Aug-19 |
| A228 | Nutresa | Industry website | Information management | Production | INVESTIGACIÓN EN NUTRICIÓN, SALUD Y BIENESTAR En Grupo Nutresa, la investigación como componente del modelo de innovación se lleva a cabo desde los centros de investigación de los diferentes negocios, y de la Corporación Vidarium -Centro de investigación en nutrición, salud y bienestar. Este último tiene como propósito la generación de conocimiento científico en alimentación, salud y nutrición, y la gestión en la transferencia de conocimiento para aportar al desarrollo de los negocios de Grupo Nutresa y al mejoramiento de la calidad de vida en los diferentes países que componen su región estratégica.  Vidarium prioriza como temas de investigación la obesidad, la salud cardiovascular y la salud gastrointestinal, a través de tres líneas de investigación: modulación de procesos oxidativos; moduladores alimentarios de la microbiota; y componentes alimentarios y otras sustancias. www.vidarium.org | There is a specific website for the Vidarium | <https://www.gruponutresa.com/nutricion-y-vida-saludable/investigacion-en-nutricion-salud-y-bienestar/> | 20-Aug-19 |
| A229 | Nutresa | Industry website | Information management Discursive strategy | Amplification Frame the debate | 107 NIÑOS DEL CENTRO RURAL LOS MICOS en Colombia se divierten mientras aprenden de alimentación y hábitos de vida saludable a través del programa Nutresa Quiere a los Niños |  | <https://s3.amazonaws.com/grupo-nutresa/wp-content/uploads/2019/04/02172007/informe-integrado-grupo-nutresa-2018.pdf> | 20-Aug-19 |
| A230 | PepsiCo | Industry website | Coalition management | Community | Proyectos para dar acceso al agua potable Proyecto que se desarrolla en Fuente de Vida Malambo en alianza con Postobón y la fundación Empresas Públicas de Medellín. La iniciativa busca llevar agua potable a los habitantes de Malambo. Se beneficiarán 4.080 viviendas en este municipio. |  | <http://www.pepsico.com.co/que-creemos/fundacion-pepsico> | 14-Aug-19 |
| A231 | PepsiCo | Twitter | Discursive strategy | Frame the debate | Bebidas de tu lado @bebidasdetulado  13 juil. “A2 Aliméntate y actívate” es un proyecto de la Fundación PEPSICO y COMPENSAR que prueba que las bebidas estamos #DeTuLado por una #VidaBalanceada. |  | <https://twitter.com/bebidasdetulado/status/1149853449892388865> | 12-Aug-19 |
| A232 | PepsiCo | Industry website | Discursive strategy | Economy | Somos una compañía que genera empleo en Colombia, junto con nuestros socios empleamos aproximadamente 16,066 personas. |  | <http://www.pepsico.com.co/quienes-somos/pepsico-col> | 14-Aug-19 |
| A233 | PepsiCo | Conference | Information management | Amplification | **ACODIN Congreso 2019 - Dia 2 SIMPOSIO (17) ENTRENAMIENTO, FITNESS Y NUTRICIÓN**  MODERADOR: MARIA VICTORIA BENJUMEA ND U. DE ANTIOQUIA -PHD Escuela Nacional De Salud Pública Cuba  Importancia de la hidratación en la práctica deportiva Viviana Viviant ND. ARGENTINA **Ejercicio: el aliado clave en un plan de alimentación Carolina Peña Ochoa. ND. Gatorade.** COLOMBIA  Suplementos: indicaciones y contraindicaciones Maximiliano Kammerer ND MSc. U CES. COLOMBIA  Periodización nutricional en deportistas Ligia Guerrero ND PhD. UNal. COLOMBIA |  | https://drive.google.com/file/d/15wsLa88rSWC4t3va9O-jAgHxY3xCJBRK/view?usp=sharing | 06-Aug-19 |
| A234 | PepsiCo | Industry website | Information management | Amplification | Alimentate y activate En los municipios de Funza y Madrid Cundinamarca, el programa Aliméntate y Actívate, liderado por PepsiCo y La Caja de Compensación Familiar Compensar, trabaja en la promoción de hábitos y estilos de vida saludable. Durante los tres años de ejecución del programa se ha logrado la adquisición y apropiación de conocimientos en hábitos y estilos de vida saludable por parte de los beneficiarios en un 91%, igualmente se han realizado 386 talleres con estudiantes y 15 talleres con padres de familia. Desde 2015 2.427 alumnos han sido evaluados, más de 1.700 familias y 240 miembros del personal escolar se han beneficiado de este programa. Gracias a este proyecto, el 53% de los estudiantes priorizados mostraron un cambio positivo, ya que todos ellos mejoraron sus mediciones iniciales.  Nutrición para el futuro El programa "Nutrición para el futuro" está alineado con la agenda de PepsiCo Desempeño con Propósito y tiene como objetivo brindar acceso a por lo menos 3 mil millones de porciones de alimentos y bebidas nutritivas para las comunidades y consumidores marginados de todo el mundo. La compañía está comprometida a lograr esto a través de una variedad de programas apoyados por la Fundación PepsiCo, con un enfoque creciente en productos locales para cada región. Recientemente anunciamos la donación de $500,000 dólares de la Fundación PepsiCo a la Red Mundial de Bancos de Alimentos para su programa: "Expandir el acceso a alimentos nutritivos". Específicamente, en América Latina, este programa proporcionará 6,75 millones de porciones nutritivas durante 18 meses. Hasta la fecha, PepsiCo Latinoamérica y la Fundación PepsiCo han beneficiado a 20,000 personas en la región. |  | <http://www.pepsico.com.co/que-creemos/fundacion-pepsico> | 14-Aug-19 |
| A235 | Postobon | Media | Coalition management | Community | Programas de Postobón promueven el reciclaje y la educación en Bahía Solano miércoles, 13 de marzo de 2019  Desde 2015 con el programa ‘Mi pupitre postobón' se han beneficiado 49.200 estudiantes de 69 colegios.  Con el fin de contribuir con el manejo de residuos sólidos y el mejoramiento de las condiciones educativas, Postobón llegó a Bahía Solano en Chocó con los programas ‘Recicla por el planeta’ y ‘Mi pupitre Postobón’.  ‘Mi pupitre Postobón’, con inversión de $80 millones, entregó 486 piezas de mobiliario escolar hecho con material reciclado de Tetra Pak que beneficiarán a 1.500 estudiantes de la Institución Educativa Luis López de Mesa. El mobiliario se realizó con 1,7 millones de cajitas de Hit y Tutti Frutti, “evitando que lleguen a los rellenos sanitarios”, destacó Postobón |  | <https://www.larepublica.co/empresas/programas-de-postobon-promueven-el-reciclaje-y-la-educacion-en-bahia-solano-2838992> | 06-Aug-19 |
| A236 | Postobon | Twitter | Coalition management | Community | Postobón empresa @postobonoficial  Jun 22 Nuestra meta es que jóvenes y niños tengan la facilidad de seguir estudiando para cumplir sus más grandes metas. Esto es posible gracias a #MiBiciPostobón |  | <https://twitter.com/postobonoficial/status/1142687481013260288> | 07-Aug-19 |
| A237 | Postobon | Twitter | Coalition management | Community | Postobón empresa @postobonoficial  Jun 5 100 bicicletas del programa #MiBiciPostobón, se entregaron a jóvenes de los municipios de Samaná, La Victoria, Manzanares, Marquetalia y el corregimiento de San Diego, esto gracias a la alianza entre la Caja de Compensación Familiar de Caldas y Postobón |  | <https://twitter.com/postobonoficial/status/1136376684884705281> | 07-Aug-19 |
| A238 | Postobon | Twitter | Coalition management | Community | Postobón empresa @postobonoficial Follow Follow @postobonoficial More Estamos presentes en la Institución Educativa Tricentenario impactando cerca de 1.200 estudiantes con el programa #MiPupitrePostobón ¡Nuestro compromiso es seguir ayudando a cumplir sueños! 🙌 2:05 9:42 AM - 9 May 2019 |  | <https://twitter.com/postobonoficial/status/1126527951053496321> | 07-Aug-19 |
| A239 | Postobon | Twitter | Coalition management | Community | Postobón empresa ‏@postobonoficial  Mar 27 More Postobón empresa Retweeted Telemedellín #MiBiciPostobón presente en la Institución Educativa Santa Elena 🚴‍♂️👏🎓 Ahora la historia de los niños de esta institución comenzará a cambiar 😄🤓 |  | <https://twitter.com/postobonoficial/status/1110901368037011456> | 07-Aug-19 |
| A240 | Postobon | Twitter | Coalition management | Community Government (first lady from Medellin municipality) | Postobón empresa Retweeted  Comfama  @Comfama  Mar 26 More 🚴‍♀️🚵‍♀️📸📷 ¡Dotar de sentidos! Junto a @postobonoficial y la Primera Dama de Medellín, Margarita María Gómez, entregamos 242 bicicletas en #SantaElena. 👉 Conoce más detalles de #MiBiciPostobón en este enlace https://bit.ly/2HUCWk0 https://twitter.com/Comfama/status/1110586237419692033/photo/1pic.twitter.com/ZmdsbR6ARx |  | <https://twitter.com/Comfama/status/1110612266624397312> | 07-Aug-19 |
| A241 | Postobon | Twitter | Coalition management | Community | Comfama ‏   @Comfama Follow Follow @Comfama "Esta institución educativa ha venido progresando de forma significativa en todas las pruebas. Con @postobonoficial queremos aportar juntos a una mejor transformación", resalta Silvia Ochoa, responsable de Empresas @Comfama.  8:51 AM - 26 Mar 2019 | Much much more on the Tweeter feed - see pictures: branding | <https://twitter.com/Comfama/status/1110569946021617664> | 07-Aug-19 |
| A242 | Postobon | Twitter | Coalition management | Community | Postobón empresa @postobonoficial  Mar 6 Nuestro compromiso es mejorar las condiciones educativas de las escuelas rurales del país y acercar a jóvenes y niños para cumplir sus sueños. #NuestroCompromiso 🤚🏻 https://bit.ly/2SvFYg6 |  | <https://twitter.com/postobonoficial/status/1103324364097773569> | 07-Aug-19 |
| A243 | Postobon | Twitter | Coalition management | Community | Postobón empresa @postobonoficial  Feb 24 Contar con un buen entorno para el aprendizaje es un factor importante para lograr que nuestros niños y niñas alcancen sus sueños. #MiPupitrePostobón invita a pensar en grande.✍👏🤓👧 |  | <https://twitter.com/postobonoficial/status/1099780254661398528> | 07-Aug-19 |
| A244 | Postobon | Industry website | Coalition management | Community | MiPupitre Postobón Uno más Todos también es pensar en la educación de los colombianos. Por eso, con el apoyo de Agua Oasis, Postobón S.A contamos con el programa MiPupitre, un proyecto de alto impacto que busca aportar al mejoramiento de las condiciones de mobiliario en las instituciones educativas del país. El mobiliario de MiPupitre es hecho con material de Tetrapak reciclado, lo cual representa un aporte al medio ambiente al reutilizar este insumo. Además, la comunidad podrá participar con la recolección de cajas en diferentes puntos de las ciudades, iniciando con Medellín y Bogotá, donde se instalarán en alianza con Tetrapak, 2.896 puntos de recolección en colegios, instituciones educativas, universidades y centros comerciales, entre otros, en los que se harán actividades de sensibilización y cocreación para que los estudiantes, padres de familia y comunidad en general, gestionen la posibilidad de adquirir el mobiliario de MiPupitre. El programa entregará además, dotación en 13 instituciones educativas de Atlántico, Bolívar, Cesar, Boyacá y Antioquia, beneficiando a 8.200 niños, niñas y jóvenes. A esto se suma la intervención a la infraestructura física de las escuelas, haciendo que Mi Pupitre Postobón sea un programa integral con alto impacto en la educación y el medio ambiente. La característica principal del mobiliario, que cumple con todas las normas que el Ministerio de Educación define en temas de dotación escolar y que puede ser hasta un 30% más económico que el mobiliario tradicional para escuelas y colegios, es su alta resistencia que lo protege de la humedad y los cambios de temperatura. | See webpage for more info See pictures | <https://www.postobon.com/sostenibilidad/mipupitre-postobon> | 19-Aug-19 |
| A245 | Postobon | Industry website | Coalition management | Community | MiBici Postobón El programa MiBici hace parte de la iniciativa Uno más Todos de Postobón. Este programa entrega a niños y jóvenes bicicletas diseñadas especialmente para la ruralidad colombiana con el fin de que este medio de transporte se convierta en una herramienta integral para transformar sus vidas. Anualmente, Postobón entregará alrededor de 2.000 bicicletas por año. | See also programme in specific cities: https://www.postobon.com/sala-prensa/noticias/mibici-y-mipupitre-postobon-llegan-cordoba https://www.postobon.com/sala-prensa/noticias/mibici-y-mipupitre-en-cordoba | <https://www.postobon.com/sostenibilidad/mibici-postobon> | 19-Aug-19 |
| A246 | Postobon | Industry website | Coalition management | Community | iPupitre entrega 745 piezas de mobiliario escolar en la inauguración de la I.E Tricentenario de Medellín 09 de Mayo de 2019 La institución fue ampliada por la Alcaldía de Medellín para atender un mayor número de niños de la zona norte de la ciudad, con una inversión que ascendió a $10.365 millones . Esta institución se fundó en 1978 como sede de los trabajadores que construían la unidad Tricentenario, donde habitaron los deportistas participantes de los XIII Juegos Centroamericanos y del Caribe. Luego de los juegos, se convirtió en institución educativa. En esta ocasión, el programa MiPupitre Postobón, impactó cerca de 1.200 estudiantes, con la entrega de 745 piezas de mobiliario escolar entre sillas y pupitres, para lo cual se usaron 17 toneladas de Tetra Pak para hacer el mobiliario, que equivalen a 1,4 millones de cajitas, para esto la compañía realizó una inversión de $106 millones, por intermedio de la Fundación Postobón. Nuestro programa nació en 2015, cuando nos propusimos avanzar en el mejoramiento de las condiciones de estudio de los niños, niñas y jóvenes colombianos y contribuir al mismo tiempo al cuidado del medio ambiente. (...) Desde 2015 a la fecha, MiPupitre ha entregado 25.815 piezas de mobiliario entregadas, beneficiando a 47.705 estudiantes en 12 departamentos del país, logrando reciclar 1.137 toneladas de Tetra Pak, que equivalen a 122 millones de cajitas de 200ml, las cuales se convirtieron en oportunidades de educación. | See also programme in other cities: https://www.postobon.com/sala-prensa/noticias/postobon-llega-bahia-solano-choco-los-programas-recicla-el-planeta-y-mipupitre | <https://www.postobon.com/sala-prensa/noticias/mipupitre-entrega-745-piezas-mobiliario-escolar-en-la-inauguracion-la-ie> | 19-Aug-19 |
| A247 | Postobon | Industry website | Coalition management | Community | Desde 2014, cuando se creó el programa, se han reciclado más de 122 millones de cajitas de Tetra Pak. Con este programa reciclamos las cajitas de Tetra Pak para fabricar mobiliario escolar para nuestros niños y jóvenes en condiciones pobreza extrema. Si algo puede marcar la educación de un niño son las condiciones del entorno para el aprendizaje. Conscientes de eso, diseñamos MiPupitre Postobón, que busca mejorar las condiciones de acceso a la educación de los niños y jóvenes del país que habitan en condiciones de pobreza extrema. Con este programa entregamos mobiliario escolar fabricado con cajitas de Tetra Pak de Hit, Tutti Frutti y Mr. Tea. Estas piezas son resistentes a la humedad y a los cambios de temperatura. Además, son 100% reciclables, garantizando así ciclos cerrados de producción. MiPupitre Postobón también incentiva el aumento en los volúmenes de recolección de Tetra Pak, para desarrollar así un impacto ambiental positivo. 25.815 Piezas de mobiliario escolar entregado. (....) 47.705 Estudiantes beneficiados. | See also CSR report: https://www.postobon.com/sites/default/files/resumen_informe_de_sostenibilidad_2018_0.pdf | <https://fundacionpostobon.com/programas/mipupitre-postobon/> | 19-Aug-19 |
| A248 | Postobon | Twitter | Coalition management Discursive strategy | Community Frame the debate | Postobón empresa Retweeted Agenda del Mar @LaAgendadelMar  May 18 Hoy compartimos con los niños de la Sierra Nevada de Santa Marta en un taller de educación ambiental. Gracias a @wirakoku @maajiswimwear @SoyTosh @postobonoficial @LOccitane_CO #EarthWarriors #SomosGuardianesDeLosOcéanos |  | <https://twitter.com/LaAgendadelMar/status/1129908134343041024> | 07-Aug-19 |
| A249 | Postobon | Twitter | Coalition management Discursive strategy | Community Government (first lady from Medellin municipality)  Frame the debate | Postobón empresa @postobonoficial  Jan 25 More La Fundación Postobón, con el apoyo de la Secretaría de Educación de Medellín, lanzó el programa ¡Boom! Activa tu vida, el cual iniciará con actividades en 60 instituciones educativas oficiales y privadas de la ciudad 👉 ¡Entérate de más en la... https://www.postobon.com/sala-prensa/noticias/boom-activa-tu-vida-la-nueva-iniciativa-la-fundacion-postobon-para-promover … |  | <https://twitter.com/postobonoficial/status/1088940128477827072> | 07-Aug-19 |
| A250 | Postobon | Industry website | Coalition management Discursive strategy | Community Frame the debate | ¡Boom! Activa tu vida, la nueva iniciativa de la Fundación Postobón para promover hábitos de vida responsables 25 de Enero de 2019 Con el fin de promover hábitos de vida responsables en jóvenes entre los 12 y 17 años, la Fundación Postobón, con el apoyo de la Secretaría de Educación de Medellín, lanzamos este viernes 25 de enero de 2019 en Medellín el programa ¡Boom! Activa tu vida, el cual iniciará con actividades en 60 instituciones educativas oficiales y privadas de la ciudad, las cuales fueron seleccionadas de un universo de 120 instituciones, en un trabajo que se hizo con el acompañamiento de la Secretaría de Educación de Medellín, abarcando así a más de 9 mil jóvenes beneficiados. El objetivo del programa es promover en los estudiantes el gusto por la actividad física y dejar a un lado las barreras que impiden su realización. La metodología que desarrollará el programa ofrece un acompañamiento integral a las instituciones educativas para que tengan mayores elementos y argumentos que motiven y sensibilicen a los jóvenes, docentes y familias sobre los beneficios de la actividad física, además de brindar herramientas técnicas y pedagógicas para implementar buenas prácticas y, realizar, con el apoyo de los docentes de educación física y facilitadores, actividades rítmicas, ejercicios con TRX, entrenamientos funcionales y circuitos de ejercicio, entre otros, luego de la jornada académica. (...) La presentación del programa se realizó en el auditorio del Edificio Coltejer y contó con la participación de los rectores y profesores de educación física de las intuiciones seleccionadas, así como la presencia del secretario de Educación de Medellín, Luis Guillermo Patiño. Durante el evento, los rectores y docentes recibieron información sobre la relevancia de actividad física y los beneficios que tiene su promoción en la vida de los jóvenes. (...) Con el fin de promover hábitos de vida responsables, la Fundación Postobón, con el apoyo de la Secretaría de Educación de Medellín, lanzó este viernes 25 de enero de 2019 en Medellín el programa ¡Boom! Activa tu vida. "Con este programa, la Fundación Postobón va a aportar a las instituciones educativas seleccionadas, herramientas que les permitan a nuestros jóvenes entender que a través de la actividad física pueden avanzar en sus proyectos de vida", dijo al respecto Luis Guillermo Patiño, secretario de Educación de Medellín durante la presentación del programa. | See this webpage as well: https://tomatelavida.com.co/boom-activa-tu-vida/ | <https://www.postobon.com/sala-prensa/noticias/boom-activa-tu-vida-la-nueva-iniciativa-la-fundacion-postobon-para-promover> | 19-Aug-19 |
| A251 | Postobon | Industry website | Coalition management Discursive strategy | Community Frame the debate | Distribuimos bicicletas diseñadas especialmente para la ruralidad, con el fin de que se conviertan en una herramienta integral para transformar vidas. MiBici Postobón distribuye a niños y jóvenes de Colombia, bicicletas diseñadas especialmente para la ruralidad colombiana, para que pedaleen hacia sus sueños y mejoren su acceso a la educación. Este programa plantea dos objetivos: 1. Disminuir la inasistencia y deserción escolar rural. 2. Mejorar el desempeño escolar de los niños y adolescentes ubicados en la ruralidad dispersa. Los estudiantes que reciben este medio de transporte, reducen sus tiempos de desplazamiento entre la escuela y el hogar entre un 40% y 60%. Las bicicletas de MiBici Postobón son de marca Buffalo y fueron diseñadas por la marca SRAM, líder mundial en accesorios de bicicletas, pensando en las zonas rurales y la base de la pirámide. El diseño de las bicicletas de marca Buffalo, es el resultado del trabajo que desarrolla la fundación norteamericana World Bycicle Relief, aliada estratégica de Postobón en el programa. 8.405 Bicicletas entregadas desde 2014. 3,452 Bicicletas entregadas en 2018. 1.800 de ellas, gracias a la alianza con Comfama. 12 Departamentos impactados. 179 Instituciones educativas públicas beneficiadas. | See also CSR report: https://www.postobon.com/sites/default/files/resumen_informe_de_sostenibilidad_2018_0.pdf | <https://fundacionpostobon.com/programas/mibici-postobon/> | 19-Aug-19 |
| A252 | Postobon | Media | Coalition management Discursive strategy | Community Frame the debate | TRANSPORTE El programa MiBici Postobón ha entregado más de 8.000 bicicletas en cerca de cinco años martes, 30 de julio de 2019 Diferentes iniciativas en el país promueven el uso de la bicicleta en los niños para desplazarse hasta sus colegios. Hoy por hoy, son muchos los proyectos que incentivan el uso de la bicicleta para que los niños se desplacen a sus colegios. En Medellín, por ejemplo, la campaña que ha tenido el impacto más notorio es MiBici, liderada por Postobón. La operación en Antioquia es solo una parte de la campaña MiBici, que también se enfoca en zonas rurales de La Guajira, Cundinamarca, Putumayo, Cesar, Valle, Cauca, Bolívar y Caldas, según comentó la directora de sostenibilidad de Postobón, Martha Falla. En los casi cinco años que suma esta campaña, se han entregado 8.700 bicicletas, cuyo valor asciende a $800.000 cada una. En total, el costo de las mismas asciende a $6.960 millones. |  | <https://www.larepublica.co/especiales/especial-rse-julio-2019/el-programa-mibici-postobon-ha-entregado-mas-de-8000-bicicletas-en-cerca-de-cinco-anos-2890569> | 06-Aug-19 |
| A253 | Postobon | Media | Coalition management Discursive strategy | Community Frame the debate | Postobón invirtió más de $18.500 millones en proyectos sociales y ambientales miércoles, 22 de mayo de 2019 La inversión destinada por la compañía en 2018 fue 39% más que en 2017  Ayer, Postobón presentó su informe de sostenibilidad de 2018, el cual mostró el impacto de sus programas e iniciativas sociales y ambientales en el territorio nacional. Según el documento, Postobón benefició directamente a más de 500.000 colombianos en 16 departamentos del país con una inversión de más de $18.500 millones en programas sociales y ambientales. Las zonas del territorio nacional en las que estuvieron presentes los programas de Postobón fueron Antioquia, Bolívar, Caldas, Cauca, Chocó, Córdoba, Cundinamarca, Guajira, Nariño, Norte de Santander, Risaralda, Tolima, Santander, Sucre, Putumayo y Valle del Cauca. Vale la pena resaltar que a las personas beneficiadas se les debe sumar las más de seis millones de personas sensibilizadas con actividades recreativas, enfocadas en la promoción de estilos de vida activos. “Nos enorgullece presentar una vez más nuestro Informe de Sostenibilidad que da cuenta del gran compromiso que tenemos con Colombia. Durante 2018, invertimos 39% más que en 2017, cifra que tiene mucha relevancia para el progreso de las comunidades donde tenemos influencia”, señaló Miguel Fernando Escobar, presidente de Postobón. Los programas que tiene la empresa para trabajar por la equidad en el país se dividen en varios frentes. Por ejemplo, en materia educativa están planes como ‘MiBici’ y ‘MiPupitre Postobón’ que beneficiaron a más de 56.100 estudiantes en 12 departamentos. También está el programa ‘Hit Social Postobón’ que está enfocado en el apoyo a agricultores de fruta. Con este se beneficiaron 1.358 familias de Bolívar, Santander, Risaralda, Valle, Cauca y Tolima, según el informe de la firma. | See also:  https://www.larepublica.co/responsabilidad-social/el-programa-mibici-postobon-ya-ha-entregado-1272-bicicletas-en-antioquia-2844822 https://www.rcnradio.com/colombia/antioquia/la-educacion-llega-en-dos-ruedas-santa-rosa-de-osos-con-mi-bici-de-postobon https://www.larepublica.co/responsabilidad-social/mibici-postobon-entrego-100-bicis-en-santa-rosa-de-osos-en-antioquia-2839386 | <https://www.larepublica.co/empresas/postobon-invirtio-mas-de-18500-millones-en-proyectos-sociales-y-ambientales-2864934> | 06-Aug-19 |
| A254 | Postobon | Industry website | Discursive strategy | Frame the debate | Como sabemos, los hábitos son acciones que se convierten en nuestra cotidianidad, debido a las repeticiones. Por esto, es mucho mejor crear hábitos responsables en la infancia, ya que se convierte en un estilo de vida para siempre, ya que los niños interiorizan más fácil las rutinas y le cogen amor desde que las inician.Entre los hábitos más necesarios están el comer bien, hacer deporte y beber agua, son rutinas que debemos enseñar a los niños desde que están pequeños para llevar una vida sana, esto apoyará la prevención de enfermedades como el sobrepeso. A continuación de mostraremos 5 hábitos responsables que puedes enseñar a los más pequeños, para que interioricen y crezcan de la mejor manera: 1. Alimentación variada y equilibrada: esto garantiza que los niños obtengan los nutrientes necesarios para su desarrollo y crecimiento. Por esto es importante animarlos a probar cosas nuevas, ya que el gusto de los pequeños es cambiante y poco a poco aceptaran alimentos que antes no deseaban comer. Asimismo es importante brindar un equilibrio en las porciones de alimentos, proporcionando frutas, verduras, proteínas, cereales y una constante hidratación, también debemos tener en cuenta la importancia de establecer horarios, planear las comidas, compartir en familia y hacer cenas originales y creativas. 2. Beber agua: esta es necesaria para eliminar toxinas e hidratar los órganos, por esto debemos tenerla como la primera opción de bebida en nuestras cenas y llevarla siempre con nosotros para una ingesta constante. 3. Buenas costumbres al alimentarse: debemos enseñarles a los niños a comer lo necesario según sus necesidades, para esto no debemos obligarlos a comer más de lo que pueden y debemos ofrecerles un espacio idóneo para alimentarse. 4. Disminuir la televisión: el tiempo es muy valioso y en vez de ver televisión, podrían estar realizando alguna actividad física, recuerda que el tiempo recomendado para que los niños vean televisión es de máximo 2 horas al día. 5. Actividad física: el juego es un aliado para el funcionamiento del organismo, ya que brinda beneficios similares a los del deporte, por esto procuremos que los niños tengan una hora al día de juegos y deportes para mejorar su estado de animo e incrementar su actividad física. Te recomendamos que estos planes deportivos sean en familia, no sean obligatorios y lo más importante es que sean divertidos para todos. | Many other examples on the website | <https://tomatelavida.com.co/noticias/habitos-saludables-para-ninos/> | 20-Aug-19 |
| A255 | Postobon | Media | Discursive strategy | Frame the debate | Al explicar las motivaciones para ser patrocinador, Postobón afirmó que es coherente con su objetivo de promover el deporte y apoyar las selecciones del país en diferentes modalidades. Además, Colombiana se une a Manzana Postobón, marca que patrocina a las selecciones nacionales de ciclismo, patinaje y béisbol.  “Estamos muy orgullosos de que una marca como Colombiana, que representa tanto para el país, llegue de nuevo al fútbol, y qué mejor que apoyando a las selecciones Colombia de este deporte como socios oficiales...”, manifestó Miguel Fernando Escobar, presidente de la compañía.  Postobón se ubica entre los aliados de la Selección, tales como Adidas, Movistar (Telefónica), Homecenter, Bancolombia, Allianz, Avianca, Caracol Televisión y Servientrega, a los que se les vencieron sus contratos en diciembre del año pasado, y se está a la espera de una renovación. Por ahora, el único patrocinador del equipo es Bavaria, en cabeza de su marca Cerveza Águila.  En 2014, Postobón anunció apoyo a la actividad física, en la cual incluía múltiples disciplinas, desde lo profesional hasta lo social. |  | https://www.eltiempo.com/economia/empresas/acuerdo-de-postobon-para-patrocinar-la-seleccion-colombia-324298 | 06-Aug-19 |
| A256 | Postobon | Media | Discursive strategy | Frame the debate | Desde el año 2016, la empresa ha firmado un compromiso de autorregulación que incluye cinco puntos, el primero es la no comercialización de bebidas azucaradas en colegios y en escuelas primarias, en segundo lugar han incorporado el etiquetado GDA en todas sus bebidas y han habilitado un portal para explicar cómo hacer correctamente la lectura de la información nutricional.  Esta iniciativa de autorregulación también ha impactado su portafolio de mas de 35 marcas, y por tal motivo, han decidido sacar al mercado nuevas alternativas de bebidas más saludables, que pretenden facilitar las decisiones consientes de consumo.   Hoy, el 52% de las ventas son generadas por productos diferentes a las gaseosas, y el 72% del portafolio en general es “reducido, bajo o libre de calorías”. Otra de las medidas adoptadas por la compañía en medio de su política de autorregulación, es la directriz de no hacer publicidad dirigida a menores de 12 años y finalmente, buscan promocionar estilos de vida saludables y responsables. |  | <https://www.dinero.com/empresas/articulo/postobon-se-la-juega-por-la-autorregulacion-y-la-sostenibilidad/272459> | 06-Aug-19 |
| A257 | Postobon | Media | Discursive strategy | Frame the debate | Actualmente, contamos con 35 marcas y cerca de 500 presentaciones. De acuerdo con Nielsen, somos líderes en gaseosas en el segmento de sabores y en la categoría de aguas, bebidas con fruta, té listo para beber e hidratantes. (…) Como industria y de la mano de la ANDI, en 2016, varias empresas de sector llegamos a un acuerdo con el gobierno que incluye cinco compromisos enfocados en promover estilos de vida activos y saludables. A la fecha, desde Postobón avanzamos en el cumplimiento y en la implementación de las acciones derivadas de los compromisos. Lo hacemos con responsabilidad y claridad frente a su implementación. Además, quiero destacar que trabajamos con el Ministro de Salud, para alinear a las políticas públicas del gobierno estos compromisos y las iniciativas que tenemos como compañía en la promoción de estilos de vida activos y responsables. |  | <https://www.larepublica.co/empresas/nos-hemos-adaptado-a-las-tendencias-de-los-consumidores-dijo-el-presidente-de-postobon-2826787> | 06-Aug-19 |
| A258 | Postobon | Twitter | Discursive strategy | Frame the debate | Postobón empresa @postobonoficial  May 14 More Postobón empresa Retweeted Bebidas de tu lado El 100% de nuestras bebidas tiene GDA. Estamos comprometidos con #InformaciónClara. 👏🤓 @bebidasdetulado |  | <https://twitter.com/postobonoficial/status/1128372364574646273> | 07-Aug-19 |
| A259 | Postobon | Twitter | Discursive strategy | Frame the debate | Postobón empresa ‏@postobonoficial  Apr 11 More Hacer un poco de actividad física al día trae muchos beneficios para tu bienestar. ¡No pierdas más el tiempo! Mueve tu cuerpo y #ActivaTuVida |  | <https://twitter.com/postobonoficial/status/1116309926698258432> | 07-Aug-19 |
| A260 | Postobon | Twitter | Discursive strategy | Frame the debate | Postobón empresa Retweeted CECODES @CECODES  Apr 9 Miguel Fernando Escobar, presidente de @postobonoficial habla de la responsabilidad que tiene la compañía con la actividad física.  ¿Quieres conocer más? 👉 http://ow.ly/lh7m50pE9Ek |  | <https://twitter.com/CECODES/status/1115680820365283329> | 07-Aug-19 |
| A261 | Postobon | Twitter | Discursive strategy | Frame the debate | Postobón empresa @postobonoficial  Apr 7 More ¡Cuídate más! Mueve tu cuerpo y haz pausas activas. Cuando lo haces tu vida se llena de bienestar. 🤓✋👏 |  | <https://twitter.com/postobonoficial/status/1114935873135697920> | 07-Aug-19 |
| A262 | Postobon | Twitter | Discursive strategy | Frame the debate | Postobón empresa ‏@postobonoficial  Apr 4 More Hacer un poco de actividad física al día trae muchos beneficios. ¿qué estás esperando? #ActivaTuVida |  | <https://twitter.com/postobonoficial/status/1113773211097935872> | 07-Aug-19 |
| A263 | Postobon | Twitter | Discursive strategy | Frame the debate | Postobón empresa  @postobonoficial  Mar 1 Buscamos promover estilos de vida activos y acompañar a las personas, para que construyan hábitos responsables que propicien su bienestar. |  | <https://twitter.com/postobonoficial/status/1101452023017492480> | 07-Aug-19 |
| A264 | Postobon | Twitter | Discursive strategy | Frame the debate | Postobón empresa @postobonoficial Follow Follow @postobonoficial Realizar actividad física produce beneficios para la salud; ayuda a dormir mejor, facilita la movilidad y ayuda a tener una vida saludable. #TómateLaVida dedicando unos minutos para hacer actividad física y mejorar tus hábitos. #Salud #ActividadFísica☝ 4:00 AM - 22 Feb 2019 |  | <https://twitter.com/postobonoficial/status/1098915312865501184> | 07-Aug-19 |
| A265 | Postobon | Twitter | Discursive strategy | Frame the debate | Postobón empresa+F115  @postobonoficial  Feb 23 More ¡Actívate! No importa dónde estés, mueve tu cuerpo, sal de la rutina. Libérate de tensiones, cansancio y estrés. En nuestro blog #TómateLaVida te contamos cómo puedes hacerlo. |  | <https://twitter.com/postobonoficial/status/1099296316067323908> | 07-Aug-19 |
| A266 | Postobon | Twitter | Discursive strategy | Frame the debate | Postobón empresa ‏@postobonoficial  Feb 21 More En Postobón te invitamos a que tengas un estilo de vida activo, creando nuevos hábitos que te mantengan sano. #TómateLaVida🍃🍃🍃 |  | <https://twitter.com/postobonoficial/status/1098552917471170561> | 07-Aug-19 |
| A267 | Postobon | Twitter | Discursive strategy | Frame the debate | Postobón empresa @postobonoficial Follow Follow @postobonoficial ¡No hay excusas para hacer ejercicio! ¿sabías que puedes hacerlo en casa? Mira nuestro post en Tómate La Vida y aprende cómo puedes comenzar a ejercitarte sin tener que ir al gimnasio. #TómateLaVida 😃 👍 ✊ 💪  https://bit.ly/2VJDXjb  3:50 AM - 22 Jan 2019 |  | <https://twitter.com/postobonoficial/status/1087678829613719553> | 07-Aug-19 |
| A268 | Postobon | Twitter | Discursive strategy | Frame the debate | Postobón empresa @postobonoficial  20 janv. Después de unas vacaciones emocionantes se acerca el regreso a clases, y queremos que tus hijos alimenten de una manera saludable en el colegio. Aquí te mostramos opciones que puedes implementar para cada día de la semana.🙋‍♀🙋‍♂ #TómateLaVida |  | <https://twitter.com/postobonoficial/status/1087122600688373760> | 08-Aug-19 |
| A269 | Postobon | Twitter | Discursive strategy | Frame the debate | Postobón empresa @postobonoficial  18 janv. En Postobón estamos comprometidos con la innovación; está en nuestro ADN. En este video te contamos de qué manera innovamos con nuestras bebidas y cómo estas pueden aportar a tu bienestar. #NuestroCompromiso 👍 🇨🇴 |  | <https://twitter.com/postobonoficial/status/1086292133223362560> | 08-Aug-19 |
| A270 | Postobon | Twitter | Discursive strategy | Frame the debate | Postobón empresa @postobonoficial  11 janv. En Postobón nos tomamos la vida enseñándote a crear hábitos saludables para que aprendas a disfrutar de una vida activa. ☝ ✍ En el nuevo post de #TómateLaVida te contamos cómo hacerlo. ¿Tienes alguna rutina de ejercicios? Compártela con nosotros en... |  | <https://twitter.com/postobonoficial/status/1083785648526512134> | 08-Aug-19 |
| A271 | Postobon | Twitter | Discursive strategy | Frame the debate | Postobón empresa @postobonoficial Mantener un ritmo de vida saludable es importante para tu bienestar. ¡Recuerda crear hábitos responsables para ti y tu familia! Aquí te dejamos algunos consejos que puedes comenzar a poner en práctica. #TómateLaVida 05:04 - 4 janv. 2019 |  | <https://twitter.com/postobonoficial/status/1081174406511185920> | 08-Aug-19 |
| A272 | Postobon | Twitter | Discursive strategy | Frame the debate | Postobón empresa @postobonoficial  8 janv. #TómateLaVida en este nuevo año. Comienza el 2019 con una vida activa. 🙋‍♀ 🙋‍♂ ¿Quieres saber cómo? Haz clic en el enlace y te contamos 😃 #VidaActiva #Salud #EstiloDeVida |  | <https://twitter.com/postobonoficial/status/1082675800631988224> | 08-Aug-19 |
| A273 | Postobon | Industry website | Discursive strategy | Frame the debate | Nos autorregulamos • No comercializamos bebidas azucaradas en colegios ni escuelas primarias. • Incorporamos el etiquetado frontal bajo el sistema GDA en todas las bebidas y educamos sobre lectura de información nutricional. • Innovamos en el portafolio de bebidas con alternativas que propicien decisiones conscientes de consumo. • No hacemos publicidad de bebidas dirigida a menores de 12 años. • Promocionamos los estilos de vida activa, saludables y responsables. 72% de nuestro portafolio es reducido, bajo o libre de calorías*. Brindamos múltiples opciones para distintos momentos, que pueden ir desde la indulgencia hasta el máximo cuidado. Nuestra meta es que en 2024 el 50% del volumen total de litros que vendamos sea sin azúcar añadida. del volumen de ventas lo generan bebidas diferentes a las gaseosas. 52% del volumen de ventas lo generan bebidas diferentes a las gaseosas. Somos más que gaseosas. Somos amplitud de opciones. Brindamos opciones para que los colombianos se tomen la vida (aguas, bebidas con fruta, tés, hidratantes, energizantes y cervezas). Lo hacemos porque entendemos las tendencias de consumo y damos respuesta a las necesidades de los grupos de interés.  100% del portafolio cumple con el etiquetado GDA** |  | <https://www.postobon.com/sites/default/files/resumen_informe_de_sostenibilidad_2018_0.pdf> | 19-Aug-19 |
| A274 | Postobon | Twitter | Information management Discursive strategy | Amplification Frame the debate | Postobón empresa @postobonoficial  15 janv. Cuidar la alimentación en el trabajo también es Tomarse la vida. Hoy nuestro post de #TómateLaVida te cuenta cómo tener una dieta equilibrada y algunos consejos para mantener una alimentación saludable. 😃 👍 ✊ 💪 https://bit.ly/2ApB8e4 |  | <https://twitter.com/postobonoficial/status/1085168521892581377> | 08-Aug-19 |
| A275 | Postobon | Industry website | Coalition management Information management Discursive strategy | Community Amplification Frame the debate | Con el fin de promover hábitos de vida responsables en jóvenes entre los 12 y 17 años, la Fundación Postobón, con el apoyo de la Secretaría de Educación de Medellín, lanzó en Medellín, el programa ¡Boom! Activa tu vida, un programa que promueve la realización de actividad física en 60 instituciones educativas oficiales y privadas de la ciudad, para beneficio de cerca de 9.000 jóvenes. El objetivo del programa es promover en los estudiantes el gusto por la actividad física y dejar a un lado las barreras que impiden su realización y su metodología, ofrece un acompañamiento integral a las instituciones educativas beneficiadas para que tengan mayores elementos y argumentos que motiven y sensibilicen a los jóvenes, docentes y familias sobre los beneficios de la actividad física, además de brindar herramientas técnicas y pedagógicas para implementar buenas prácticas, luego de la jornada académica. De igual forma, el programa contempla la entrega de materiales deportivos que acompañen los procesos formativos relacionados con la actividad física en los estudiantes. 60 Instituciones a intervenir en seis zonas de la ciudad 9.000 Estudiantes beneficiarios de los grados 6to a 11 BENEFICIOS DE LA ACTIVIDAD FÍSICA Mejora el estado muscular y cardiorrespiratorio, la salud ósea y funcional. Reduce el riesgo de hipertensión, cardiopatía coronaria, accidente cardiovascular, diabetes, diferentes tipos de cáncer y la depresión. Ayuda al control de peso y mitiga enfermedades no transmitibles. Así se ve ¡Boom! Activa tu vida Estas son algunas de las piezas de comunicaciones que acompañan el programa ¡Boom! Activa tu vida, las cuales buscan la motivación de las personas para que tomen decisiones conscientes al respecto de la actividad física. | See more information here: https://tomatelavida.com.co/por-que-el-programa/ and CSR report: https://www.postobon.com/sites/default/files/resumen_informe_de_sostenibilidad_2018_0.pdf | <https://fundacionpostobon.com/programas/boom-activa-tu-vida/> | 19-Aug-19 |
