# Supplementary material for: “The architecture of the state was transformed in favour of the interests of companies”: corporate political activity of the food industry in Colombia
Source: Global Health. 2020 Oct 12;16:97. doi: 10.1186/s12992-020-00631-x (PMC7552360; doi:10.1186/s12992-020-00631-x)
Supplement: Supplementary file 5 — Additional file 5. Interview guide (Spanish). [file 12992_2020_631_MOESM5_ESM.docx]

Additional file 5: Interview guide (Spanish)

**Introducción (inicio de cada entrevista):**

Gracias por aceptar participar en este proyecto de investigación. Sé que su tiempo es extremadamente precioso, y gracias por usarlo para contribuir con este importante proyecto.

Como mencioné en mi carta de invitación, el objetivo de esta investigación es comprender las prácticas de la industria de alimentos que pueden influenciar la prevención y el control de las enfermedades crónicas no transmisibles relacionadas con la alimentación. El contexto es que el poder y la influencia de la industria de alimentos se han identificado como un factor importante que influye en las políticas de salud pública y la opinión pública en todo el mundo. Nuestro objetivo es identificar y monitorear esas prácticas, como parte de los esfuerzos para prevenir y controlar las enfermedades crónicas no transmisibles relacionadas con la alimentación. Usted ha sido seleccionado debido a su amplia experiencia de primera mano, observando y / o interactuando con la industria de alimentos, y creemos que puede proporcionar una visión crítica de las prácticas de la industria de alimentos. Hablaré con una serie de expertos, como usted, para obtener una visión detallada de esta área.

Espero que la entrevista lleve unos 45 minutos a 1 hora. Si usted está de acuerdo, escribiré la entrevista para cerciorarse de que registro sus puntos de vista con precisión, pero tenga el 100% de garantía de que toda la información proporcionada en la entrevista no será vinculada a su nombre y no será identificable como proporcionada por usted.

Firmar el formulario de consentimiento (entrevista + grabación digital)

*Perguntas-chave Prompts (se necessário)*

- ¿Para empezar, usted podría decirme de qué manera interactuó con la industria alimenticia, como parte de su profesión?

*Trabajo actual, trabajos anteriores, reuniones, alianzas, fondos*

- Y, específicamente, en qué roles profesionales y en qué situaciones interactuaste y observó la industria de alimentos?
- Como he mencionado, ciertas prácticas de la industria alimentaria están potencialmente proyectadas para influir en la política de salud pública. ¿Cuáles prácticas ha observado al respecto?

*exemplos*

- Ahora voy a presentar una lista de prácticas de la industria de alimentos que fueron previamente identificadas como potencialmente influyentes en la prevención y control de las Enfermedades Crónicas No Transmisibles relacionadas a la alimentación. Para cada una de las prácticas, usted puede indicar si usted la experimentó o observó?
- ¿Podrías decirme cuáles de estas prácticas crees que pueden representar un riesgo para las políticas y resultados de salud pública y por qué?
- ¿Podría indicar cuáles de estas prácticas creen más influyentes en las políticas y los resultados de la salud pública? Y cuáles son los menos influyentes
- Por último, nos gustaría entrevistar a otras personas que puedan proporcionar información sobre esta área. ¿Quién más usted sugiere que entrevistamos sobre eso?

*por ejemplo. ex políticos, ex-personas de la industria alimentaria, otras personas que tienen una vasta experiencia en esta área*

*Gracias de nuevo por su importante visión sobre esta área y por ofrecer su tiempo para ayudar en este proyecto de investigación. Explique cómo voy a comunicar los resultados con ellos y los próximos pasos.*
