# Supplementary material for: “The architecture of the state was transformed in favour of the interests of companies”: corporate political activity of the food industry in Colombia
Source: Global Health. 2020 Oct 12;16:97. doi: 10.1186/s12992-020-00631-x (PMC7552360; doi:10.1186/s12992-020-00631-x)
Supplement: Supplementary file 6 — Additional file 6. Spanish version of the article. [file 12992_2020_631_MOESM6_ESM.docx]

Additional file 6: Spanish version of the article

**Título:** “La arquitectura del Estado se transformó a favor de los intereses de las empresas”: actividad política corporativa de la industria alimentaria en Colombia.

**Autores**

Melissa Mialon ^a,b^ *, Diego Alejandro Gaitan Charry ^b^, Gustavo Cediel ^b^, Eric Crosbie ^c^, Fernanda Baeza Scagliusi ^a^, Eliana María Pérez Tamayo ^b^.

**Afiliaciones**

a Escuela de Salud Pública, Universidad de São Paulo, Brasil.

b Escuela de Nutrición y Dietética, Universidad de Antioquia, Medellín, Colombia.

c Escuela de Ciencias de la Salud Comunitaria, Universidad de Nevada, Reno, EE. UU.

*Autor para correspondencia

**Antecedentes:** En Colombia, se están desarrollando políticas de salud pública para mejorar los entornos alimentarios y entre ellas se ha incluido el etiquetado frontal de alimentos y las restricciones de comercialización de productos no saludables. Los actores de la industria alimentaria que se oponen a estas políticas están retrasando y debilitando estos esfuerzos. Esta oposición se conoce comúnmente como "Actividad Política Corporativa" (CPA) e incluye estrategias instrumentales (basadas en la acción) y estrategias discursivas (basadas en argumentos). El objetivo de este estudio fue identificar la APC de la industria alimentaria en Colombia.

**Métodos:** Se hizo un análisis documental de la información disponible públicamente que fue publicada entre los meses de enero y julio de 2019. Estos datos se triangularon con entrevistas desarrolladas con diecisiete informantes clave. Se empleo un enfoque deductivo para el análisis de datos, basado en un marco existente para el estudio de la APC de la industria alimentaria.

**Resultados:** Se identificaron 275 ocurrencias de APC a través del análisis de información disponible públicamente. Se reconocieron 197 ejemplos de estrategias instrumentales y 138 ejemplos de estrategias discursivas (estas categorías no son mutuamente excluyentes, 60 ejemplos pertenecen a ambas categorías). Los informantes clave durante las entrevistas también compartieron información sobre la APC en el país. La industria utilizó sus estrategias discursivas para hacer una fotografía de sus prácticas bajo una "mejor luz", demostrando sus esfuerzos por mejorar los entornos alimentarios y su papel en el desarrollo económico del país. La industria alimentaria participó en varios programas comunitarios, incluso a través de iniciativas público-privadas. La industria también capturó a los medios de comunicación e intentó influir en la ciencia sobre la nutrición y las enfermedades no transmisibles. Los actores de la industria alimentaria fueron muy prominentes en la esfera de las políticas públicas, a través de su cabildeo, en este sentido fueron evidentes sus estrechas relaciones con funcionarios de alto rango y su apoyo a la autorregulación en el país.

**Conclusiones:** La proximidad entre la industria, el gobierno y los medios de comunicación es particularmente evidente e incuestionable en Colombia. La influencia sobre las poblaciones vulnerables en las comunidades y la sensación de inseguridad de los defensores de la salud pública también es preocupante. En Colombia, la APC de la industria alimentaria, tiene el potencial de debilitar y retrasar los esfuerzos para desarrollar e implementar políticas de salud pública que podrían mejorar la salubridad de los entornos alimentarios. Es urgente que se desarrollen en el país mecanismos para prevenir y gestionar la influencia de la industria alimentaria.

**Palabras clave**

Determinantes comerciales de la salud; actividad política corporativa; industria de alimentos; enfermedades no transmisibles.

**Antecedentes**

En el año 2019 en Colombia, las políticas de salud pública para mejorar los entornos alimentarios, incluyó la introducción de un nuevo sistema de etiquetado nutricional frontal de los alimentos y las restricciones de comercialización para productos no saludables, las cuales fueron debatidas en el Congreso (1-3). Estas acciones de política pública se generaron como respuesta al aumento de la carga de enfermedades no transmisibles (ENT), como la diabetes, las enfermedades cardiovasculares y el cáncer, que ahora son responsables del 75,00% de todas las muertes en el país (4). Las dietas poco saludables, en particular, se encuentran entre los principales factores de riesgo de las ENT (5). Según la última Encuesta Nacional de Situación Nutricional de Colombia realizada en el año 2015, entre los niños menores de 5 años, el 10,8% presentaba retraso en el crecimiento, mientras que el 6,4% tenía sobrepeso (6).

Las organizaciones de la sociedad civil y los medios de comunicación informaron que los actores que representan a la industria alimentaria se opusieron firmemente a estas políticas (7–13). La influencia de la industria alimentaria en los medios de comunicación y en el Congreso, se hizo evidente en el 2016 durante la propuesta de aumento de impuestos a las bebidas azucaradas, que aún no se ha implementado en Colombia (11). En este caso, la industria encargó sus propios estudios económicos para contrarrestar la evidencia de que se necesitaba un aumento en el impuesto para mejorar la salud de la población (11). Los actores de la industria alimentaria también desarrollaron varias iniciativas de "responsabilidad social empresarial" en el país, a través del apoyo de las comunidades, que podrían haber contribuido a mejorar su imagen en la opinión pública (11). La industria ha continuado ejerciendo su influencia, utilizando prácticas similares, durante el desarrollo de la Ley de Prevención de la Obesidad N ° 019 de 2017 (Proyecto de Ley o PL019 de 2017) que incluyó el desarrollo de un nuevo sistema de etiquetado nutricional frontal de los alimentos y restricciones de la comercialización en alimentos poco saludables para los niños (10,13,14). Un informe de investigación describió el uso de la "puerta giratoria" con empleados de la industria alimentaria que van a trabajar en el gobierno (15).

Estas acciones de la industria alimentaria representan la denominada “actividad política corporativa” (APC), que incluye estrategias instrumentales basadas en la acción (gestión de coaliciones; gestión de la información; participación e influencia directa en las políticas; estrategias legales) y estrategias discursivas basadas en argumentos, haciendo hincapié en la alimentación, destacando por ejemplo, importancia de la industria en la economía, los costos potenciales asociados con la implementación de políticas de salud pública y el fomento del debate sobre los problemas de salud relacionados con la dieta de manera favorable a sus productos y prácticas, con énfasis en la responsabilidad de las personas y la libertad de elección que estos poseen frente a su alimentación (16, 17). Estas prácticas se describen en el Archivo 1. Los académicos explican que la APC no es necesariamente puntual y está sujeta a períodos de tiempo específicos, como durante el desarrollo de políticas específicas que podrían amenazar las actividades de una industria, sino que se utiliza para influir en la salud pública tanto a corto como a largo plazo (18). La APC es parte de una literatura más amplia sobre los determinantes comerciales de la salud, lo que corresponde a la influencia negativa que tienen las corporaciones sobre la salud (19-21).

Existe una investigación limitada y falta de seguimiento de la APC de la industria alimentaria en América Latina (22), incluida Colombia. Un estudio piloto en la región mostró que, en el país, los actores de la industria alimentaria enfatizaron su papel destacado en la economía para contrarrestar las críticas; intentaron demostrar que eran parte de la solución en la prevención y control de las ENT; y construyeron alianzas con organizaciones y comunidades de salud pública (23).

En el presente estudio, el objetivo general fue precisamente identificar la APC de la industria alimentaria en Colombia.

**Métodos**

Se realizó un análisis documental de información disponible públicamente triangulado con entrevistas, desarrollado entre mayo y agosto de 2019. El estudio fue dirigido por un investigador internacional con experiencia en la APC de la industria alimentaria, se hizo en Colombia y la recolección y análisis de datos fue en inglés y español. Algunos de los entrevistados conocían a la investigadora por su trabajo en ese espacio, pero no personalmente. El equipo de investigación también estuvo integrado por tres investigadores locales con experiencia en políticas públicas alimentarias y nutricionales y entornos alimentarios en Colombia y a nivel mundial, participaron dos investigadores internacionales, con experiencia en entornos alimentarios y prácticas políticas de la industria. Todos los investigadores de este proyecto adoptaron una postura crítica sobre la influencia de las corporaciones en las políticas de salud pública.

En el estudio la “industria alimentaria” incluyó a los fabricantes de productos alimenticios y bebidas, mayoristas, minoristas, distribuidores, proveedores de servicios alimenticios y productores de materias primas, así como a organizaciones que actuaban en su nombre, de manera abierta o encubierta, incluidas asociaciones comerciales, empresas públicas. firmas de relaciones, organizaciones “filantrópicas”, instituciones de investigación y otras personas y grupos.

Este manuscrito cumple con los criterios de COnsolidated for Reporting Qualitative Research (COREQ) (24) [Archivo 2].

**Análisis de documentos**

Para el análisis de documentos, se utilizó un protocolo desarrollado por INFORMAS (por su sigla en inglés, Red Internacional de Investigación, Monitoreo y Apoyo a la Acción en Alimentos y Obesidad / Enfermedades no Transmisibles) para identificar la APC de la industria alimentaria (16). Estos métodos y el marco utilizado para el análisis de datos se han aplicado en diferentes países del Pacífico, Europa y América Latina (23,25-30).

La recopilación y el análisis de datos estuvo a cargo del primer autor, utilizando Excel para administrar los datos recolectados.

Desde INFORMAS se sugiere identificar a los actores más destacados en un determinado país, en términos de cuotas de mercado (16). No se tuvo acceso a esta información para la industria alimentaria colombiana y, en cambio, se efectuó una consulta con expertos locales y se hizo un estudio piloto, según lo recomendado por INFORMAS en estas circunstancias (16): se visitaron las páginas web de dos fabricantes globales que tenían sitios web nacionales, Nestlé y Coca Cola. Esto ayudó a estimar el nivel de información disponible en estas páginas web. Con base en ese análisis, se decidió incluir en el estudio veinte actores de la industria alimentaria y datos publicados entre enero y julio de 2019 (muestra intencionada), excepto para el informe anual u otro evento anual, donde se incluyeron los datos más recientes. Los actores de la industria analizados se presentan en la Tabla 1. Se consideraron los miembros de la Alianza Internacional de Alimentos y Bebidas (IFBA, por su sigla en inglés), ya que estos se encuentran entre los mayores fabricantes de alimentos y bebidas a nivel mundial (31). Otros actores de la muestra fueron productores locales de alimentos y bebidas, un minorista y tres grupos financiados por la industria alimentaria.

Todos los datos enunciados están disponibles como Archivo 3.

Según lo recomendado por INFORMAS (16), las fuentes de información para el estudio circunscribieron material propio de la industria, material gubernamental y datos de otras fuentes, incluidas asociaciones profesionales y universidades. Las fuentes consultadas para el estudio se presentan como Archivo 4. Cabe anotar que empresas como Mars, General Mills, Grupo Bimbo y Unilever no tenían sitio web nacional ni cuenta Twitter.

El análisis de datos se describe en el protocolo INFORMAS y consistió en la identificación y codificación simultánea de datos relevantes para la APC, utilizando un marco existente [Archivo 1] (16). El tercer y cuarto autores revisaron el 10,0% y el 100% de los datos, respectivamente. El desacuerdo se resolvió mediante discusión (este no fue un proceso de cuantificación sino de análisis de argumentos).

Este manuscrito presenta las diferentes estrategias de APC de la industria alimentaria, como se indica en el análisis de documentos y entrevistas. Para estas entrevistas e informantes clave se asignó un código que comienza con la letra A seguida de un número a cada ejemplo de APC identificado en el análisis de documentos.

**Entrevistas**

El objetivo de las entrevistas fue tener acceso a informantes clave que tenían una experiencia de primera mano en el APC de la industria alimentaria, sin límites de tiempo específicos, ni restricciones sobre el tipo de actores de la industria. Los ejemplos de APC compartidos por los participantes, ayudaron a triangular los datos encentrados en el espacio público. También se identificaron ejemplos adicionales, como se detalla en la sección de resultados. Además, durante las entrevistas, los participantes compartieron sus perspectivas y opiniones sobre la APC de la industria alimentaria en Colombia y a nivel mundial.

El primer autor realizó trece entrevistas semiestructuradas, incluidas dos entrevistas grupales. En total, diecisiete informantes clave participaron en el estudio, del poder legislativo del gobierno (n = 1), el poder ejecutivo del gobierno (n = 1), la academia (n = 1), la sociedad civil (n = 12), y los medios de comunicación (n = 2). Una persona de la academia aceptó la invitación al estudio, pero estaba viajando y, por lo tanto, no pudo ser entrevistada. Las entrevistas se efectuaron hasta alcanzar la saturación de los datos (es decir, cuando el primer autor no identificó ningún tema nuevo / prácticas de APC). El muestreo fue intencional y los participantes se identificaron a través de su discusión sobre la APC de la industria alimentaria en Colombia en los medios de comunicación. También se empleó una técnica de muestreo de bola de nieve (los participantes invitaron a posibles entrevistados de sus redes sociales). La guía de entrevistas está disponible como Archivo 5.

Los participantes fueron contactados por correo electrónico o llamadas telefónicas y se les ofreció participar en el estudio, voluntariamente y bajo estrictas condiciones de anonimato y confidencialidad. Se firmó un acuerdo ético entre el entrevistador y los participantes. Los participantes dieron su consentimiento con la toma de notas de campo y la grabación digital de la entrevista. Estos tuvieron la oportunidad de revisar su transcripción antes de la presentación de este manuscrito. En esta etapa, una participante pidió que se eliminara la mayor parte de la información compartida durante su entrevista por temor a represalias. Un participante se retiró del estudio en la etapa de revisión por pares durante la publicación del presente artículo, luego de que se publicaran otros artículos sobre el APC de la industria alimentaria en Colombia. Por lo tanto, no se contó en la lista de participantes.

Las entrevistas duraron en promedio una hora; se realizaron cara a cara (n = 12) o a través de Skype (n = 1); en español (n = 10), español / inglés (n = 3) y francés (n= 1). Las entrevistas fueron transcritas palabra por palabra por un traductor contratado bajo condición de confidencialidad.

El análisis de datos fue dirigido por el primer autor y este utilizó el marco existente presentado en el Archivo 1 descrito previamente en este documento. El segundo y último autor revisaron el 10,00% y el 100% de los datos para las entrevistas, respectivamente. Se emplearon los programas Word y Excel para administrar los datos.

Toda la información que pudiera identificar a los participantes se eliminó de este manuscrito y se utilizan términos genéricos para describir sus profesiones, sin asignar un número a cada participante, para preservar su anonimato y confidencialidad. En este sentido se emplearon los pronombres "ella / el" cuando se hace referencia a participantes masculinos o femeninos.

**Resultados**

Se identificaron 275 ocurrencias de APC entre enero y julio de 2019 a través del análisis de información disponible públicamente. La Tabla 2 presenta un resumen de los ejemplos encontrados en el dominio público, clasificados por actor de la industria y por estrategia de APC.

Asimismo, se identificaron 197 ejemplos de estrategias instrumentales y 138 ejemplos de estrategias discursivas. Estas categorías no son mutuamente excluyentes y, por lo tanto, 60 ocurrencias pertenecen a ambas estrategias de APC Los participantes de las entrevistas también identificaron ejemplos de APC, describiendo acciones o argumentos que han sido utilizados en los últimos años por la industria alimentaria en Colombia. Cabe anotar que las estrategias de APC esgrimidas por la industria alimentaria con respecto a la discusión sobre la introducción de un nuevo sistema de etiquetado frontal de alimentos en Colombia es un tema de una publicación separada (32).

**Gestión de coaliciones: construyendo alianzas y debilitando a la oposición**

En Colombia, a partir del análisis de documentos se identificaron 101 ejemplos de la estrategia de gestión de coaliciones. Incluso, durante las entrevistas se compartieron ejemplos adicionales. Como parte de esta estrategia, la industria alimentaria estableció relaciones con organizaciones de salud, comunidades y medios de comunicación y con otros actores de la industria. Paralelamente, utilizó diferentes mecanismos para debilitar a sus oponentes, como se describe a continuación.

**Captura de los medios de comunicación**

Los entrevistados destacaron la captación de los medios de comunicación en Colombia, donde, por ejemplo, el Grupo Ardila Lulle es dueño de un canal de televisión líder en el país, llamado RCN, y además de la empresa de bebidas Postobón. Esta apropiación dio lugar a dos casos de censura de campañas de salud pública en Colombia, como se describe en la sección de "estrategias legales", a continuación.

*“RCN [un canal de televisión] pertenece a un grupito económico que se llama organización Ardila Lule. Y la organización Ardila Lule tiene la empresa de gaseosas más grandes de Colombia, que se llama Postobón. (...) los medios dominantes en Colombia todos tiene relación con la industria alimentaria, es decir relación directa de propiedad, la industria alimentaria es dueña de los medios, esa es la palabra”. [defensor de la salud pública]*

*“El periódico, la radio, la televisión más importante son los medios comprados por grupos de industriales que tienen una gran cantidad de empresas en el agronegocio. Esta es una de las razones por las cuales los anuncios [de salud pública] en la televisión y la radio fueron inmediatamente censurados (...) Refleja perfectamente que los medios pertenecen a los grupos económicos en los que también se encuentra la agroindustria”. [defensor de la salud pública]*

**Interacciones con la sociedad civil y las organizaciones de salud y participación en la comunidad**

Varias empresas tienen sus propias organizaciones benéficas en el país. La Fundación Éxito colaboró con distintos actores de la industria alimentaria, entre ellos Coca-Cola, y con los ayuntamientos, el Ministerio de Salud, el Instituto Colombiano de Bienestar Familiar (Instituto Colombiano de Bienestar Familiar), el Departamento de Planificación Nacional y la Oficina de la Inspector General de Colombia [A84]. La Fundación Nutresa contó con el apoyo del Ministerio de Educación de Colombia, UNICEF y el Programa Mundial de Alimentos [A217]. Un participante en nuestras entrevistas también describió un programa gubernamental que involucró a empleados de la industria alimentaria:

*“Hay un programa [organizado por el Instituto Colombiano de Bienestar Familiar o Instituto Colombiano de Bienestar Familiar, ICFBF] de apoyo alimentario y educativo a las familias con niños menores de dos años que se llama “La modalidad familiar” (…) Estos programas deben contratar profesionales para atender los niños. En algunas ocasiones las capacitaciones a los profesionales las hace Nestlé o Alpina. Las capacitaciones oficiales del ICBF. Entonces es una cosa de no creer”. [defensor de la salud pública]*

La Tabla 3 presenta otras iniciativas que fueron financiadas o apoyadas por la industria alimentaria en Colombia durante el período de análisis.

La información contenida en la Tabla 3 demuestra hasta qué punto la industria alimentaria está presente en las comunidades de Colombia. También muestra las numerosas interacciones entre los actores de la industria alimentaria y el sector público. Además, la mayoría de los programas comunitarios patrocinados o apoyados por la industria se dirigían a los niños y se centraban en la educación, la nutrición y / o la actividad física.

Varios de los participantes entrevistados describieron un caso, del año 2017, donde Postobón lanzó un programa en una región desértica del extremo norte de Colombia, en el departamento de la Guajira, donde la empresa distribuía, diariamente, dos bebidas fortificadas con micronutrientes a niños (33). Los entrevistados explicaron que la industria planeaba comercializar estas bebidas en el resto del país y dijeron que Postobón inició un estudio con estos niños, sin aprobación de un comité de ética independiente, tomando muestras de sangre y medidas antropométricas (33). Los participantes de este estudio también discutieron sobre los esfuerzos de la industria para obtener el apoyo del Ministerio de Salud para este programa, lo que nunca sucedió. Esta historia se convirtió en un escándalo en los medios a principios del año 2018, pero una investigación reciente encontró que, casi dos años después, Postobón sigue ejecutando su programa en diferentes comunidades de Colombia (33,34).

*“Cuando empieza a salir el escándalo, ellos ya empiezan a cambiar el discurso, como te dije, y dicen que ellos nunca tienen interés comercial [en desarrollar estos productos]. Y al final nunca salió al mercado y lo retiraron de los programas de las escuelas”. [miembro del gobierno]*

Estas interacciones podrían ser perjudiciales para la salud pública, particularmente cuando los programas tienen una marca fuerte o cuando la industria distribuye productos que podrían no ser saludables. Por ejemplo, Colanta, Nestlé, Nutresa y Postobón utilizaron material de marketing con sus marcas al organizar eventos en las escuelas y / o la comunidad [A58-9, A185, A193, A216, A241]. Además, Colanta a través del “Programa Maná”, del gobierno del Estado de Antioquia, distribuyó “una ración diaria de leche en polvo aromatizada, como caramelo de complemento nutricional, que puede ser consumida directamente o diluida en agua” a 140.000 niños [A60]. Nuestros participantes criticaron estos programas comunitarios:

*“[Las escuelas] están recibiendo fondos y están validando la llegada de la industria en los entornos escolares, los cuales deberían estar protegidos de, justamente las marcas, y deberían estar protegidos de la disponibilidad de esos productos (…) de hecho es posicionamiento de marca lo que están haciendo". [defensor de la salud pública]*

*“Eso es super malo, porque entonces [los actores de la industria alimentaria] están llegando a las poblaciones más vulnerables, tratando de ser de cierta forma como el salvador. Y eso de hecho le da una potestad de tener a su favor a población vulnerable que puede defender los intereses directamente a la industria. Y aún más llegando con el Estado, [que] los valida con mayor fuerza”. [defensor de la salud pública]*

Finalmente, estos programas podrían ayudar a la industria a obtener un acceso privilegiado a los responsables políticos:

*“Lo que hacen con todos estos programas en varios territorios básicamente es crear alianzas estratégicas con actores locales de la sociedad civil y tomadores de decisión que finalmente va creando una base de respaldo para ellos”. [defensor de la salud pública]*

Es fundamental señalar que Colombia es un caso único, en el sentido de que tiene una historia que ha estado marcada por un conflicto armado (35). Además, muchos segmentos de su población, incluidos los indígenas y los afrodescendientes, siguen marginados y carecen de acceso a la infraestructura básica, la alimentación y la educación (36). Como tal, la información publicada en el dominio público (Tabla 3) y compartida por los participantes sugiere que la participación de la industria alimentaria en la comunidad a menudo se considera una contribución a la paz, la alegría, el desarrollo social y la prosperidad del país (esto también se describe en la sección “estrategias discursivas” a continuación). La industria a veces llena un vacío donde el gobierno ha estado ausente.

*“Unos niños que antes no iban al colegio y ahora van al colegio, ¿que vas a decir?; ¿pues esto está mal? ¿Es mejor que no vayan al colegio? Pero entonces es un vacío del Estado. El Estado esta out-sourcing a la industria privada una serie de servicios que son responsabilidad de ellos entonces ahí es donde está el problema. (…) Entonces usas a la industria para hacer como obras de caridad, ¿no?" [periodista]*

Sin embargo, esta posición fue criticada por algunos de nuestros participantes:

*“El gobierno no tiene dinero, simplemente porque el gobierno que fue infiltrado por las empresas no le cobra a las empresas ni los impuestos, no le cobra tazas de ganancia (…). El gobierno instituyó amplias zonas francas en el país, donde ellos pueden importar su materia prima sin ningún costo de arancel. Por supuesto, el Estado no tiene dinero (…) Pues porque toda la arquitectura del Estado fue transformada en favor de los interese de las empresas”. [defensor de la salud pública]*

**Fragmentación y desestabilización de la circunscripción**

Paradójicamente, la defensa de los derechos humanos a un nivel de vida adecuado, incluido el derecho humano a la alimentación, y la promoción de la prevención y el control de las ENT, a menudo exponen a las personas, en particular a las de la sociedad civil, a amenazas y peligros en Colombia. Así lo describió un artículo del New York Times en 2017 (37), cuando la dirección de la organización de consumidores EducarConsumidores recibió amenazas directas, aunque en ese momento no se establecieron vínculos directos con la industria alimentaria. Los actores de la salud pública en Colombia se sentían inseguros a diario. Incluso a algunos les robaron el equipo, incluido material con información confidencial.

*“[Nos sentimos] inseguros (…) si, debemos, porque nos hemos dado cuenta que hay muchas personas (…) que se hacen detrás de cada persona a ver que escriben en el celular (…) cuando son las plenarias en los salones son grandes, nos hemos dado cuenta que llevan fotógrafos con cámaras con un lente [tan pequeño] sin mentirte. Sin exagerarte”. [defensor de la salud pública]*

**Gestión de la información: influir en la ciencia**

La industria alimentaria utilizó diferentes prácticas para intentar incidir en la producción y difusión de información sobre salud pública nutricional en Colombia. Encontramos 99 ejemplos en esta categoría en nuestro análisis de documentos. Esta estrategia también se discutió durante las entrevistas.

**Producción propia y ampliación de la investigación**

En Colombia, actores de la industria alimentaria realizaron directamente investigaciones y difundieron información sobre nutrición. Nutresa tenía su propio centro de investigación sobre enfermedades no transmisibles y sus vínculos con las dietas, llamado Vidarium (donde “Vida” significa vida) [A226, A228]. Nestlé ejecutó su programa de nutrición en Colombia, “Unidos por Niños Saludables” (ver Tabla 3), donde difundió información a niños, padres y maestros [A201, A204-5]. La empresa colaboró con la Facultad de Enfermería y Rehabilitación de la Universidad de La Sabana para la validación de este programa [A188]. Los resultados del estudio sirvieron para promover aún más el programa [A188]. La Fundación Éxito entregó un Premio de Nutrición Infantil a “instituciones públicas y privadas de diferentes sectores [que] actúan para mejorar la nutrición de los niños en sus primeros 1000 días de vida” [A112]. Además, la Fundación Éxito organizó un evento en mayo del año 2019 donde se reunió con “algunos de los medios más medulares del país hablando de la importancia de la nutrición para el desarrollo del cerebro” [A100]. Coca-Cola organizó una serie de charlas “dirigidas a entidades gubernamentales, en las que brindamos información sobre el balance energético y la hidratación adecuada, contribuyendo a la promoción de estilos de vida activos y saludables. Hasta la fecha, hemos beneficiado a más de 900 personas”. [A55].

La “Alianza por la Nutrición Infantil”, una iniciativa público privada (ver Tabla 3) lanzada en el 2019 por la Fundación Éxito, organizada, en alianza con el Ministerio de Salud, ofreció diferentes cursos a profesionales de la salud sobre alimentación y epidemiología de lactantes y niños pequeños, donde dichos profesionales recibieron una certificación oficial del gobierno [A96-7].

Un participante de las entrevistas explicó que la industria también suele pagar los viajes y las tarifas de los estudiantes y académicos para asistir a estas conferencias [académicas]. Otro participante explicó que asociaciones profesionales, como la Asociación Colombiana de Dietistas y Nutricionistas (Asociación Colombiana de Dietistas y Nutricionistas, ACODIN), invitan a representantes de la industria alimentaria en sus congresos:

*“La intervención magistral inaugural del del [congreso de ACODIN hace unos años] fue de Jairo Romero [de la “Asociación Latinoamericana de Ciencia y Tecnología de Alimentos”, ALACCTA]. (…) Obviamente el evento estaba full de stands de la industria, ¿no?. (…) ACODIN, es la asociación de nutricionistas, está completamente cooptada por la industria”. [defensor de la salud pública]*

**ILSI Nor-Andino**

El ILSI Nor-Andino es la rama local del International Life Science Institute, un grupo de fachada de la industria que ha sido criticado por su influencia en la ciencia y la política en numerosos países (38–40). Alpina, Coca-Cola, Kellogg, Mondelez, Nestlé, Pepsico, Postobón y Unilever eran miembros de ILSI Nor-Andino en agosto de 2019 [A115]. En Colombia, un artículo de un periódico describió las muchas formas en que ILSI influye en las políticas y la investigación en el país (41). El ILSI colaboró con el Ministerio de Salud y académicos de diferentes universidades, sin revelar necesariamente sus vínculos con la industria alimentaria (41). Estas personas, a su vez, participaron en la formulación de políticas sin revelar estos vínculos con ILSI y la industria (41).

Entre los miembros del directorio de ILSI se encuentran un profesor jubilado de la Universidad Nacional de Colombia, un profesor de la Pontificia Universidad Javeriana y empleados de Nestlé y Alpina, entre otros [A114].

En las entrevistas, los participantes discutieron un proyecto de investigación sobre dieta y actividad física en América Latina, llamado "Estudio Latinoamericano de Nutrición y Salud" (Estudio Latinoamericano de Nutrición y Salud, ELANS). ELANS está financiado por Coca-Cola e ILSI, entre otros, y está liderado en Colombia por investigadores de la Pontificia Universidad Javeriana (42).

El ILSI y algunos de los miembros de su industria apoyaron diferentes eventos científicos para difundir información sobre nutrición en Colombia en 2019 [A120, A125]. El ILSI y Unilever patrocinaron el congreso anual de ACODIN [A160]. Unilever y Kellogg's patrocinaron algunas de las sesiones de ACODIN [A129, A161]. Asimismo, el ILSI, Danone y McDonald’s patrocinaron el congreso anual de la Asociación Colombiana de Nutrición Clínica (Asociación Colombiana de Nutrición Clínica, ACNC) [A162]. Durante el congreso, ILSI organizó una sesión sobre nutrición infantil [A162].

**La influencia en la ciencia se traduce en influencia política**

En las entrevistas, se sugirió que la influencia de la industria alimentaria en la ciencia en Colombia también podría traducirse directamente en influencia en política pública.

*“Uno de los (…) profesor[es] de Los Andes (…) ha trabajado mucho el tema de deporte pero con Coca-Cola (…) el ha saboteado varios [eventos públicos sobre nutrición y salud] (…). Es uno de los detractores académicos más fuertes que hay en Colombia. (…) Por ejemplo el nunca aparece en audiencias públicas, pero (…) es muy amigo del Ministro actual, si aparece en debates académicos (…). Y para él su conflicto de interés con Coca-Cola, no eso sí nunca lo menciona". [defensor de la salud pública]*

Los participantes de las entrevistas explicaron cómo la industria alimentaria trató de dar forma a la evidencia en Colombia durante la discusión sobre un aumento en los impuestos a las bebidas azucaradas entre los años 2016 y 2017:

*“Contrataron a dos personas para hacer dos estudios, dos personas muy nombradas en el país. (…). Y cada uno hizo un estudio por separado (…) y llegaron a decir en una exposición pública que si se ponía el impuesto a las bebidas azucaradas, las madres de familia y los padres de familia como sustituto [de las bebidas azucaradas] le iban a poner a los niños en la lonchera cerveza. (…) [Los estudios] nunca fueron revisados por pares, nunca los vimos publicados en una revista indexada, nunca dieron la publicación siquiera”. [Defensor de la salud pública]*

**Participación e influencia directa en la política pública**

La industria alimentaria es un actor destacado e influyente en las políticas de salud pública en Colombia. Se identificaron 16 ejemplos de esta práctica durante la recopilación de datos de documentos disponibles en el espacio público. Los participantes en las entrevistas también describieron ejemplos en esta categoría.

**Lobby o cabildeo**

Varios de los participantes entrevistados describieron el lobby o cabildeo que ejerce la industria alimentaria en el Congreso:

*“Logran cooptar a los nuevos parlamentarios que llegan (…) y lo que uno ve, es que empiezan a visitarlo, empiezan a pedir citas (…). Entonces allí, la industria, frecuentemente pedía citas para, pues hablar, manifestar sus intereses”. [político]*

*“Ellos ingresan al congreso y se meten a toda parte sin ninguna autorización legal; entonces se meten en la organización del orden del día de las plenarias, ayudan a romper el quórum de las plenarias, pasan proposiciones para que se las firme alguien para bloquear, para archivar proyectos de ley, para cambiar el articulado de los proyectos de ley. Ellos se meten en todo el proceso parlamentario de manera irregular”. [político]*

Un entrevistado describió cómo diferentes actores de la industria alimentaria unen fuerzas y construyen alianzas dentro de la industria para luego influir en las políticas en Colombia.

*“Una estrategia que utilizan es apalancarse en las agremiaciones (…). Entonces no son voces solitarias de una industria diciendo algo, sino que son voces agremiadas”. [actor de salud pública]*

El presidente de Colombia, entonces senador, presionó contra una propuesta para aumentar los impuestos a las bebidas azucaradas hace unos años cuando era senador [A141]. En el año 2019 participó y pronunció el discurso final en la asamblea de la sección de ANDI Bogotá, evento que dio a conocer en su sitio web oficial [A17]. Los participantes entrevistados sugirieron que estas interacciones entre el presidente y la industria tienen una influencia directa en la política del país:

*“Eso (…) se deduce de las posturas, por lo menos de este gobierno, de las posturas que tuvieron varios de los funcionarios que están hoy en el gobierno, empezando por el presidente Duque, que fue un defensor para que no se hiciera el impuesto a las bebidas azucaradas. Pues sin duda que los lobistas del gobierno van a estar en alianza con los lobistas de la industria". [político]*

**Donaciones y otros incentivos**

En cuento a la información sobre donaciones políticas es difícil de obtener datos disponibles públicamente en Colombia, ya que se necesitaría buscar información para cada individuo del gobierno para reconocer si ha recibido este tipo de donaciones, lo que posiblemente demandaría un estudio separado. Un participante resumió la situación en Colombia de la siguiente manera:

*“Hace muchos años (…) hablaba con un político y decía: "yo ya no quiero ser más político porque para ser congresista tienes o qué venderte a un grupo armado que te financie o a un grupo de empresarios. Entonces cada vez que vas a tomar una decisión siempre te mandan a alguien que te toca el hombro y te dice: “recuerde que X le mandó saludos, entonces usted y X quieren que esto no se vote, como sea que no se vote". [defensor de la salud pública]*

En un artículo de investigación se evidenció que la industria alimentaria realizó numerosas donaciones durante las últimas elecciones presidenciales del año 2018 (7). El presidente de Colombia, por ejemplo, declaró haber recibido el equivalente a US $ 148.000 de la industria de bebidas azucaradas durante su campaña electoral en 2018 (7).

Algunos entrevistados explicaron que la industria alimentaria también ofrece obsequios a los políticos:

*“Y de la otra forma es que la industria siempre visita para llevar presentes (...) ellos regalan objetos. Por ejemplo regalan lapiceros (…), llevan vinos, licores finos, dulces bien presentados, chocolates bien presentados”. [político]*

*“Las personas que iban de la industria al Congreso hablaban con los congresistas y les ofrecían: “¿su hijo quiere irse a estudiar a tal universidad?, listo senador o representante, nosotros cubrimos los costos de estudios de su hijo al otro lado del mundo, que usted necesita tal cosa”, “listo representante, senador, nosotros le damos esto pero usted no puede votar a favor de esto”, “esto lo supimos por el grupo de agenda”. [miembro del gobierno]*

**Actores en la toma de decisiones y la autorregulación**

Los actores de la industria alimentaria a menudo participaron directamente en la formulación de políticas y otras reuniones de alto nivel en Colombia e internacionalmente. El ACTA, por ejemplo, declaró trabajar con el Ministerio de Salud en la reducción del consumo de sal en la población colombiana [A2]. En abril, Colanta participó en el lanzamiento de las “Alianzas Competitivas para la Equidad”, que tiene como objetivo impulsar el desarrollo del país a través de inversiones de corporaciones, en presencia del Presidente Duque y el embajador de EE.UU. en Colombia [A66].

La autorregulación, que afecta indirectamente el proceso de toma de decisiones, al sugerir que son posibles otras alternativas a la reglamentación obligatoria, también fue favorecida por la industria y apoyada por el gobierno [A257, A273]. Las iniciativas impulsadas por la industria alimentaria fueron: el suministro de información nutricional a los consumidores [A152-3]; lo que llamaron "publicidad consciente"; marketing responsable [A155]; una estrategia de reformulación [A12]; la promoción de estilos de vida saludables [A12]. En las entrevistas, varios participantes se mostraron escépticos ante este enfoque:

*“El acuerdo de autorregulación (...) fue [adoptado] en el contexto de evitar un impuesto de bebidas azucaradas y de evitar una serie de propuestas de regulación estatal que en ese momento estaban a punto de hacerse”. [periodista]*

**Estrategias legales**

En el estudio no se encontró información relacionada con las estrategias legales de APC en el análisis de documentos. Sin embargo, los participantes de las entrevistas describieron dos casos en los que las campañas de salud pública dirigidas por organizaciones benéficas fueron impugnadas en el tribunal.

El primer caso ocurrió en el año2016, cuando EducarConsumidores realizó una campaña televisiva sobre los efectos negativos para la salud asociados al consumo de bebidas azucaradas (12). Un participante detalló:

*“EducarConsumidores, cuando se trató de colocar en la televisión colombiana un comercial orientativo sobre los riesgos de la salud por el consumo de bebidas azucaradas, inmediatamente el Grupo Postobón que pertenece a Ardila Lulle, envió una comunicación a la Superintendencia de Industria y Comercio para que retirara el comercial de la televisión. " [defensor de la salud pública]*

Como consecuencia, EducarConsumidores tuvo que detener su campaña (12). Finalmente, la Corte Constitucional de Colombia reconoció que EducarConsumidores tenía derecho a compartir esta información ya que tenía importantes consecuencias para la salud de la población (12). Un participante de las entrevistas explicó que la esposa de un juez de la Corte Constitucional que estaba a cargo del caso fue contratada por Postobón durante ese período [abogado de salud pública].

El segundo caso ocurrió en el 2018, cuando la organización benéfica Red Papaz, que aboga por la protección de los derechos de la niñez y la adolescencia, intentó realizar una campaña denominada “No comas más mentiras” (43). El objetivo de la campaña fue difundir información sobre el consumo de productos comestibles ultra-procesados y sus riesgos para la salud, en particular para niños y adolescentes (43). Red Papaz quiso ejecutar la campaña en los principales canales de televisión del país, pero su solicitud fue rechazada por el Consorcio de Canales Nacionales Privados (Consorcio de Canales Nacionales Privados), que incluye a RCN (43). El caso fue llevado al Tribunal Constitucional en 2019 y finalmente ganó Red Papaz (44).

Un participante explicó que los litigios contra este tipo de campañas eran una práctica bien conocida:

*“El SLAP [Demanda estratégica contra la participación pública] es litigio estratégico para disuadir o distorsionar el debate. Entonces las industrias a veces inician estrategias de litigio, no necesariamente para ganarlas, porque saben que no la van a ganar, sino para silenciar una voz o para atemorizar a la sociedad civil”. [defensor de la salud pública]*

**Estrategias discursivas**

Se identificaron 138 ejemplos con el análisis de datos públicos, en los que la industria alimentaria utilizó una amplia gama de argumentos como parte de sus estrategias discursivas de la APC. Los participantes en las entrevistas también describieron estrategias discursivas.

**Papel de la industria en la economía**

En Colombia, la creación de empleo por parte de la industria alimentaria a menudo se enmarcaba como una contribución no solo a la economía [A232], sino, lo que es más importante, como un factor central para el desarrollo social. En ocasiones, esto se debatió como parte de las iniciativas de responsabilidad social empresarial de los actores de la industria alimentaria [A27]. La ANDI por ejemplo declaró: “La industria alimentaria en Colombia es un motor de desarrollo económico y social: Gran generadora de empleo formal (260.000 trabajadores); Más de 65.000 empresas; Gran exportador: más de USD900 millones a 129 países. ¡Creamos bienestar económico y social!” [A25].

La industria alimentaria también utilizó el argumento económico para criticar las políticas públicas propuestas que impactarían sus productos y actividades. Tras la supresión de un subsidio a las bebidas azucaradas, Coca-Cola dijo que perdió ingresos y tuvo que eliminar 177 empleos, y como consecuencia de estas pérdidas de dinero, la empresa decidió dejar de patrocinar a la selección colombiana de fútbol y declaró que la decisión tuvo “efectos contraproducentes para la economía” [A44].

**Encuadre del debate en salud pública y nutrición**

En sus esfuerzos por enmarcar el debate sobre nutrición en salud pública en el país, los actores de la industria alimentaria promovieron su papel central y sus esfuerzos en la prevención y control de las ENT y otros temas relacionados con la dieta. Por ejemplo, Alquería explicó que su distribución de productos a los bancos de alimentos fue crucial para el país:

*“Somos conscientes de la importancia de nuestro papel en la cadena alimentaria y de nuestro compromiso con la erradicación del hambre en Colombia” [A71].*

Otros actores presentaron argumentos similares:

*“Nestlé ha contribuido a mejorar la calidad de vida y asegurar un futuro más saludable para los niños” [A186].*

Los actores de la industria alimentaria abogaron por la autorregulación, incluido el uso de un sistema de etiquetado frontal de los alimentos, como se describió anteriormente, y por otras iniciativas voluntarias, incluida la promoción de la educación sobre nutrición y actividad física, en lugar de la introducción de nuevas políticas públicas [A29, A33-4, A196, A256, A273].

Se identificaron dos iniciativas de la industria alimentaria: cada una tenía un sitio web dedicado y una cuenta de Twitter. El primero fue “Decido lo que como” (“Yo decido lo que como”) [A38, A47]. La iniciativa fue desarrollada por la Fundación Éxito, Nestlé y otros actores de la industria alimentaria [A138] y las fuentes de información citadas fueron actores de la industria [A203]. La segunda iniciativa fue “Bebidas de tu lado” donde la ANDI promovió las cinco iniciativas de autorregulación adoptadas por los actores de la industria alimentaria en Colombia, como se describió anteriormente [A12, A150]. En sus mensajes, en estas plataformas y otros medios, los actores de la industria alimentaria promovieron particularmente la responsabilidad personal y parental, las dietas equilibradas y la actividad física [A47, A49, A52-3, A149, A156, A200, A254, A259-67, A274 y entrevistas].

En las entrevistas, un participante sugirió:

*“En un país como Colombia, que es víctima de un conflicto interno que no ha terminado y ya tiene más de un siglo de duración, entonces este tema de la culpa [personal] juega mucho, tiene mucho poder". [defensor de la salud pública]*

**Discusión**

Los resultados del estudio revelan que la industria alimentaria es un actor destacado e influyente en Colombia. En esta investigación se encontraron 275 ejemplos de prácticas de APC para la industria alimentaria, utilizando los datos disponibles públicamente. Los participantes describieron ejemplos adicionales, incluidos nuevos datos sobre las estrategias legales, y proporcionaron un análisis crítico de estas acciones y argumentos de la industria alimentaria.

Se encontró evidencia de que los actores de la industria alimentaria construyeron alianzas con las comunidades, el gobierno (nacional y local) y los medios de comunicación. Las interacciones entre la industria alimentaria y los actores del gobierno, la academia y los medios de comunicación, entre otros, podrían significar que la industria obtenga credibilidad por asociación (9). En un país afectado por un conflicto armado y donde algunos segmentos de la población aún carecen de acceso a la infraestructura básica, la alimentación y la educación, nuestros resultados muestran que la industria alimentaria a menudo se describe como una contribución a la prosperidad del país, al menos en el corto plazo. Esto significa que las inversiones y el empleo, que son posibles gracias a las corporaciones con el apoyo del gobierno y quizás del público, pueden tener prioridad sobre los objetivos de salud pública. Distintos actores de la nutrición en salud pública que abogan por el derecho humano a la alimentación a menudo se sienten inseguros en sus posiciones al criticar los productos o acciones de esa industria.

La industria trató de influir en la ciencia en temas de nutrición y relacionados con la dieta en Colombia, a través de su producción interna y difusión de la ciencia y el uso de terceros como ILSI, que a su vez tuvo un impacto directo en la política.

En Colombia, los actores de la industria participaron directamente en la formulación de políticas públicas en el país. La industria también estaba promoviendo la autorregulación, que es un enfoque estándar de la industria para evitar la regulación gubernamental que ha demostrado ser ineficaz (45–47). Las organizaciones de la sociedad civil han monitoreado los compromisos voluntarios asumidos por la industria alimentaria en el año 2016, que tenían como objetivo limitar la venta de productos nocivos para la salud en las escuelas [A144]. Concluyeron que las empresas alimentarias no cumplieron sus objetivos iniciales (48). La industria respondió con el lanzamiento de otra iniciativa de autorregulación en septiembre del año 2019 en presencia del Ministro de Salud (49).

Se encontraron evidencias del uso de estrategias legales en Colombia en el análisis de documentos y además en las entrevistas se describieron algunos ejemplos de litigio. Esto se explica por el hecho de que solo se recopilaron documentos publicados en el año 2019, mientras los entrevistados discutieron casos antiguos de los años 2016 y 2018.

La industria alimentaria en Colombia está utilizando estrategias discursivas, donde la industria se presentó como un actor económico esencial en el país y enmarcó el debate sobre las ENT y otros temas relacionados con la dieta.

Este fue el primer estudio de la APC de la industria alimentaria en América Latina. Los resultados son consistentes con los hallazgos de otros estudios sobre APC de la industria alimentaria a nivel mundial, donde todas las estrategias de APC son utilizadas por grandes actores económicos para influir en las políticas, la investigación y la práctica (16,23,28,50). Sin embargo, en Colombia, la proximidad entre la industria, el gobierno y los medios de comunicación es particularmente evidente y permanece en gran parte incuestionable. La influencia de las poblaciones vulnerables en las comunidades, incluso en áreas que carecen del apoyo del gobierno, y las amenazas a las organizaciones de la sociedad civil también son llamativas y preocupantes. Las prácticas de APC de la industria alimentaria podrían facilitar la distribución de productos comestibles ultra-procesados, con el supuesto, por ejemplo, de que dichos productos pueden ayudar a abordar el hambre, como fue el caso de las bebidas fortificadas distribuidas en el extremo norte del país a un segmento vulnerable del país. población. Estas iniciativas de alivio del hambre también se han utilizado en otras partes del mundo (51). Por lo tanto, estas prácticas de APC pueden representar un riesgo para la salud de la población y la de los niños en particular, ya que el consumo de productos comestibles ultra-procesados se ha asociado con el desarrollo de ENT (52,53), pero también con la protección de los derechos humanos fundamentales. a la salud y la alimentación adecuada, como lo reconoce el Relator Especial de las Naciones Unidas sobre el derecho a la salud (54).

Este estudio también se basa en la creciente literatura sobre los determinantes comerciales de la salud, que se centra en cómo las corporaciones comercializan y presionan por productos nocivos para la salud (19, 55). Al aplicar un análisis de APC en Colombia, este estudio identifica y expone las prácticas de las corporaciones, lo que podría ayudar a académicos, defensores y funcionarios gubernamentales a contrarrestar la interferencia de la industria y ayudar a preparar, promulgar e implementar políticas de salud pública basadas en evidencia (56). Dado que los determinantes comerciales ven a la industria como el vector de la enfermedad (55), la investigación futura debería explorar comparaciones entre industrias y políticas para identificar patrones y tendencias en evolución en la actividad de la industria y la interferencia de políticas.

Este estudio tiene algunas limitaciones. En las entrevistas, se pudo tener un mejor acceso a los actores de la sociedad civil, en comparación con los actores de la industria alimentaria y el gobierno, universidades y asociaciones profesionales. También información limitada disponible para el público con respecto a las interacciones de estas personas con la industria alimentaria. Esto puede deberse al hecho de que estas interacciones no son conocidas por el público, sino que ocurren en espacios privados, como reuniones personales y a través de correos electrónicos o llamadas telefónicas, y que estas personas no están necesariamente dispuestas a discutir críticamente estas interacciones. Además, las búsquedas de información disponible públicamente a los datos publicados en los últimos meses y a un número limitado de actores de la industria alimentaria, debido a limitaciones de tiempo. Los estudios futuros podrían cubrir un período de tiempo más largo e incluir actores adicionales.

Finalmente, existen soluciones para abordar y prevenir la influencia negativa de la industria alimentaria en la política, la investigación y la práctica de salud pública en Colombia y, en el exterior, como se detalla recientemente en una revisión de alcance (56). Además, se notó, la existencia y disponibilidad, en línea, de un registro de cabilderos (lobistas) en el país, pero no se ha actualizado desde el 2014. También existe una ley que prohíbe a los miembros de los gobiernos trabajar en un sector que antes regulaban, pero Los participantes explicaron que la ley no necesariamente se implementa (artículo 3 de la Ley 1474 de 2011). En Colombia, el “Colectivo de Abogados José Alvear Restrepo” (CAJAR), lanzó un 'Pacto por la transparencia en las políticas de salud pública y contra la injerencia en los derechos [humanos]' (57). El Pacto propuso una serie de acciones que podrían ayudar a reducir la interferencia de la industria alimentaria en el país [Archivo 6] (57). Además, la protección de la salud pública, más allá de las políticas, de la influencia indebida de las corporaciones debe abordarse en Colombia.

**Conclusiones**

En Colombia, la industria alimentaria ha penetrado en muchas instituciones e interactúa estrechamente con personas en las políticas públicas, las comunidades, la investigación y los medios de comunicación. Es fundamental que estos actores comprendan los riesgos asociados con la APC y los determinantes comerciales de la salud, y que se desarrollen e implementen soluciones para abordar la influencia de los intereses creados y motivados por las ganancias de la industria alimentaria.

**Declaraciones**

**Aprobación ética y consentimiento para participar**

Este estudio, fue parte de un proyecto más amplio sobre la industria alimentaria en América Latina, se llevó a cabo de acuerdo con los lineamientos establecidos en la Declaración de Helsinki y todos los procedimientos que involucraron a los participantes del estudio de investigación fueron aprobados por el comité de ética de la Escuela de Salud Pública, Universidad de Sao Paulo, Brasil (número de proyecto 07944118.7.0000.5421).

Los participantes firmaron un formulario de consentimiento informado ético antes de participar en el estudio.

**Consentimiento para la publicación**

Se obtuvo el consentimiento por escrito de los participantes para publicar los datos, en condiciones de anonimato y confidencialidad.

**Disponibilidad de datos y materiales**

Todos los datos del dominio público recopilados durante este estudio están disponibles con este manuscrito en el Archivo 4. Los datos de las entrevistas están disponibles del autor correspondiente a solicitud razonable.

**Conflicto de intereses**

Los autores declaran que no tienen intereses en competencia.

**Fondos**

MM recibió una beca de la Fundación de Investigación de São Paulo (FAPESP), Brasil (número de beca 2017 / 24744-0). MM obtuvo financiación inicial de la Facultad de Ciencias de la Salud (FHS) de la Universidad Americana de Beirut (AUB) para MM, como parte de una subvención financiada por el Centro Internacional de Investigaciones para el Desarrollo (IDRC). Este financiamiento apoyó su trabajo de campo en Colombia y Chile en 2019. En 2018/2019, MM actuó como consultora de la Campaña para Niños Libres de Tabaco (CFTFK), el Instituto Brasileño de Defensa del Consumidor - IDEC, la Fundación Heart and Stroke de Barbados. Inc., la Coalición del Caribe Saludable - HCC y la Organización Panamericana de la Salud - OPS / oficina regional de las Américas para la Organización Mundial de la Salud (OMS). FBS recibió una beca del Consejo Nacional de Desarrollo Científico y Tecnológico (CNPq), Brasil (número de subvención 309514 / 2018-5). Los patrocinadores no participaron en el diseño del estudio, la recopilación y el análisis de datos, la decisión de publicar o la preparación del manuscrito. Los autores son los únicos responsables de las opiniones, hipótesis y conclusiones o recomendaciones expresadas en esta publicación.

**Contribuciones de los autores**

MM dirigió el diseño del estudio, la recopilación de datos, el análisis y la redacción del manuscrito. FBS contribuyó al diseño del estudio. DAGC, GC y EMPT contribuyeron al diseño del estudio, la recopilación y el análisis de datos. EC contribuyó al análisis de datos. Todos los autores contribuyeron a la redacción del manuscrito y leyeron y aprobaron el manuscrito final.

**Agradecimientos**

Los autores desean agradecer a Cora-Lee Leblanc, de la Universidad de Moncton, Canadá, por sus contribuciones en las primeras etapas de este estudio. Los autores también desean agradecer a sus entrevistados su participación en este estudio.

**Material adicional**

• Archivo 1 clasificación CPA.docx: Marco conceptual para categorizar la actividad política empresarial de la industria alimentaria

• Archivo 2 COREQ.docx: Lista de verificación de criterios consolidados para la presentación de informes de investigación cualitativa (COREQ)

• Archivo 3 3 Sources.docx: Fuentes de información para identificar la política empresarial de la industria alimentaria en Colombia

• Archivo 4 Data.docx: datos recopilados a partir de información disponible públicamente

• Archivo 5 Guide.docx: Guía de entrevista (español)

• Archivo 6 Conclusiones Acciones CPA.docx: Acciones propuestas en el “Pacto por la transparencia en las políticas públicas de salud y contra la injerencia en los derechos [humanos]”, adaptado del Colectivo de Abogados José Alvear Restrepo

• Archivo 7 Translation.docx: versión en español del artículo.

**Referencias**

1. World Health Organization. Global Strategy on Diet, Physical Activity and Health. Geneva: World Health Organization; 2004.

2. Congreso de la Republica de Colombia - Cámara de Representantes. Control de la obesidad - Proyecto de Ley 214 de 2018 [Internet]. [cited 2019 Oct 4]. Available from: http://www.camara.gov.co/control-de-la-obesidad-0

3. El Congreso de Colombia. Derecho del Bienestar Familiar [LEY 1355 DE 2009 (octubre 14) Diario Oficial No. 47.502 de 14 de octubre de 2009] [Internet]. 2009 [cited 2019 Nov 19]. Available from: https://www.icbf.gov.co/cargues/avance/docs/ley_1355_2009.htm

4. World Health Organization. Noncommunicable Diseases (NCD) Country Profiles - Colombia [Internet]. 2018 [cited 2019 Sep 24]. Available from: https://www.who.int/nmh/countries/2018/col_en.pdf?ua=1

5. Institute for Health Metrics and Evaluation (IHME). Institute for Health Metrics and Evaluation - Colombia profile [Internet]. 2018 [cited 2020 Aug 19]. Available from: http://www.healthdata.org/colombia

6. Minsalud. Encuesta Nacional de Situación Nutricional de Colombia – ENSIN 2015 [Internet]. 2015 [cited 2019 Nov 22]. Available from: http://www.ensin.gov.co/#

7. Liga contra el Silencio. Donaciones dulces aceitan la política en Colombia . Liga contra el Silencio. 2019 Aug 29;

8. Liga contra el Silencio. Así fue el lobby en el Congreso contra la Ley de Etiquetado. Liga contra el Silencio. 2019 Jul 11;

9. Gómez L, Jacoby E, Ibarra L, Lucumí D, Hernandez A, Parra D, et al. Sponsorship of physical activity programs by the sweetened beverages industry: public health or public relations? Rev Saude Publica. 2011 Apr;45(2):423–427.

10. Cortés C, Peñarredonda JL. Numero 18: En el debate sobre la comida chatarra: derrota de la sociedad civil a manos de la industria. Fundación Heinrich Böll Oficina Bogotá; 2019 May.

11. Sandoval Salazar MY, Orjuela R, Vivas D, Erazo A. Acciones de la industria ante medidas de salud pública para disminuir consumos dañinos para la salud - Interferencia de la industria al impuesto a las bebidas azucaradas. Asociación Colombiana de Educación al Consumidor Educar Consumidores; 2017.

12. Vivas Mosquera DC. Crónica de una censura - y Sus Implicaciones Respecto de los Derechos a la Salud, la Alimentación Adecuada y los Derechos de los Consumidores. Asociación Colombiana de Educación al Consumidor Educar Consumidores; 2018 Jan.

13. Sandoval Salazar MY. Interferencia de la industria en las propuestas de implementación de sellos frontales de advertencias 2017-2018. Asociación Colombiana de Educación al Consumidor Educar Consumidores; 2019.

14. Congreso de la Republica de Colombia. Proyecto de Ley 019 de 2017 - Cámara de Representantes. Jul 25, 2016.

15. Liga Contra el Silencio. El dulce y ultra-procesado círculo que rodea a Iván Duque*. Liga Contra el Silencio. 2019 Sep 26;

16. Mialon M, Swinburn B, Sacks G. A proposed approach to systematically identify and monitor the corporate political activity of the food industry with respect to public health using publicly available information. Obes Rev. 2015 Jul;16(7):519–530.

17. Mialon M, Julia C, Hercberg S. The policy dystopia model adapted to the food industry: the example of the Nutri-Score saga in France. World Nutrition. 2018;9(2):109–120.

18. Hillman AJ, Hitt MA. Corporate political strategy formulation: A model of approach, participation, and strategy decisions. The Academy of Management Review. 1999 Oct;24(4):825.

19. Kickbusch I, Allen L, Franz C. The commercial determinants of health. Lancet Glob Health. 2016;4(12):e895–e896.

20. Millar JS. The corporate determinants of health: how big business affects our health, and the need for government action! Can J Public Health. 2013 May 14;104(4):e327–9.

21. McKee M, Stuckler D. Revisiting the corporate and commercial determinants of health. Am J Public Health. 2018 Jul 19;108(9):1167–1170.

22. UK Health Forum. Public health and the food and drinks industry: The governance and ethics of interaction - Lessons from research, policy and practice [Internet]. 2018 [cited 2018 Sep 8]. Available from: http://bit.ly/2mUqwNr

23. Mialon M, Gomes F da S. Public health and the ultra-processed food and drink products industry: corporate political activity of major transnationals in Latin America and the Caribbean. Public Health Nutr. 2019 Mar 12;22(10):1898–1908.

24. Tong A, Sainsbury P, Craig J. Consolidated criteria for reporting qualitative research (COREQ): a 32-item checklist for interviews and focus groups. Int J Qual Health Care. 2007 Dec;19(6):349–357.

25. Mialon M, Mialon J. Analysis of corporate political activity strategies of the food industry: evidence from France. Public Health Nutr. 2018 Jul 12;1–15.

26. Mialon M, Mialon J. Corporate political activity of the dairy industry in France: an analysis of publicly available information. Public Health Nutr. 2017 Sep;20(13):2432–2439.

27. Mialon M, Swinburn B, Allender S, Sacks G. Systematic examination of publicly-available information reveals the diverse and extensive corporate political activity of the food industry in Australia. BMC Public Health. 2016 Mar 22;16:283.

28. Mialon M, Swinburn B, Wate J, Tukana I, Sacks G. Analysis of the corporate political activity of major food industry actors in Fiji. Global Health. 2016 May 10;12(1):18.

29. Jaichuen N, Phulkerd S, Certthkrikul N, Sacks G, Tangcharoensathien V. Corporate political activity of major food companies in Thailand: an assessment and policy recommendations. Global Health. 2018;14(115).

30. Tselengidis A, Östergren P-O. Lobbying against sugar taxation in the European Union: Analysing the lobbying arguments and tactics of stakeholders in the food and drink industries. Scand J Public Health. 2019 Jul;47(5):565–575.

31. The International Food and Beverages Alliance. The IFBA - Our members [Internet]. 2018. Available from: https://ifballiance.org/about#vic-widget-27-container-anchor

32. Mialon M, Gaitan Charry DA, Cediel G, Crosbie E, Scagliusi FB, Perez Tamayo EM. I had never seen so many lobbyists ’: food industry political practices during the development of a new nutrition front-of-pack labelling system in Colombia. Public Health Nutr. 2020 Aug 21;1–9.

33. La Liga contra el Silencio. Postobón hace pruebas de laboratorio con niños en La Guajira. VICE. 2018 Feb 13;

34. Liga Contra el Silencio. El escándalo de Kufu sin responsables dos años después. Liga Contra el Silencio. 2019 Dec 10;

35. Lawyers Without Borders Canada. The Peace Process in Colombia . Lawyers Without Borders Canada; 2016.

36. Cediel G, Perez E, Gaitan D, Sarmiento O, Gonzalez L. Association of all forms of malnutritionand socioeconomic status, educational level and ethnicity in Colombian childrenand non-pregnant women. Public Health Nutr.

37. Jacobs A, Richtel M. She Took On Colombia’s Soda Industry. Then She Was Silenced. The New York Times. 2017;

38. Steele S, Ruskin G, Sarcevic L, McKee M, Stuckler D. Are industry-funded charities promoting “advocacy-led studies” or “evidence-based science”?: a case study of the International Life Sciences Institute. Global Health. 2019 Jun 3;15(1):36.

39. Greenhalgh S. Soda industry influence on obesity science and policy in China. J Public Health Policy. 2019 Mar;40(1):5–16.

40. World Health Organization. The Tobacco Industry and Scientific Groups ILSI: A Case Study. 2001;

41. Liga Contra el Silencio. Una multinacional de la ciencia en Colombia para los intereses de la industria. Liga Contra el Silencio. 2019 Oct 31;

42. International Life Science Institute. ILSI / Estudio Latinoamericano de Nutrición y Salud (ELANS) [Internet]. [cited 2019 Nov 7]. Available from: https://ilsi.org/elans/

43. Dejusticia. Dejusticia intervino ante la Corte Constitucional en defensa del derecho a recibir información sobre ultraprocesados | Dejusticia [Internet]. 2019 [cited 2019 Nov 26]. Available from: https://www.dejusticia.org/dejusticia-intervino-ante-la-corte-constitucional-en-defensa-del-derecho-a-recibir-informacion-sobre-ultraprocesados/

44. Red Papaz ganó pulso a Caracol y RCN para emitir comercial contra' ' ’comida chatarra. El Pais. 2019 Apr 9;

45. Kunkel DL, Castonguay JS, Filer CR. Evaluating Industry Self-Regulation of Food Marketing to Children. Am J Prev Med. 2015 Aug;49(2):181–187.

46. Ronit K, Jensen JD. Obesity and industry self-regulation of food and beverage marketing: a literature review. Eur J Clin Nutr. 2014 Jul;68(7):753–759.

47. Lacy-Nichols J, Scrinis G, Carey R. The politics of voluntary self-regulation: insights from the development and promotion of the Australian Beverages Council’s Commitment. Public Health Nutr. 2019 Aug 9;1–12.

48. Liga Contra el Silencio. Empresas de bebidas azucaradas incumplen acuerdos de venta en colegios. Liga Contra el Silencio. 2019 May 23;

49. ANDI. Icontec será el organismo verificador del cumplimiento de los compromisos de autorregulación de la Industria de Bebidas en Colombia [Internet]. 2019 [cited 2019 Nov 20]. Available from: http://www.andi.com.co/Home/Noticia/13473-icontec-sera-el-organismo-verificador-d

50. Jaichuen N, Phulkerd S, Certthkrikul N, Sacks G, Tangcharoensathien V. Corporate political activity of major food companies in Thailand: an assessment and policy recommendations. Global Health. 2018 Nov 22;14(1):115.

51. Mialon M, Crosbie E, Sacks G. Mapping of food industry strategies to influence public health policy, research and practice in South Africa. Int J Public Health. 2020 Jul 29;

52. Monteiro CA, Cannon G, Moubarac J-C, Levy RB, Louzada MLC, Jaime PC. The UN Decade of Nutrition, the NOVA food classification and the trouble with ultra-processing. Public Health Nutr. 2018 Jan;21(1):5–17.

53. Fiolet T, Srour B, Sellem L, Kesse-Guyot E, Allès B, Méjean C, et al. Consumption of ultra-processed foods and cancer risk: results from NutriNet-Santé prospective cohort. BMJ. 2018 Feb 14;360:k322.

54. Grover A. Report to the Human Rights Council (main focus: unhealthy foods and non-communicable diseases). New York: United Nations; 2014.

55. Mialon M. An overview of the commercial determinants of health. Global Health. 2020 Aug 17;16(1):74.

56. Mialon M, Vandevijvere S, Carriedo-Lutzenkirchen A, Bero L, Gomes F, Petticrew M, et al. Mechanisms for addressing and managing the influence of corporations on public health policy, research and practice: a scoping review. BMJ Open. 2020 Jul 19;10(7):e034082.

57. El Colectivo de Abogados José Alvear Restrepo – CAJAR. Firma el pacto [Internet]. Dulce Veneno. 2019 [cited 2019 Nov 4]. Available from: http://eldulceveneno.org/firma-el-pacto/
