# Supplementary material for: “The architecture of the state was transformed in favour of the interests of companies”: corporate political activity of the food industry in Colombia
Source: Global Health. 2020 Oct 12;16:97. doi: 10.1186/s12992-020-00631-x (PMC7552360; doi:10.1186/s12992-020-00631-x)
Supplement: Supplementary file 7 — Additional file 7. Actions proposed in the “Pact for transparency in public health policies and against interference with [human] rights”, adapted from the Colectivo de Abogados José Alvear Restrepo. [file 12992_2020_631_MOESM7_ESM.docx]

Additional file 7: Actions proposed in the “Pact for transparency in public health policies and against interference with [human] rights”, adapted from the Colectivo de Abogados José Alvear Restrepo (1)

| **Suggested actions** |
| --- |
| 1. the regulation of lobbying related to public policies in human rights |
| 1. the adoption of a protocol for the management of relationships between public officials and representatives of the tobacco, sugary drinks, or ultraprocessed food industries |
| 1. the establishment of a public registry of private funding for academic research |
| 1. the declaration of conflicts of interest for research and press releases funded by the industry |
| 1. the rejection of economic arguments by the industry when those could refrain the government from adopting measures that seek to improve public health outcomes, promote healthy eating, promote healthy environment and basic sanitation, children's rights, and labor rights |
| 1. an independent monitoring and evaluation of the corporate social responsibility initiatives developed by companies |
| 1. the regulation of these initiatives when they relate to human rights |
| 1. sanctions for the publication of fake news on health, food, environment, basic sanitation, the rights of children, and labor rights, whose purpose is to block, hinder, postpone, delay or hinder the adoption of public policies targeted at the general interest, at the safeguarding of human rights and at the improvement of public health outcomes. |
| 1. the regulation by the constitution and international human rights treaties of political decision spaces related to public health, food, environment, basic sanitation, children's rights, and labor rights |
| 1. a minimum period of abstention from the exercise of private activity that may involve a conflict of interest for those who previously worked in public offices, both in management and advisory work in government agencies. |

1. El Colectivo de Abogados José Alvear Restrepo – CAJAR. Firma el pacto [Internet]. Dulce Veneno. 2019 [cited 2019 Nov 4]. Available from: http://eldulceveneno.org/firma-el-pacto/
